# Supplementary figures and images for: Mutations accumulated in the Spike of SARS-CoV-2 Omicron allow for more efficient counteraction of the restriction factor BST2/Tetherin
Source: PLoS Pathog. 2024 Jan 8;20(1):e1011912. doi: 10.1371/journal.ppat.1011912 (PMC10798645; doi:10.1371/journal.ppat.1011912)

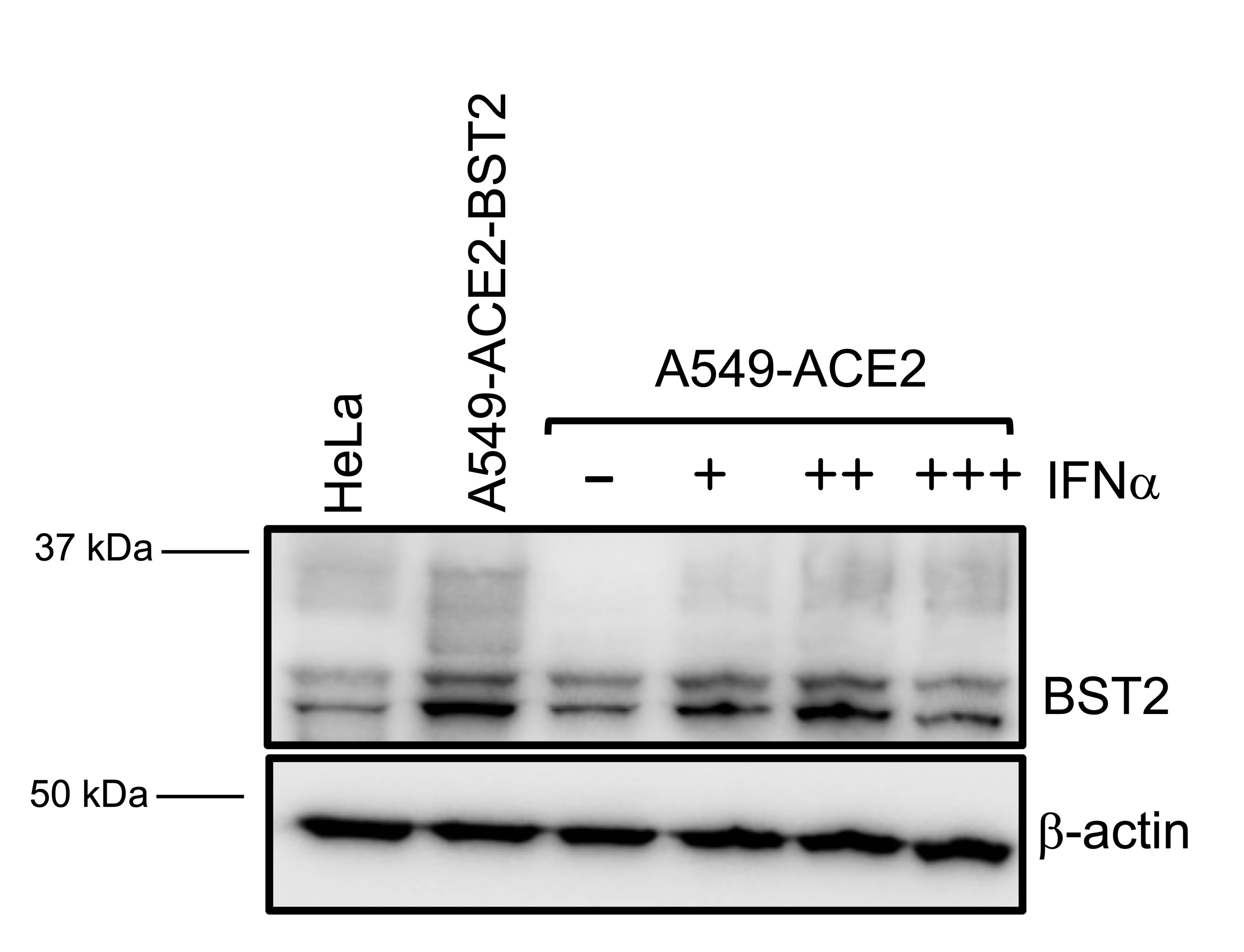

Supplement: S1 Fig — The levels of BST2 in A549-ACE2 cells engineered to constitutively express BST2 were compared to HeLa cells, which express BST2 endogenously, and parental A549-ACE2 cells treated with IFNα. For this, A549-ACE2 cells were treated with 100, 1,000 and 10,000 U/mL of IFNα2. Cells were harvested 24 hours later and analyzed by western blot. (TIFF) [file ppat.1011912.s001.tiff]

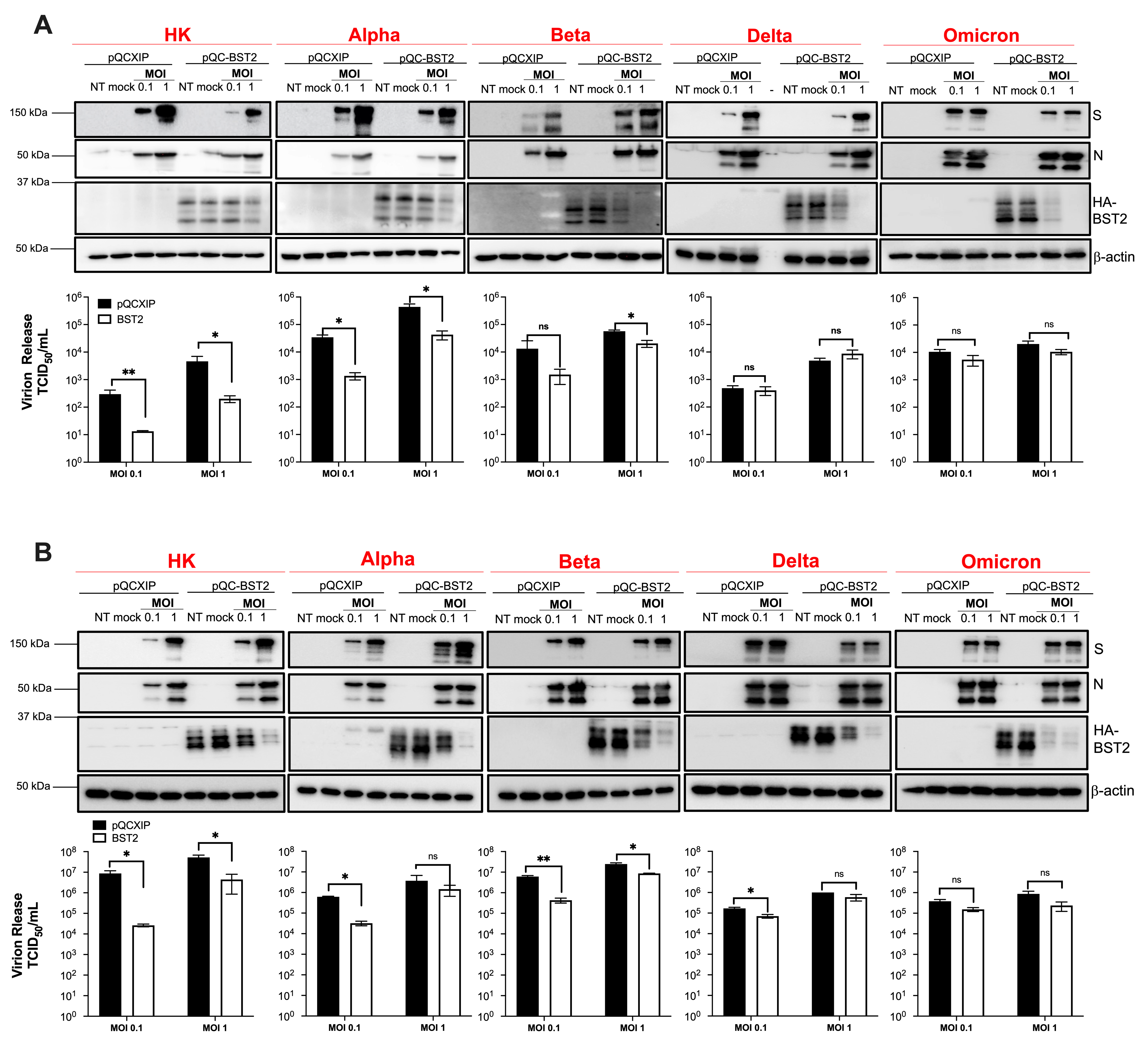

Supplement: S2 Fig — (A) HEK293T-ACE2 and (B) A549-ACE2 cells stably expressing pQCXIP or pQCXIP-BST2 were infected with SARS-CoV-2 HK, Alpha, Beta, Delta, or Omicron variants at MOI = 0.1 or 1. Twenty-four hours post-infection, the levels of BST2 and virus proteins were measured by western blot, and infectious virion production was measured by TCID50 (bottom panels). *: p<0.05, **: p<0.01, ns: not significant. Blots are representative of 3 biological replicates. Data correspond to the mean and SEM of 3 independent experiments. (TIFF) [file ppat.1011912.s002.tiff]

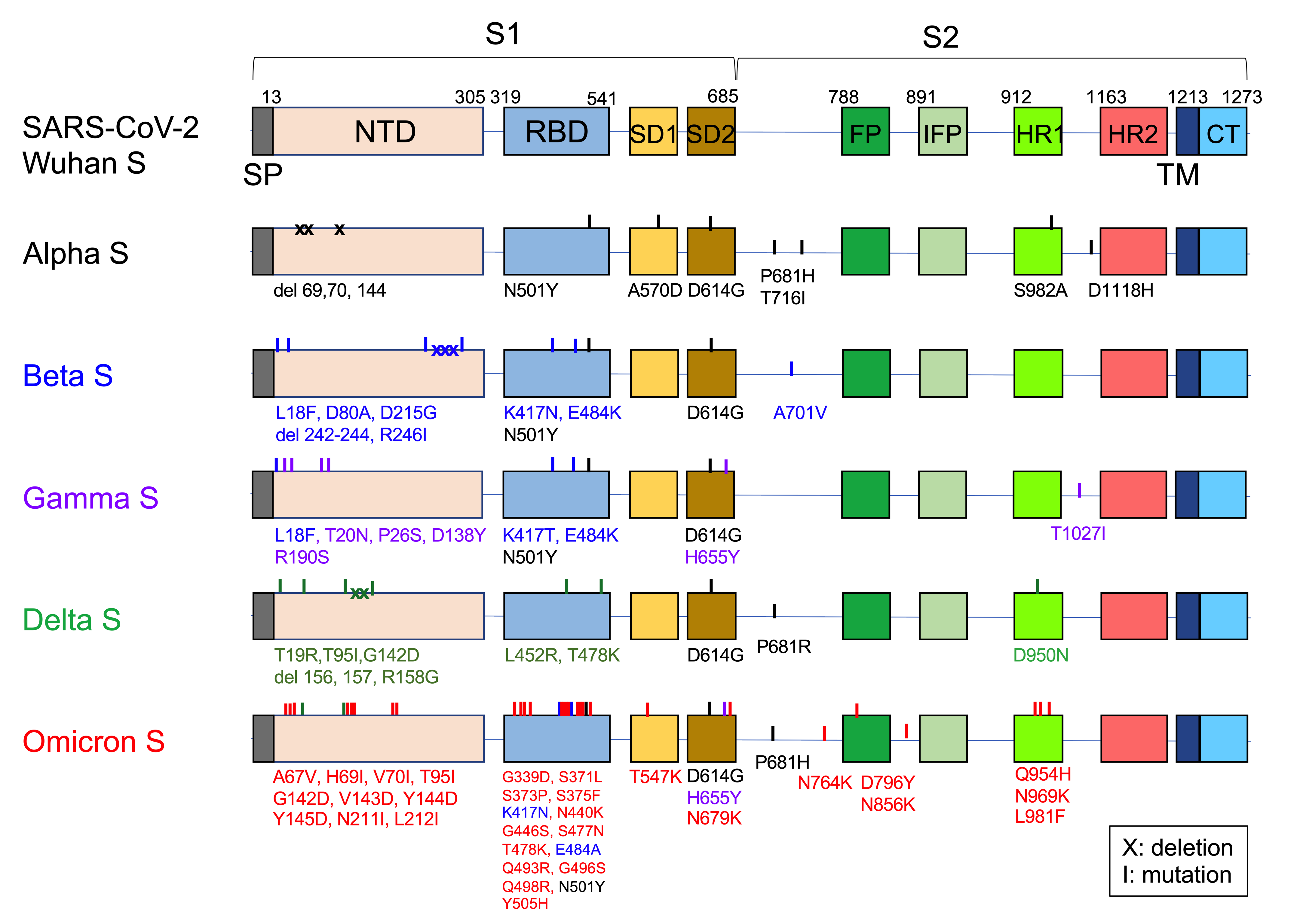

Supplement: S3 Fig — Schematic representation of the SARS-CoV-2 Spike. Mutations accumulated in variants of concern compared to the Wuhan sequence (NC_045512) are indicated. Spike sequences were obtained from NCBI GenBank: Alpha B.1.1.7: MZ344997.1, Beta B.1.351: MW598419.1, Gamma P.1: MW642250.1, Delta B.1.617.2: MZ009823.1, Omicron B.1.1.529: OL672836.1. (TIFF) [file ppat.1011912.s003.tiff]

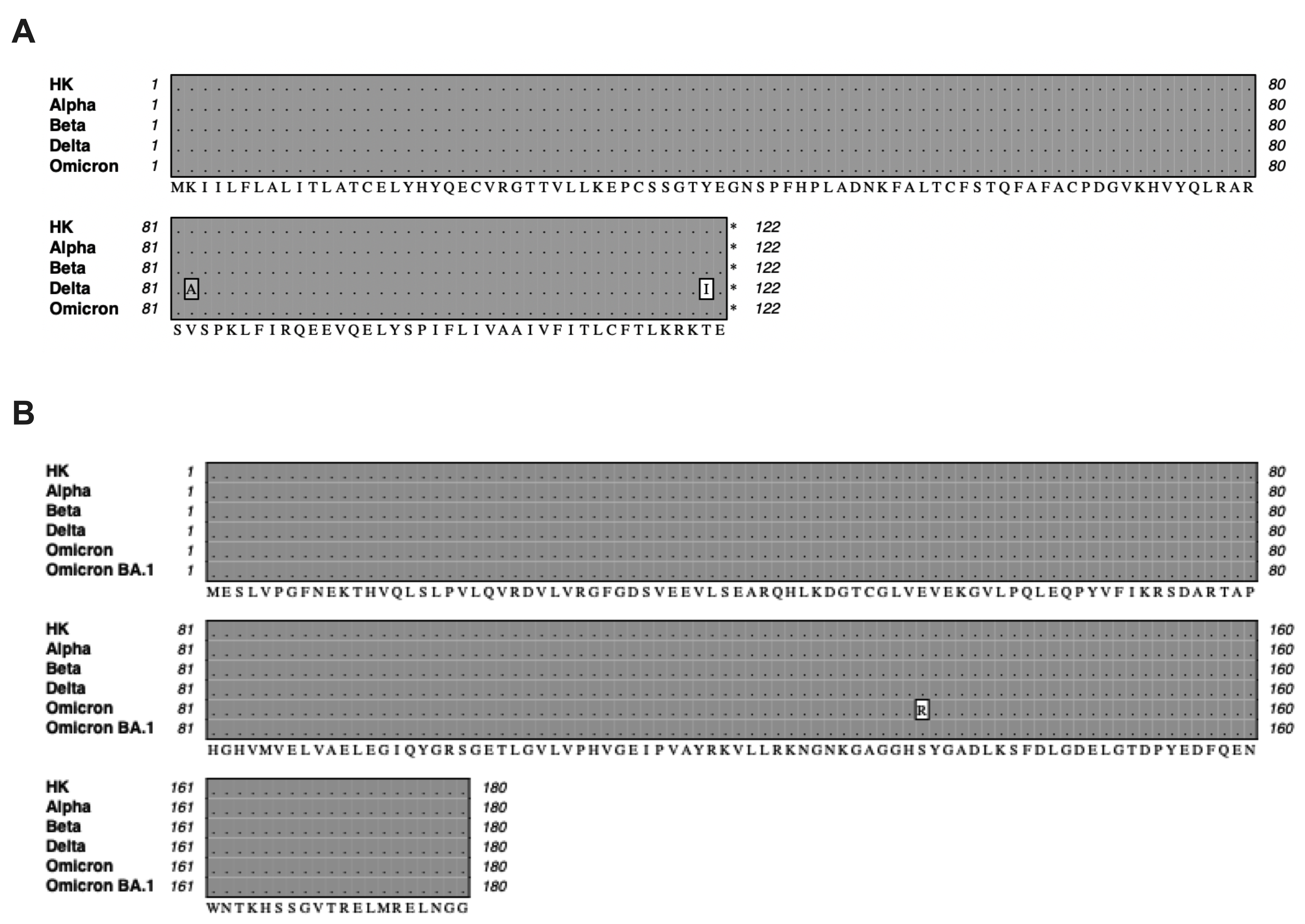

Supplement: S4 Fig — Sequence alignments of ORF7a (A) and NSP1 (B) across variants of concern. SARS-CoV-2 ORF7a and NSP1 sequences were obtained from NCBI GenBank; Wuhan-Hu-1: NC_045512.2, Alpha B.1.1.7: MZ344997.1, Beta B.1.351: MW598419.1, Delta B.1.617.2: MZ009823.1, Omicron B.1.1.529: OL672836.1 (TIFF) [file ppat.1011912.s004.tiff]

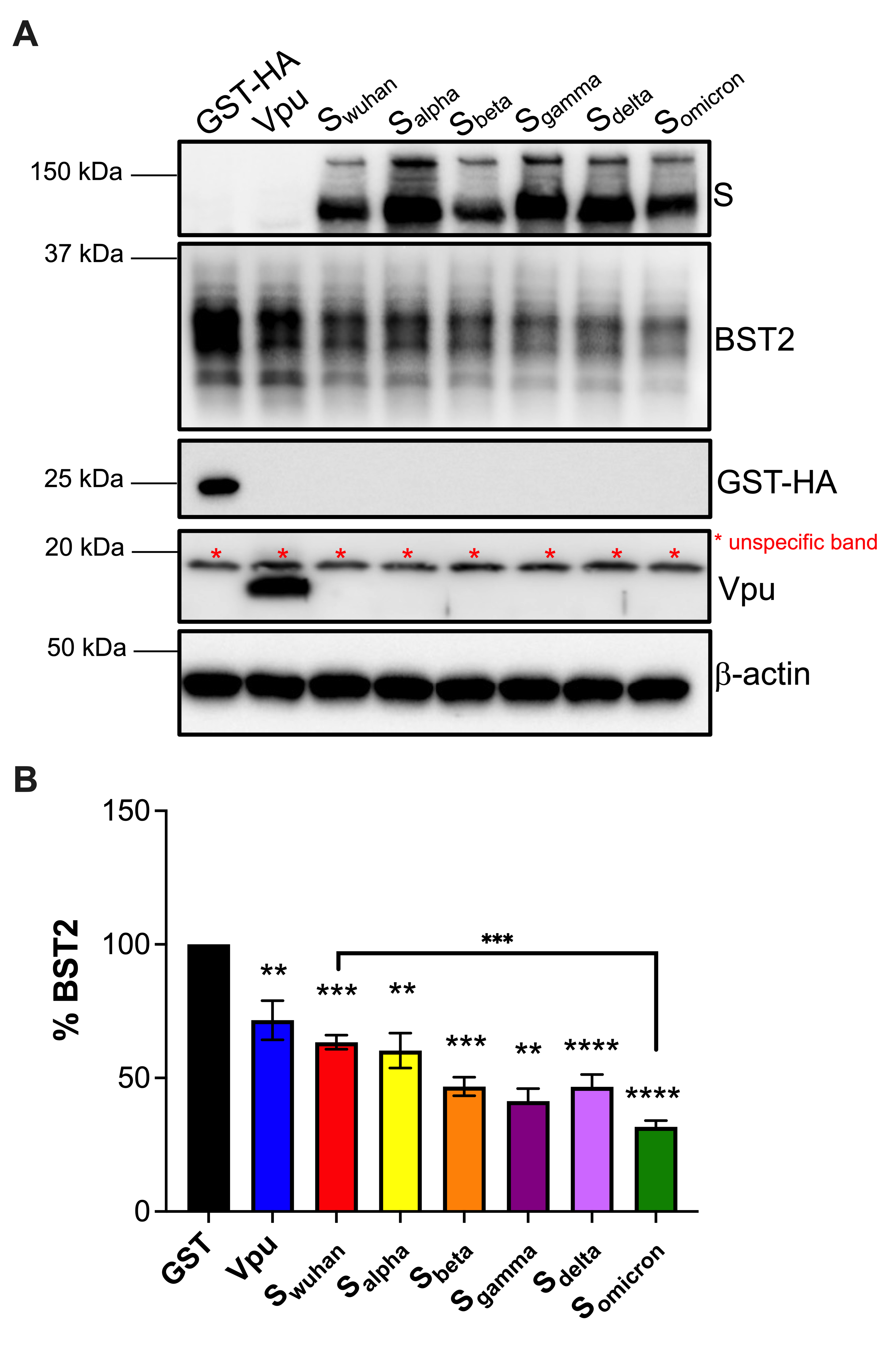

Supplement: S5 Fig — (A) HEK293T-ACE2 cells stably expressing BST2 were transfected with plasmids encoding the Spike gene from each of the indicated variants of concern. BST2 and Spike levels were measured by western blot. (B) Relative BST2 expression was calculated by densitometry analyses, normalized to actin, and expressed as the percentage of BST2. Red asterisks indicate unspecific bands. **: p<0.01, ***: p<0.001, ****: p<0.0001. Blots are representative of 3 biological replicates. Data correspond to the mean and SEM of 3 independent experiments. (TIFF) [file ppat.1011912.s005.tiff]

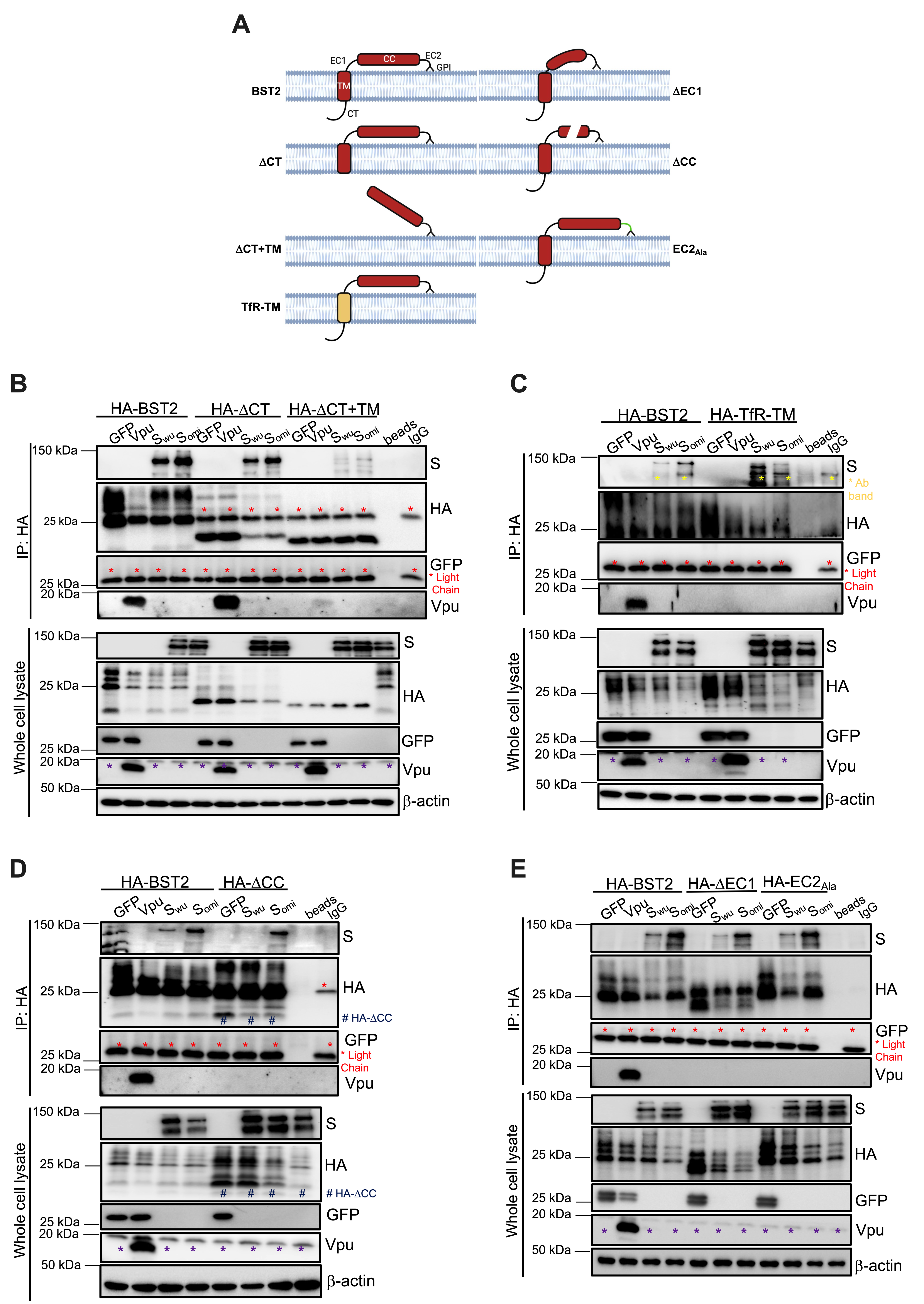

Supplement: S6 Fig — Extended data for Fig 5. (A) Diagram of the architecture of BST2 and BST2 mutants. CT: cytoplasmic tail. TM: transmembrane domain. EC1: extracellular domain region 1. CC: coiled-coil domain. EC2: extracellular domain region 2. GPI: glycosylphosphatidylinositol anchor. (B-E) The interaction between the Wuhan and Omicron Spikes and different BST2 mutants was investigated by co-IP. GFP was used as a negative control. HIV-1 Vpu was used as a positive control of a membrane virus protein interacting with BST2. Additional controls included beads only (cell lysates of Somicron and full-length BST2) and an IgG control (lysis buffer treated with beads coated with anti-HA antibody). ΔCT: BST2 lacking the cytoplasmic tail. ΔCT+TM: BST2 lacking the cytoplasmic and transmembrane domains. TfR-TM: BST2 harboring the transmembrane domain of the transferrin receptor. ΔCC: BST2 with a truncated coiled-coil domain. ΔEC1: BST2 with deletions in the region between the TM and CC domains. EC2Ala: BST2 containing Ala substitutions in the region between the CC domain and the GPI anchor. Red asterisks indicate bands corresponding to the light chain of the antibody used in the IP. Yellow asterisks indicate bands corresponding to the antibody. Purple asterisks correspond to unspecific bands. Blue pound symbol indicates bands that correspond to the ΔCC mutant. Blots are representative of 3 independent experiments. BST2 diagram was generated in BioRender. (TIFF) [file ppat.1011912.s006.tiff]
