# Supplementary material for: Mutations accumulated in the Spike of SARS-CoV-2 Omicron allow for more efficient counteraction of the restriction factor BST2/Tetherin
Source: PLoS Pathog. 2024 Jan 8;20(1):e1011912. doi: 10.1371/journal.ppat.1011912 (PMC10798645; doi:10.1371/journal.ppat.1011912)
Supplement: S1 Information — (PDF) [file ppat.1011912.s007.pdf]

Fig 1D Spike

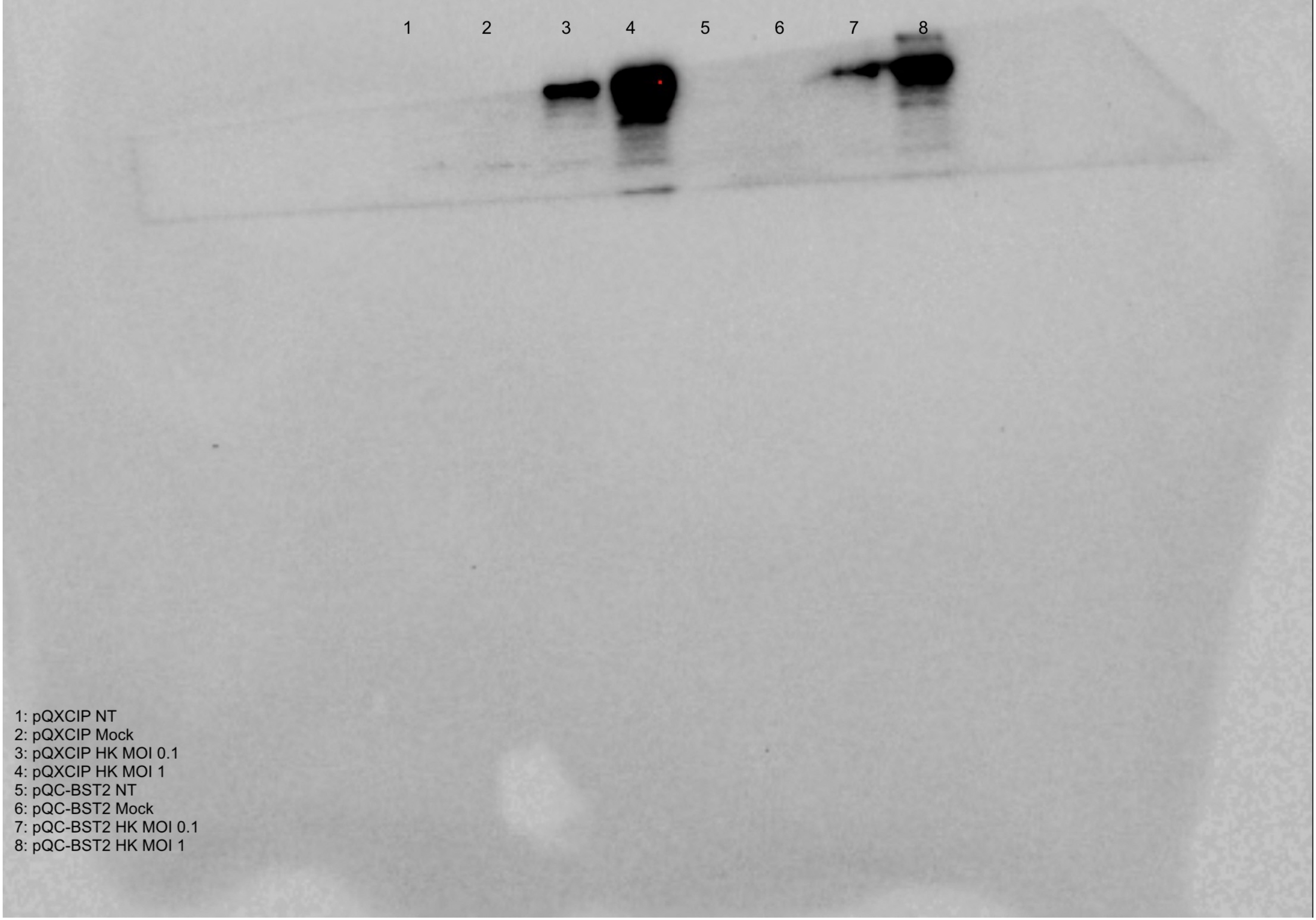

1: pQXCIP NT
2: pQXCIP Mock
3: pQXCIP HK MOI 0.1
4: pQXCIP HK MOI 1
5: pQC-BST2 NT
6: pQC-BST2 Mock
7: pQC-BST2 HK MOI 0.1
8: pQC-BST2 HK MOI 1

Fig 1D N

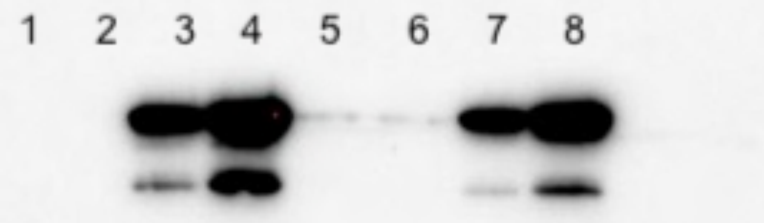

1: pQXCIP NT
2: pQXCIP Mock
3: pQXCIP HK MOI 0.1
4: pQXCIP HK MOI 1
5: pQC-BST2 NT
6: pQC-BST2 Mock
7: pQC-BST2 HK MOI 0.1
8: pQC-BST2 HK MOI 1

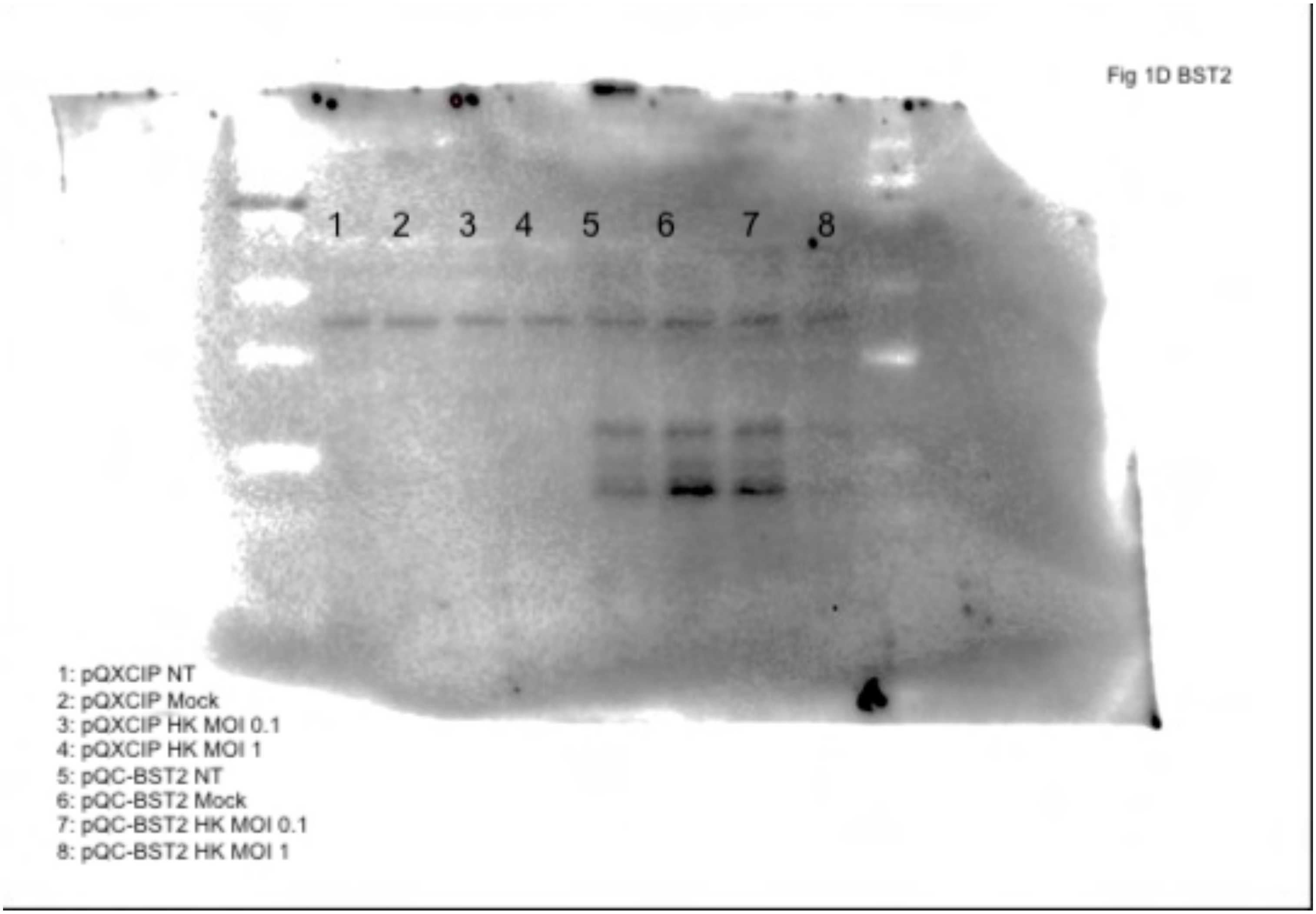
Fig 1D BST2
1
2
3
4
5
6
7
8
1: pQXCIP NT
2: pQXCIP Mock
3: pQXCIP HK MOI 0.1
4: pQXCIP HK MOI 1
5: pQC-BST2 NT
6: pQC-BST2 Mock
7: pQC-BST2 HK MOI 0.1
8: pQC-BST2 HK MOI 1

Fig 1D actin

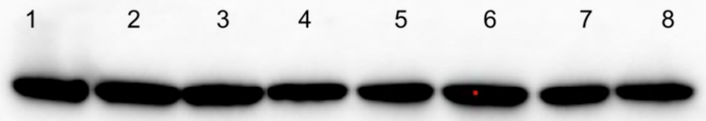

1: pQXCIP NT
2: pQXCIP Mock
3: pQXCIP HK MOI 0.1
4: pQXCIP HK MOI 1
5: pQC-BST2 NT
6: pQC-BST2 Mock
7: pQC-BST2 HK MOI 0.1
8: pQC-BST2 HK MOI 1

2A, Spike

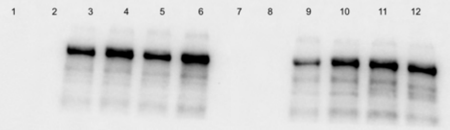

1: pQXCIP NT
2: pQXCIP Mock
3: pQXCIP HK MOI 0.1
4: pQXCIP HK MOI 1
5: pQXCIP Omicron MOI 0.1
6: pQXCIP Omicron MOI 1
7: pQC-BST2 NT
8: pQC-BST2 Mock
9: pQC-BST2 HK MOI 0.1
10: pQC-BST2 HK MOI 1
11: pQC-BST2 Omicron MOI 0.1
12: pQC-BST2 Omicron MOI 1

2A, N

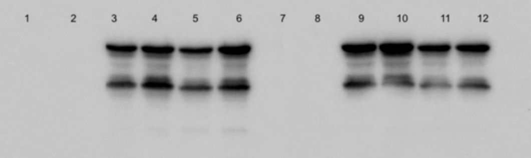

1: pQXCIP NT
2: pQXCIP Mock
3: pQXCIP HK MOI 0.1
4: pQXCIP HK MOI 1
5: pQXCIP Omicron MOI 0.1
6: pQXCIP Omicron MOI 1
7: pQC-BST2 NT
8: pQC-BST2 Mock
9: pQC-BST2 HK MOI 0.1
10: pQC-BST2 HK MOI 1
11: pQC-BST2 Omicron MOI 0.1
12: pQC-BST2 Omicron MOI 1

2A, BST2

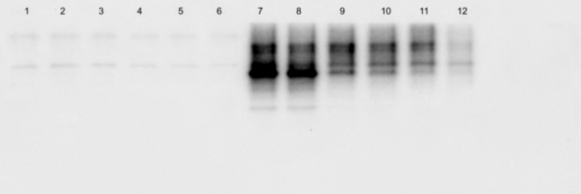

1: pQXCIP NT
2: pQXCIP Mock
3: pQXCIP HK MOI 0.1
4: pQXCIP HK MOI 1
5: pQXCIP Omicron MOI 0.1
6: pQXCIP Omicron MOI 1
7: pQC-BST2 NT
8: pQC-BST2 Mock
9: pQC-BST2 HK MOI 0.1
10: pQC-BST2 HK MOI 1
11: pQC-BST2 Omicron MOI 0.1
12: pQC-BST2 Omicron MOI 1

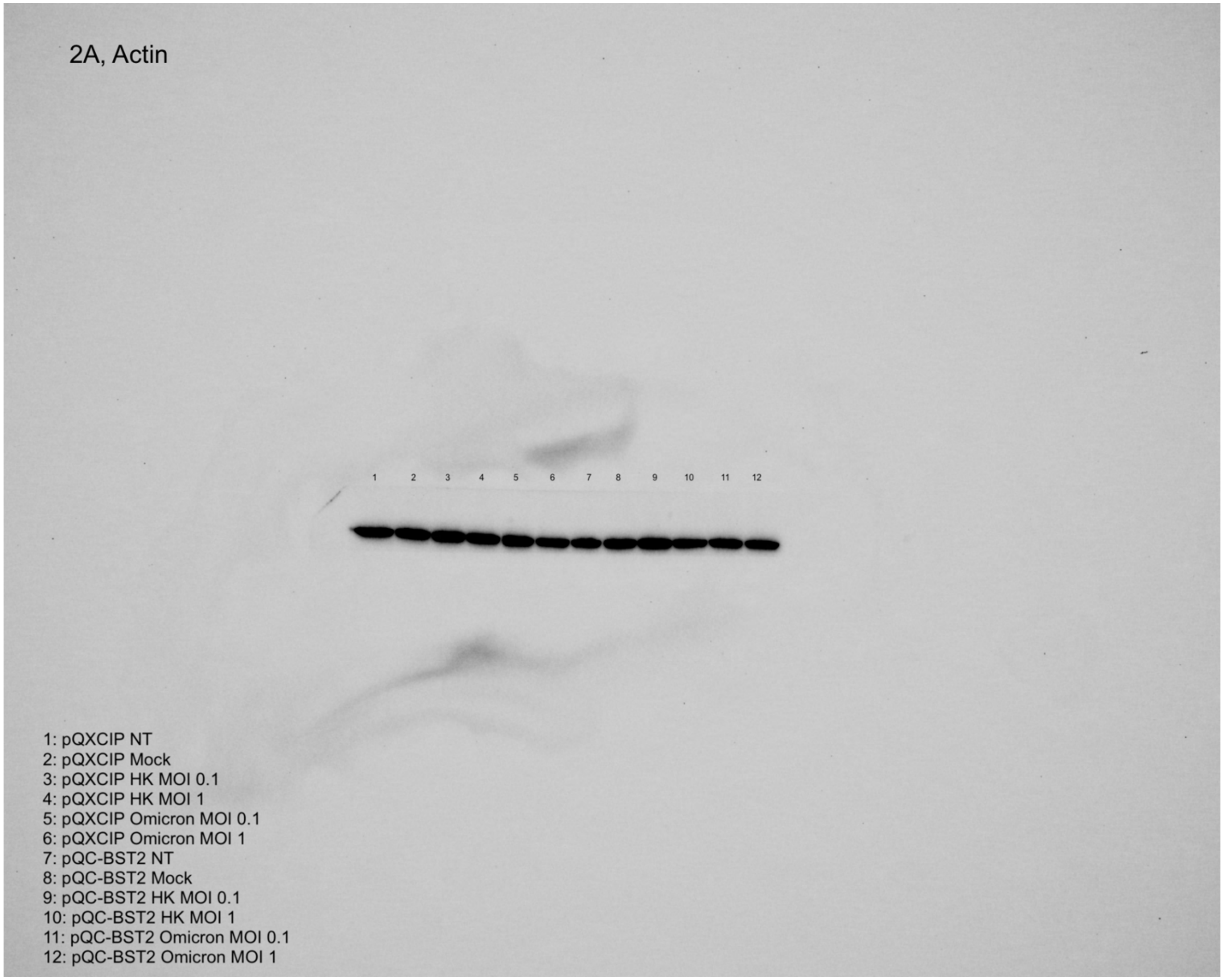
2A, Actin
1
2
3
4
5
6
7
8
9
10
11
12
1: pQXCIP NT
2: pQXCIP Mock
3: pQXCIP HK MOI 0.1
4: pQXCIP HK MOI 1
5: pQXCIP Omicron MOI 0.1
6: pQXCIP Omicron MOI 1
7: pQC-BST2 NT
8: pQC-BST2 Mock
9: pQC-BST2 HK MOI 0.1
10: pQC-BST2 HK MOI 1
11: pQC-BST2 Omicron MOI 0.1
12: pQC-BST2 Omicron MOI 1

2C, BST2

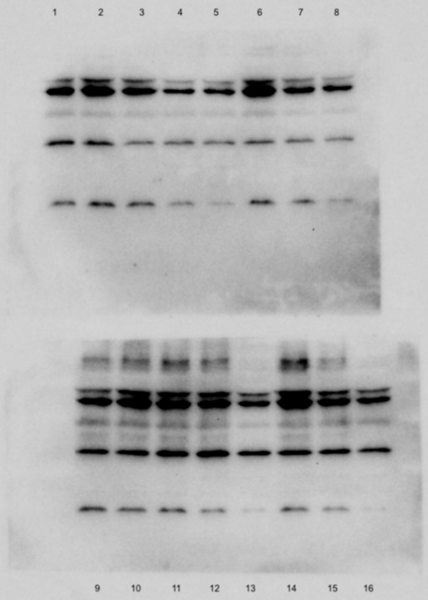

1: NT
2: VLP
3: A549 HK MOI 0.1
4: A549 HK MOI 1
5: A549 HK MOI 5
6: A549 Omi MOI 0.1
7: A549 Omi MOI 1
8: A549 Omi MOI 5

9: NT+ IFN
10: VLP+ IFN
11: A549 + IFN HK MOI 0.1
12: A549 + IFN HK MOI 1
13: A549 + IFN HK MOI 5
14: A549 + IFN Omi MOI 0.1
15: A549 + IFN Omi MOI 1
16: A549 + IFN Omi MOI 5

2C, Spike

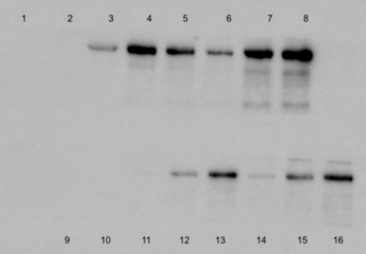

1: NT
2: VLP
3: A549 HK MOI 0.1
4: A549 HK MOI 1
5: A549 HK MOI 5
6: A549 Omi MOI 0.1
7: A549 Omi MOI 1
8: A549 Omi MOI 5

9: NT+ IFN
10: VLP+ IFN
11: A549 + IFN HK MOI 0.1
12: A549 + IFN HK MOI 1
13: A549 + IFN HK MOI 5
14: A549 + IFN Omi MOI 0.1
15: A549 + IFN Omi MOI 1
16: A549 + IFN Omi MOI 5

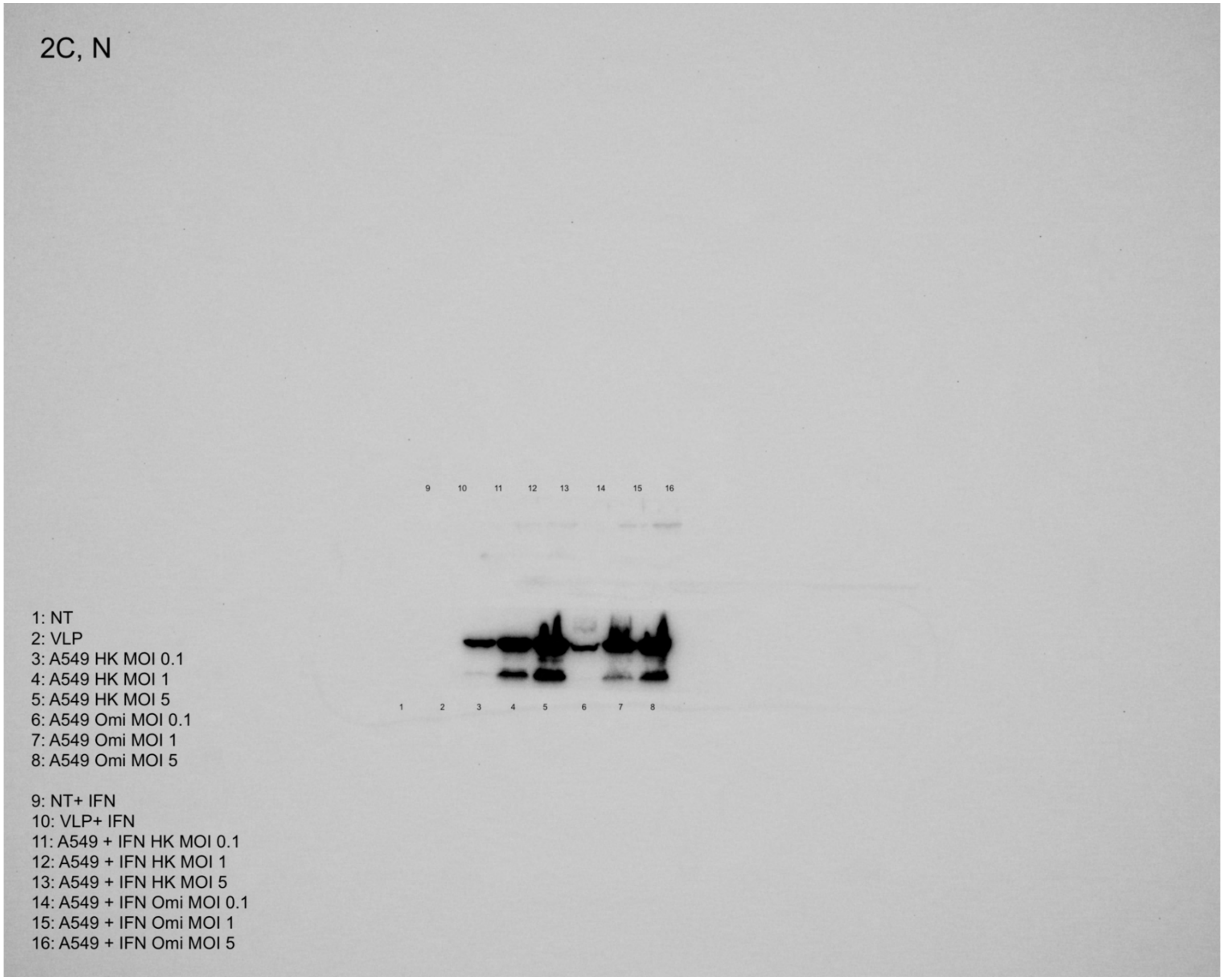
2C, N
9 10 11 12 13 14 15 16
1 2 3 4 5 6 7 8
1: NT
2: VLP
3: A549 HK MOI 0.1
4: A549 HK MOI 1
5: A549 HK MOI 5
6: A549 Omi MOI 0.1
7: A549 Omi MOI 1
8: A549 Omi MOI 5
9: NT+ IFN
10: VLP+ IFN
11: A549 + IFN HK MOI 0.1
12: A549 + IFN HK MOI 1
13: A549 + IFN HK MOI 5
14: A549 + IFN Omi MOI 0.1
15: A549 + IFN Omi MOI 1
16: A549 + IFN Omi MOI 5

2C, Actin

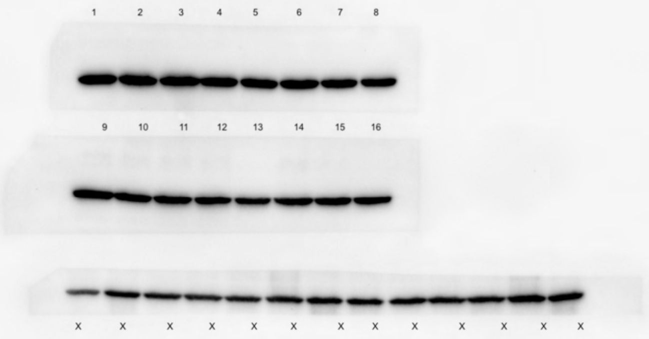

1: NT
2: VLP
3: A549 HK MOI 0.1
4: A549 HK MOI 1
5: A549 HK MOI 5
6: A549 Omi MOI 0.1
7: A549 Omi MOI 1
8: A549 Omi MOI 5

9: NT+ IFN
10: VLP+ IFN
11: A549 + IFN HK MOI 0.1
12: A549 + IFN HK MOI 1
13: A549 + IFN HK MOI 5
14: A549 + IFN Omi MOI 0.1
15: A549 + IFN Omi MOI 1
16: A549 + IFN Omi MOI 5

2C, N, higher exposure

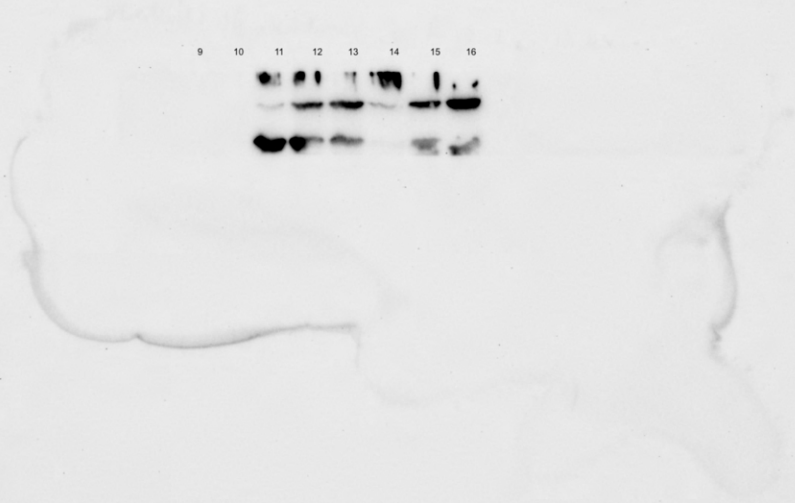

9: NT+ IFN
10: VLP+ IFN
11: A549 + IFN HK MOI 0.1
12: A549 + IFN HK MOI 1
13: A549 + IFN HK MOI 5
14: A549 + IFN Omi MOI 0.1
15: A549 + IFN Omi MOI 1
16: A549 + IFN Omi MOI 5

3A left, Spike

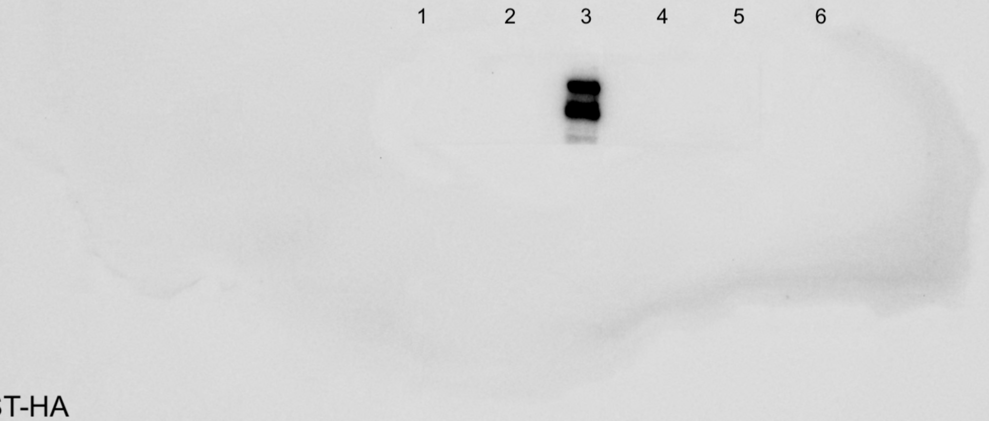

1: GST-HA
2: Vpu
3: SARS-CoV-2 Spike
4: SARS-CoV-2 NSP1
5: SARS-CoV-2 NSP8
6: SARS-CoV-2 NSP9

3A left, BST2

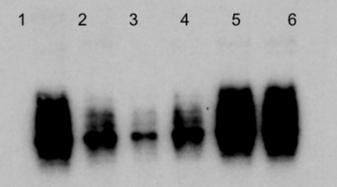

1: GST-HA
2: Vpu
3: SARS-CoV-2 Spike
4: SARS-CoV-2 NSP1
5: SARS-CoV-2 NSP8
6: SARS-CoV-2 NSP9

3A left, HA + Strep-Tag

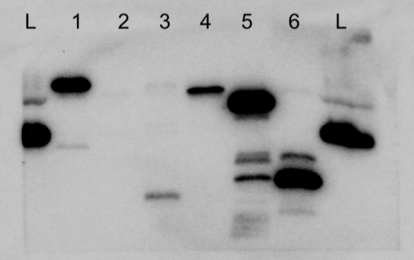

L: ladder
1: GST-HA
2: Vpu
3: SARS-CoV-2 Spike
4: SARS-CoV-2 NSP1
5: SARS-CoV-2 NSP8
6: SARS-CoV-2 NSP9

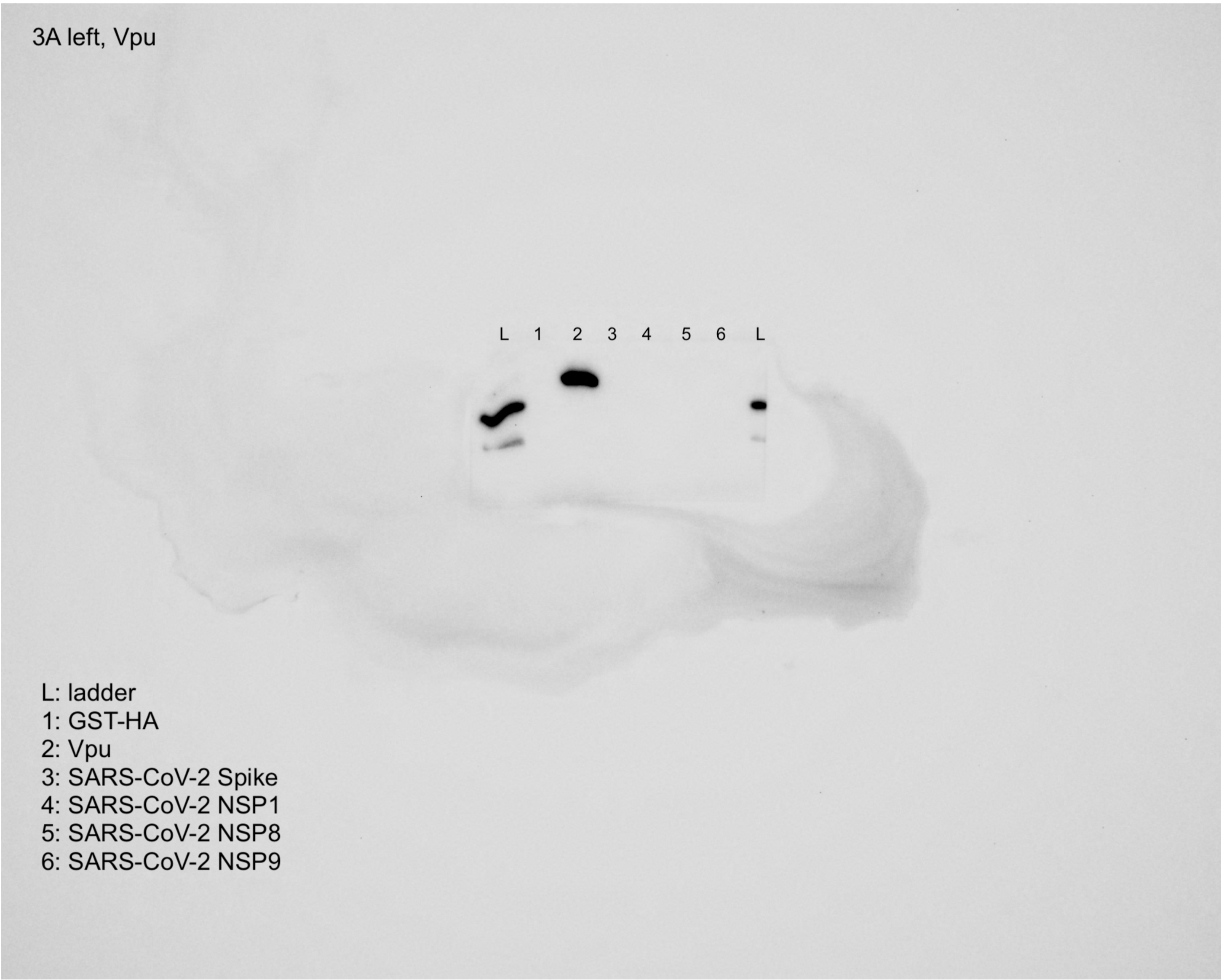

L: ladder
1: GST-HA
2: Vpu
3: SARS-CoV-2 Spike
4: SARS-CoV-2 NSP1
5: SARS-CoV-2 NSP8
6: SARS-CoV-2 NSP9

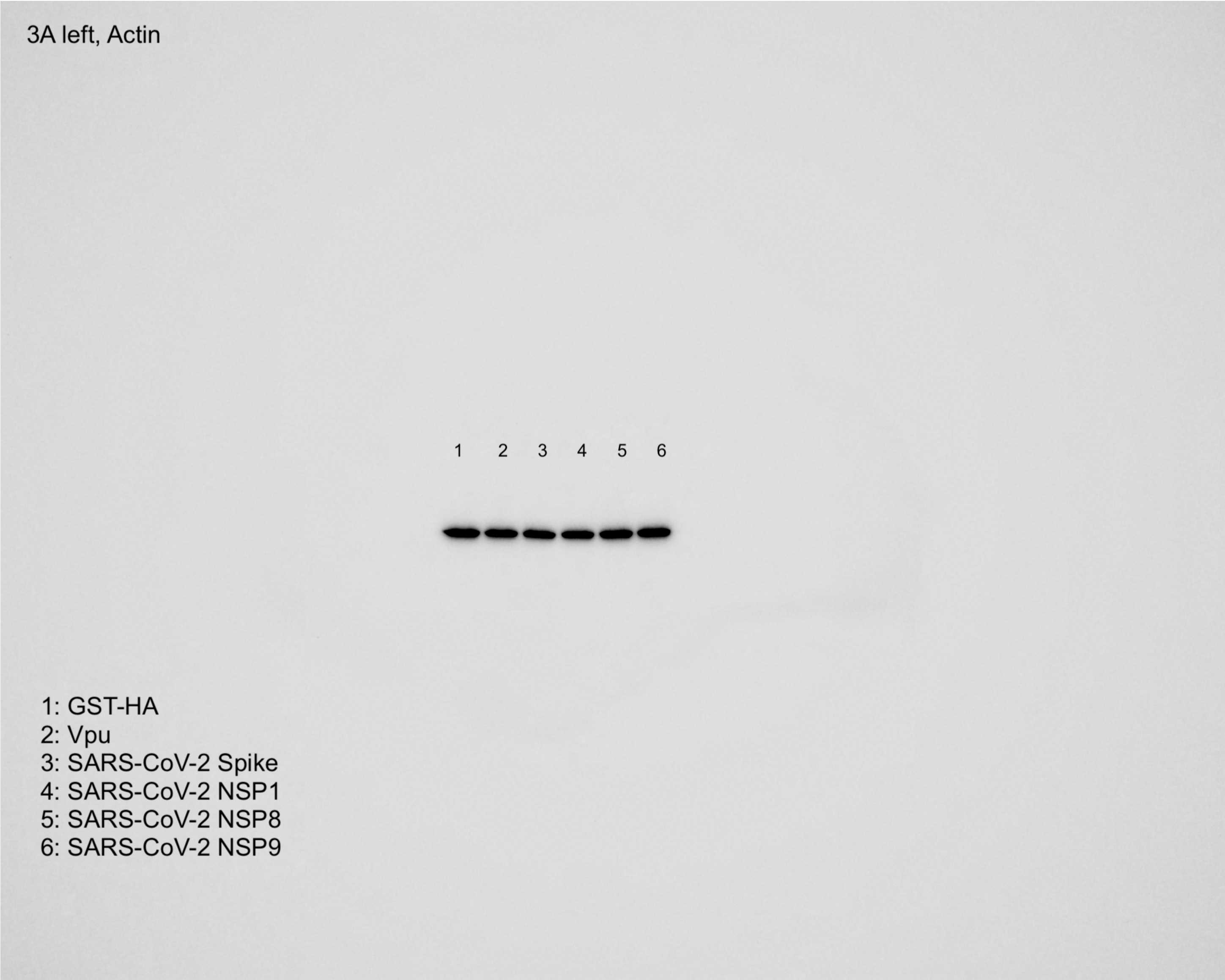
3A left, Actin
1 2 3 4 5 6
1: GST-HA
2: Vpu
3: SARS-CoV-2 Spike
4: SARS-CoV-2 NSP1
5: SARS-CoV-2 NSP8
6: SARS-CoV-2 NSP9

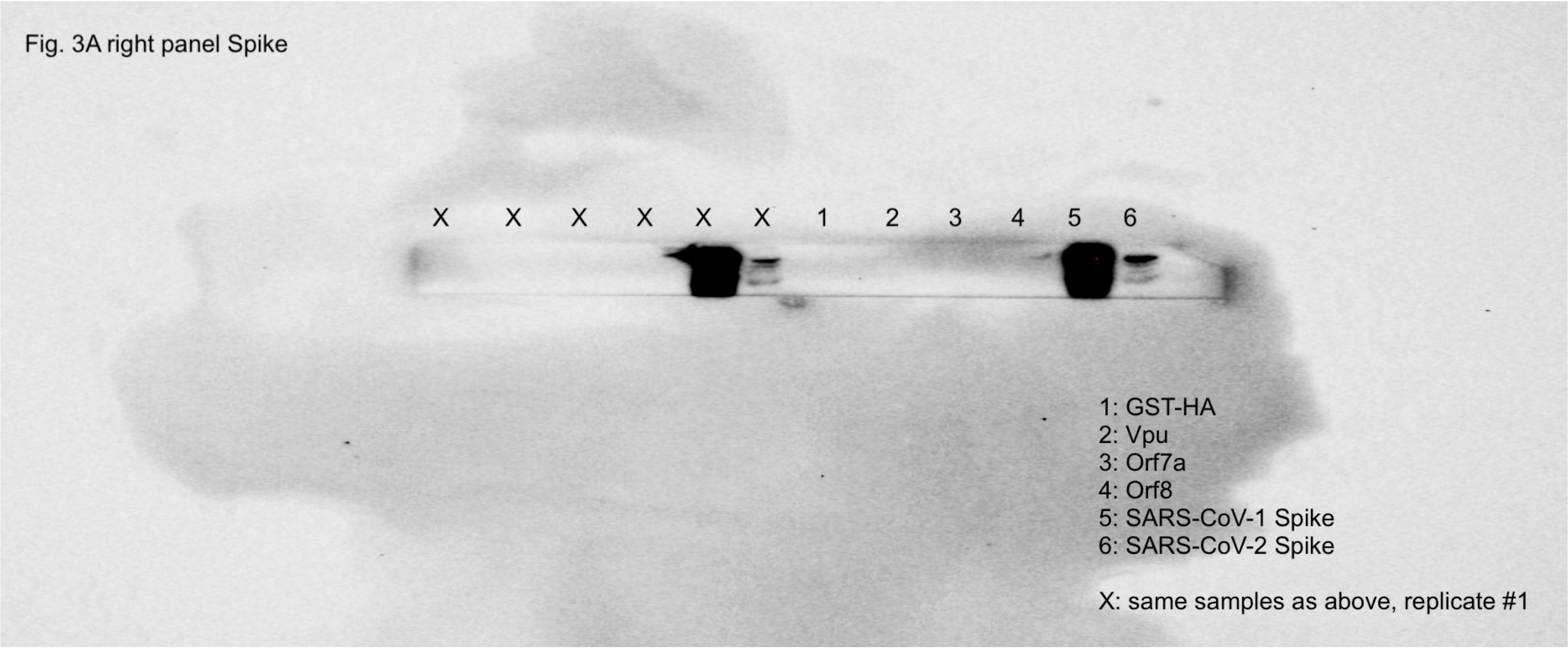
Fig. 3A right panel Spike
X X X X X X 1 2 3 4 5 6
1: GST-HA
2: Vpu
3: Orf7a
4: Orf8
5: SARS-CoV-1 Spike
6: SARS-CoV-2 Spike
X: same samples as above, replicate #1

Fig 3A, right panel BST2

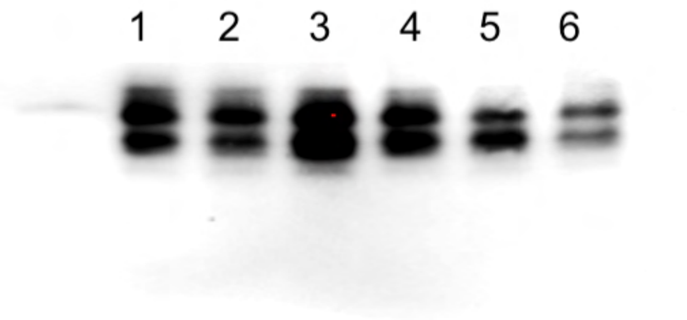

1: GST-HA
2: Vpu
3: Orf7a
4: Orf8
5: SARS-CoV-1 Spike
6: SARS-CoV-2 Spike

3A, right panel Strep tag

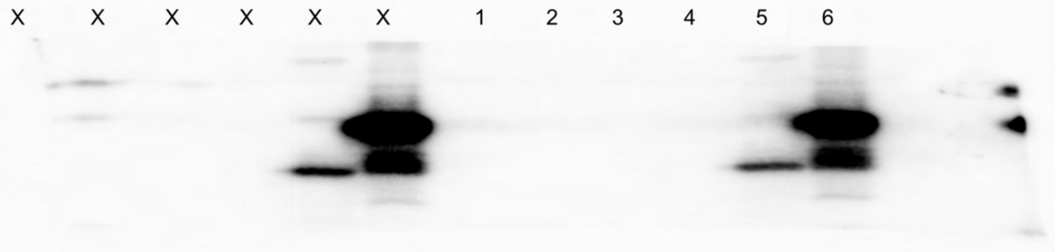

1: GST-HA
2: Vpu
3: Orf7a
4: Orf8
5: SARS-CoV-1 Spike
6: SARS-CoV-2 Spike

X: same samples as above, replicate #1

3A, right panel GST-HA

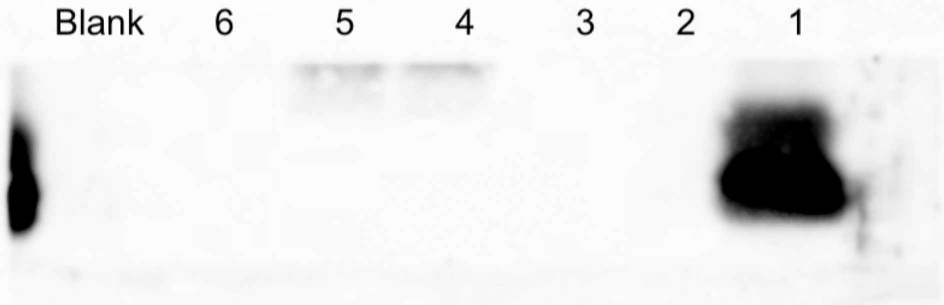

1: GST-HA
2: Vpu
3: Orf7a
4: Orf8
5: SARS-CoV-1 Spike
6: SARS-CoV-2 Spike

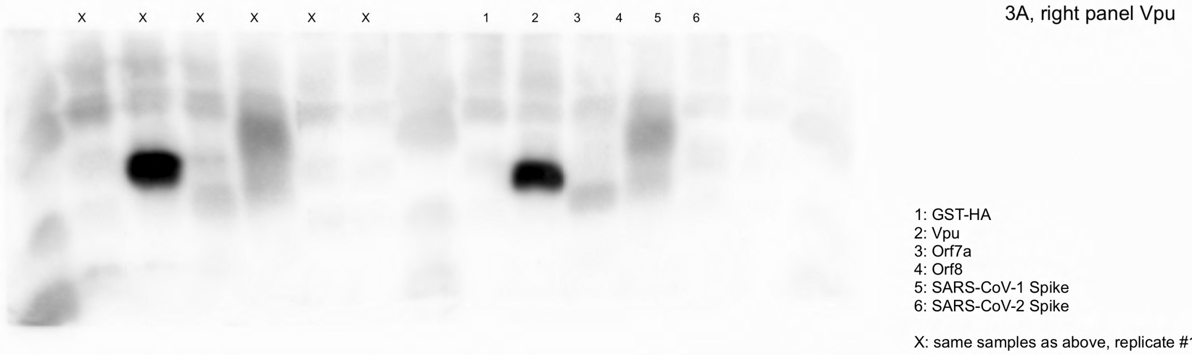

1: GST-HA
2: Vpu
3: Orf7a
4: Orf8
5: SARS-CoV-1 Spike
6: SARS-CoV-2 Spike

X: same samples as above, replicate #1

3A, right panel Actin

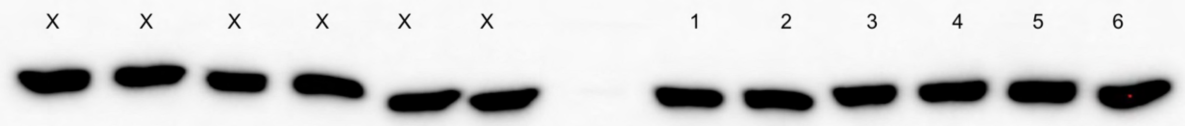

1: GST-HA
2: Vpu
3: Orf7a
4: Orf8
5: SARS-CoV-1 Spike
6: SARS-CoV-2 Spike

X: same samples as above, replicate #1

3B, Spike

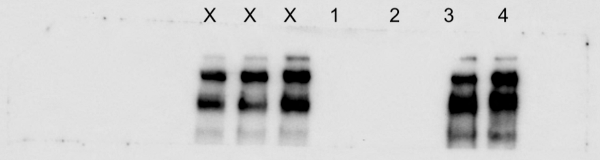

X
X
X
1: GST-HA
2: Vpu
3: Wuhan Spike
4: Omicron Spike

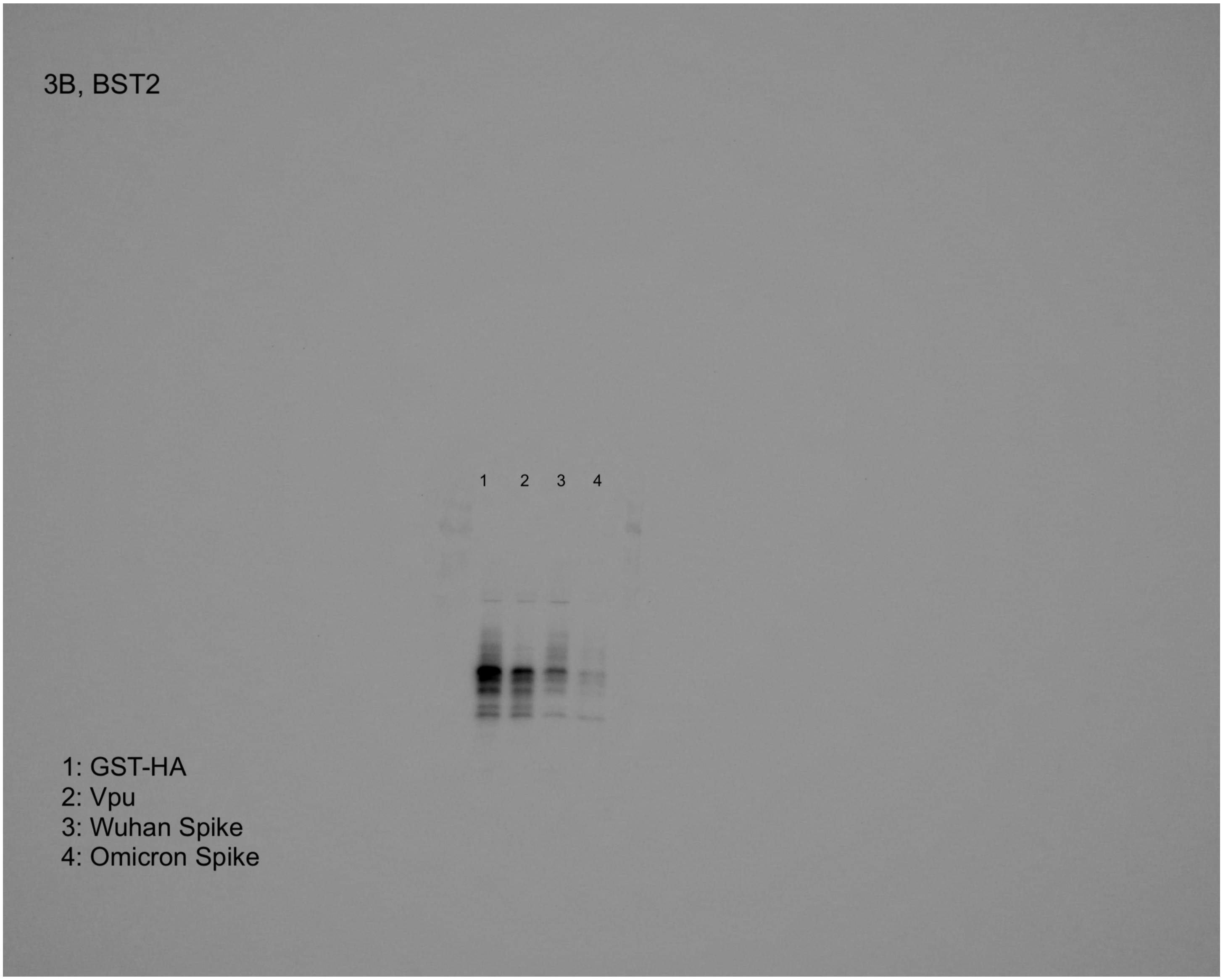
3B, BST2
1 2 3 4
1: GST-HA
2: Vpu
3: Wuhan Spike
4: Omicron Spike

3B, HA-GST

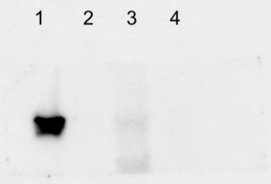

1: GST-HA
2: Vpu
3: Wuhan Spike
4: Omicron Spike

3B, Vpu

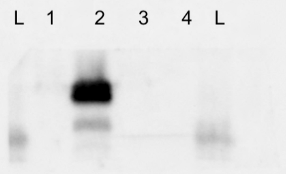

L: ladder
1: GST-HA
2: Vpu
3: Wuhan Spike
4: Omicron Spike

3B, Actin

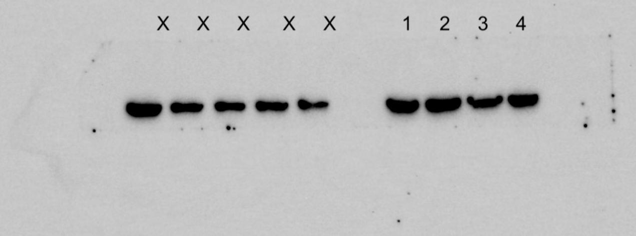

X
X
X
X
X
1: GST-HA
2: Vpu
3: Wuhan Spike
4: Omicron Spike

4A Spike-BST2 IP, Spike

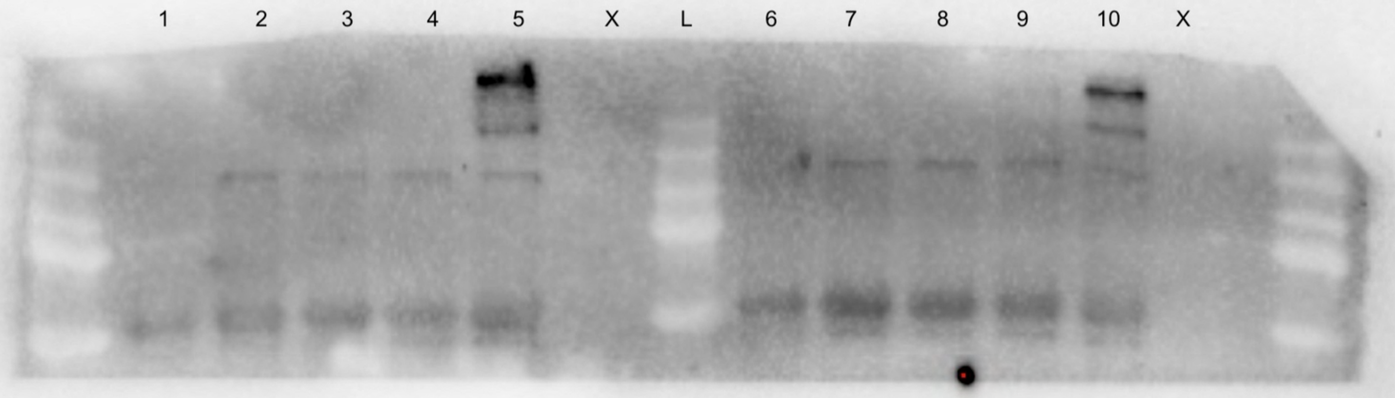

1: Beads
2: pcDNA3 – vector
3: pcDNA3 – SARS-CoV-1 Spike
4: BST2 – vector
5: BST2 – SARS-CoV-1 Spike
X: BST2 – vector input

L: ladder

6: Beads
7: pcDNA3 – vector
8: pcDNA3 – SARS-CoV-2 Spike
9: BST2 – vector
10: BST2 – SARS-CoV-2 Spike
X: BST2 – vector input

4A, BST2 IP

1: Beads
2: pcDNA3 – vector
3: pcDNA3 – SARS-CoV-1 Spike
4: BST2 – vector
5: BST2 – SARS-CoV-1 Spike
X: BST2 – vector input

6: Beads
7: pcDNA3 – vector
8: pcDNA3 – SARS-CoV-2 Spike
9: BST2 – vector
10: BST2 – SARS-CoV-2 Spike
X: BST2 – vector input

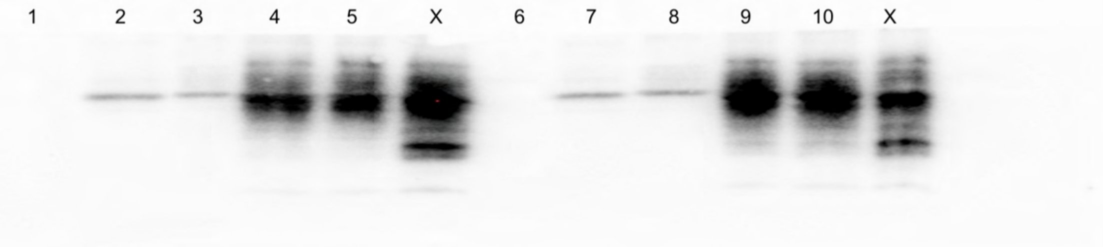

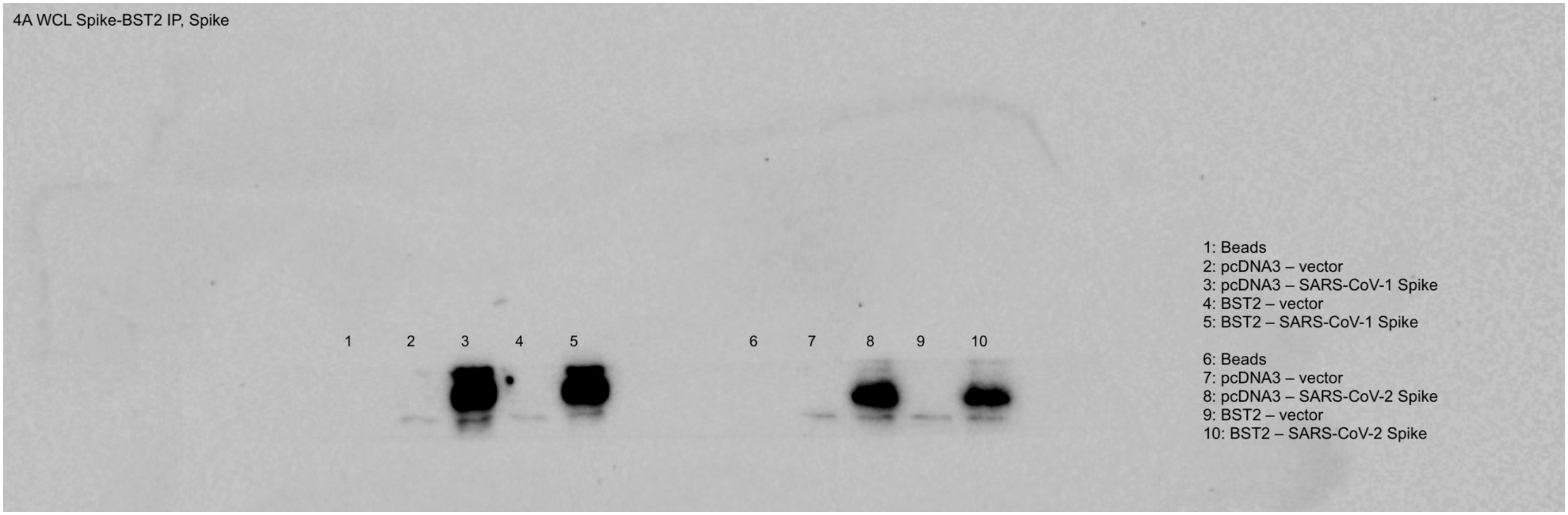
4A WCL Spike-BST2 IP, Spike
1
2
3
4
5
6
7
8
9
10
1: Beads
2: pcDNA3 – vector
3: pcDNA3 – SARS-CoV-1 Spike
4: BST2 – vector
5: BST2 – SARS-CoV-1 Spike
6: Beads
7: pcDNA3 – vector
8: pcDNA3 – SARS-CoV-2 Spike
9: BST2 – vector
10: BST2 – SARS-CoV-2 Spike

4A, left panel WCL BST2

1: Beads
2: pcDNA3 – vector
3: pcDNA3 – SARS-CoV-1 Spike
4: BST2 – vector
5: BST2 – SARS-CoV-1 Spike
X: BST2 – SARS-CoV-1 Spike tech repl.

L: ladder4

6: Beads
7: pcDNA3 – vector
8: pcDNA3 – SARS-CoV-2 Spike
9: BST2 – vector
10: BST2 – SARS-CoV-2 Spike
X: BST2 – SARS-CoV-2 Spike tech repl.

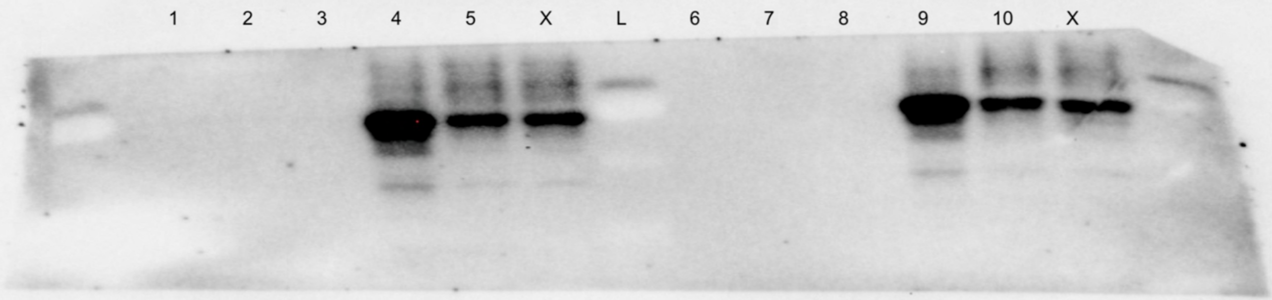

4A WCL Spike-BST2 IP,

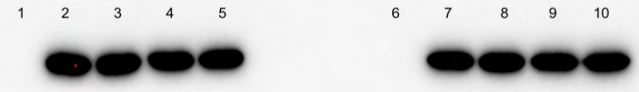

1: Beads
2: pcDNA3 – vector
3: pcDNA3 – SARS-CoV-1 Spike
4: BST2 – vector
5: BST2 – SARS-CoV-1 Spike

6: Beads
7: pcDNA3 – vector
8: pcDNA3 – SARS-CoV-2 Spike
9: BST2 – vector
10: BST2 – SARS-CoV-2 Spike

4A Orf7a-Spike IP, Orf7a

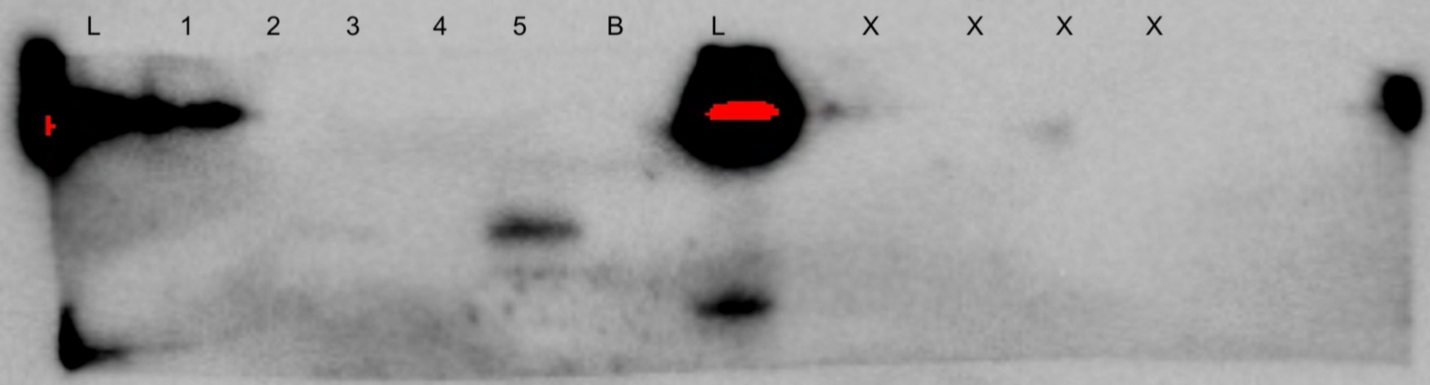

Ladder
1: Beads
2: pcDNA3 – vector
3: pcDNA3 – SARS-CoV-2 Orf7a
4: BST2 – vector
5: BST2 – SARS-CoV-2 Orf7a
B: Blank
L: Ladder
X: pcDNA3 – vector
X: pcDNA3 – SARS-CoV-2 Orf8
X: BST2 – vector
X: BST2 – SARS-CoV-2 Orf8

1: Beads
2: pcDNA3 – vector
3: pcDNA3 – SARS-CoV-2 Orf7a
4: BST2 – vector
5: BST2 – SARS-CoV-2 Orf7a
X: pcDNA3 – vector
X: pcDNA3 – SARS-CoV-2 Orf8
X: BST2 – vector
X: BST2 – SARS-CoV-2 Orf8

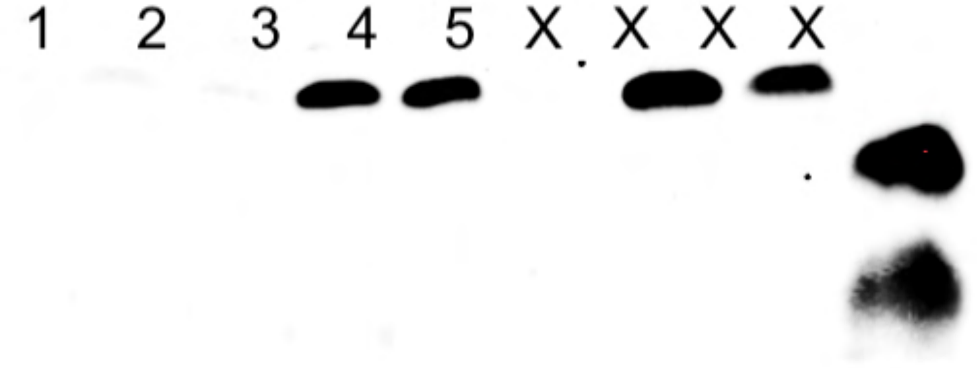

4A Orf7-BST2 IP, BST2

4A Orf7a WCL strep

L: ladder
1: Beads
2: pcDNA3 – vector
3: pcDNA3 – SARS-CoV-2 Orf7a
4: BST2 – vector
5: BST2 – SARS-CoV-2 Orf7a
B: Blank
L: Ladder
X: beads
X: pcDNA3 – vector
X: pcDNA3 – SARS-CoV-2 Orf8
X: BST2 – vector
X: BST2 – SARS-CoV-2 Orf8
B: blank
L: ladder

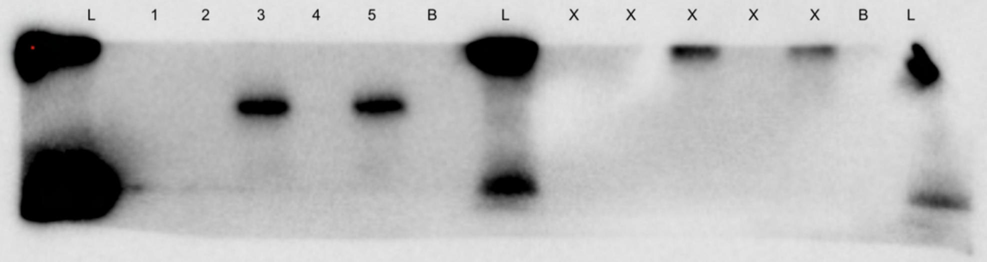

4A right panel, WCL BST2

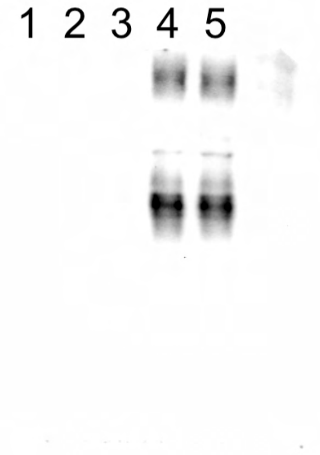

1: Beads
2: pcDNA3 – vector
3: pcDNA3 – SARS-CoV-2 Orf7a
4: BST2 – vector
5: BST2 – SARS-CoV-2 Orf7a

4A, right panel WCL actin

Ladder
1: Beads
2: pcDNA3 – vector
3: pcDNA3 – SARS-CoV-2 Orf7a
4: BST2 – vector
5: BST2 – SARS-CoV-2 Orf7a

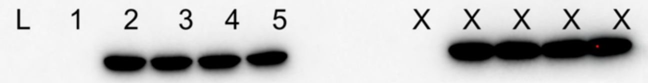

X: pcDNA3 – vector
X: pcDNA3 – SARS-CoV-2 Orf8
X: BST2 – vector
X: BST2 – SARS-CoV-2 Orf8

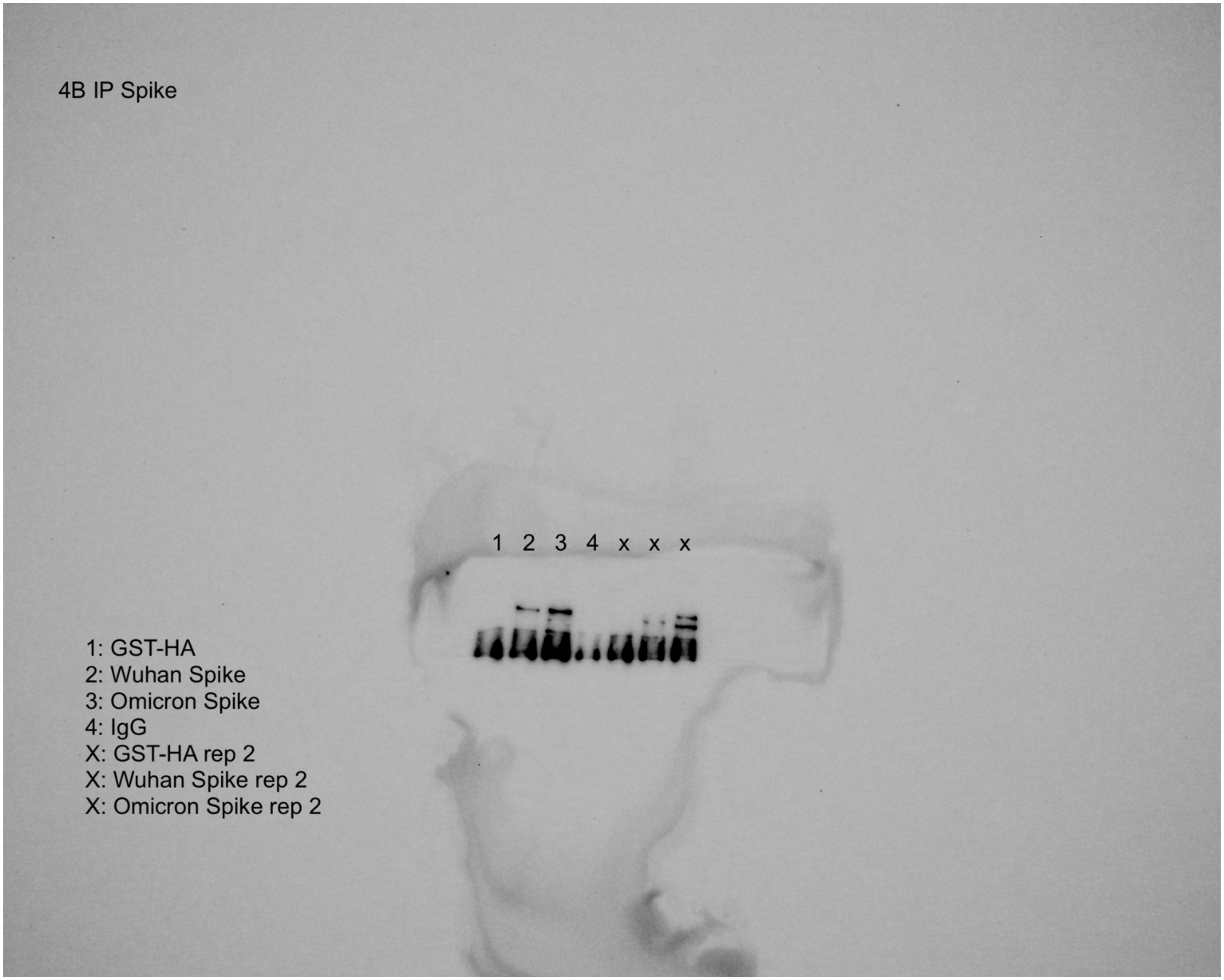
4B IP Spike
1 2 3 4 x x x
1: GST-HA
2: Wuhan Spike
3: Omicron Spike
4: IgG
X: GST-HA rep 2
X: Wuhan Spike rep 2
X: Omicron Spike rep 2

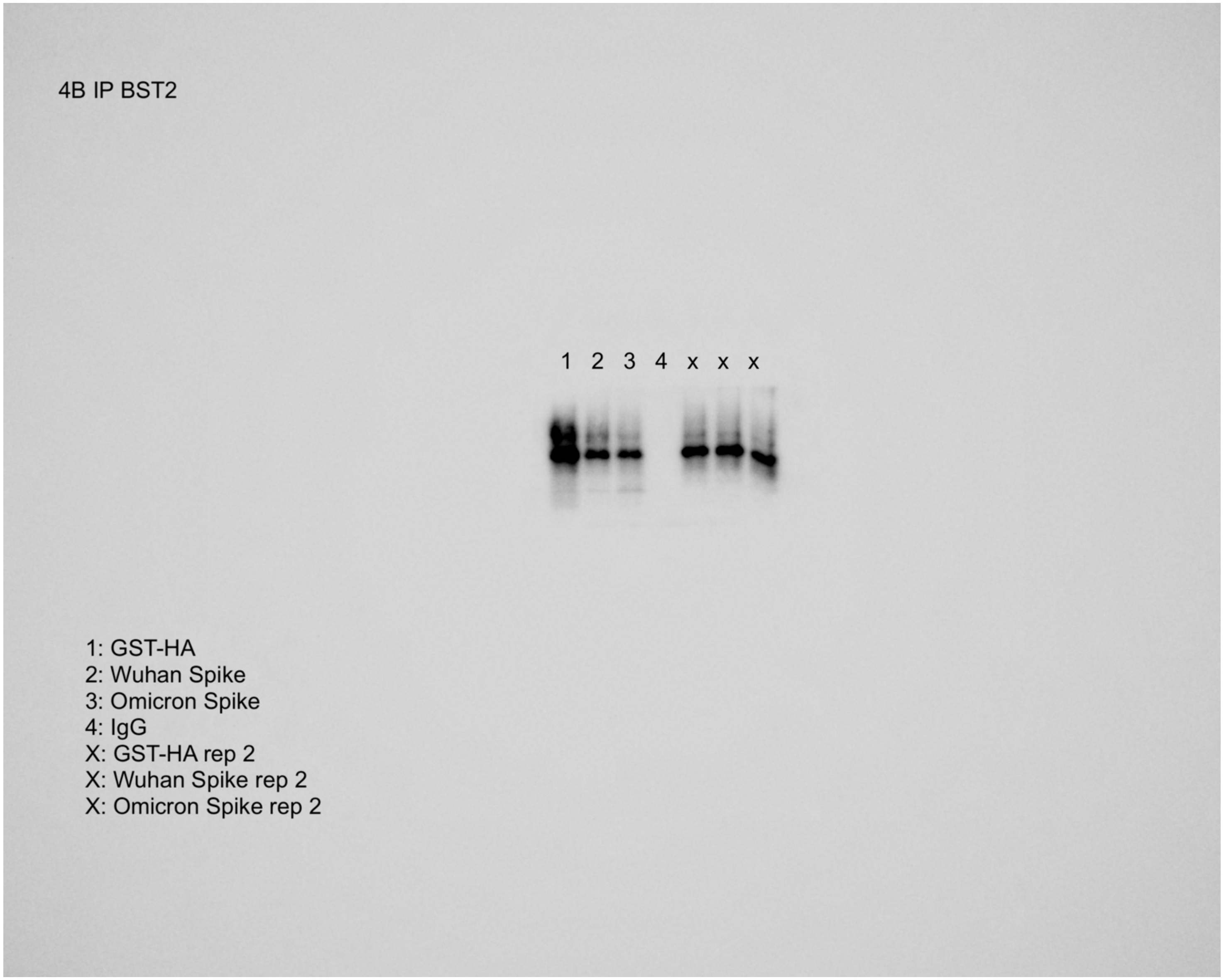
4B IP BST2
1 2 3 4 x x x
1: GST-HA
2: Wuhan Spike
3: Omicron Spike
4: IgG
X: GST-HA rep 2
X: Wuhan Spike rep 2
X: Omicron Spike rep 2

4B WCL Spike

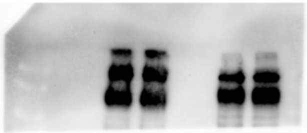

1: GST-HA
2: Wuhan Spike
3: Omicron Spike
4: IgG
X: GST-HA rep 2
X: Wuhan Spike rep 2
X: Omicron Spike rep 2

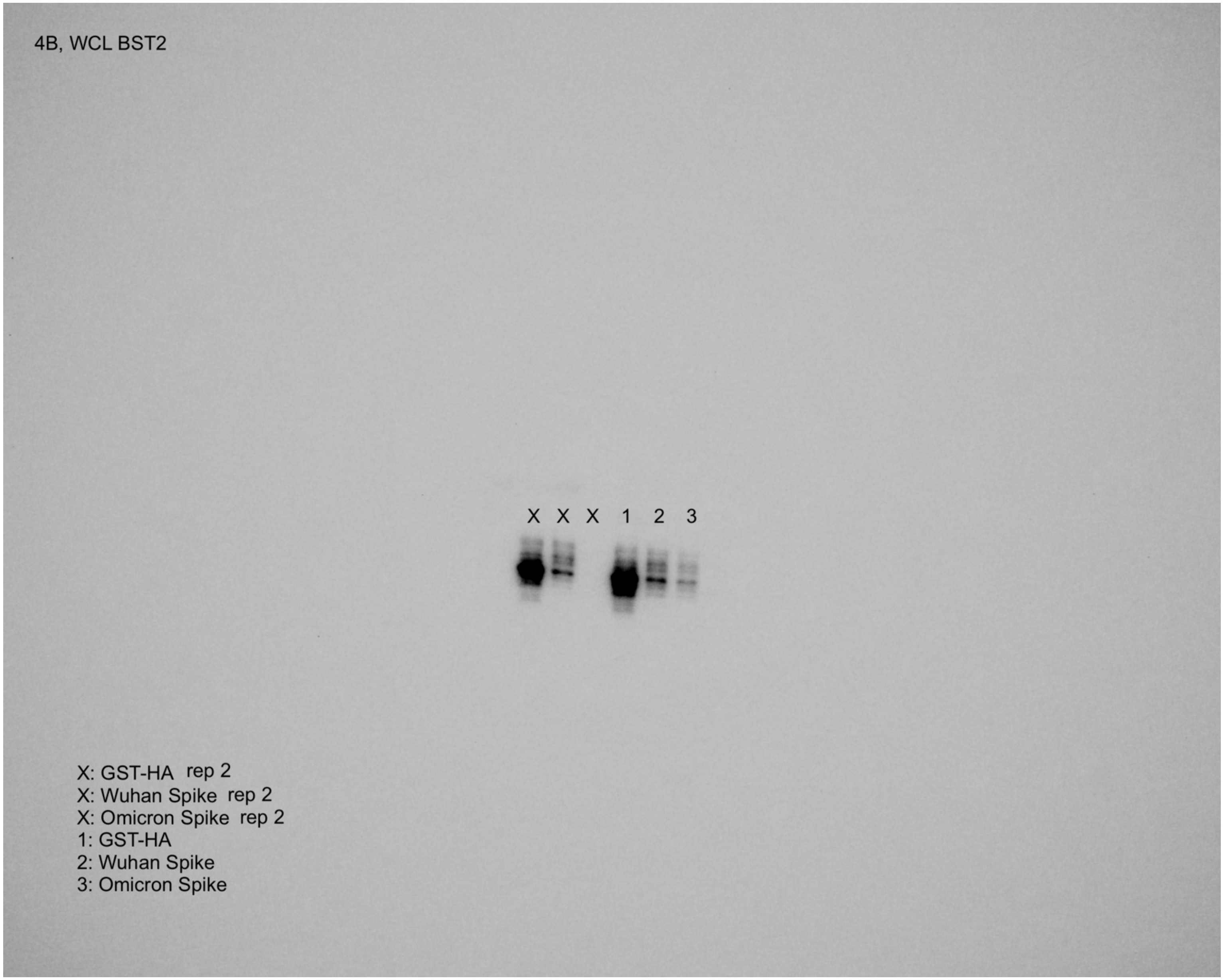
4B, WCL BST2
X X X 1 2 3
X: GST-HA rep 2
X: Wuhan Spike rep 2
X: Omicron Spike rep 2
1: GST-HA
2: Wuhan Spike
3: Omicron Spike

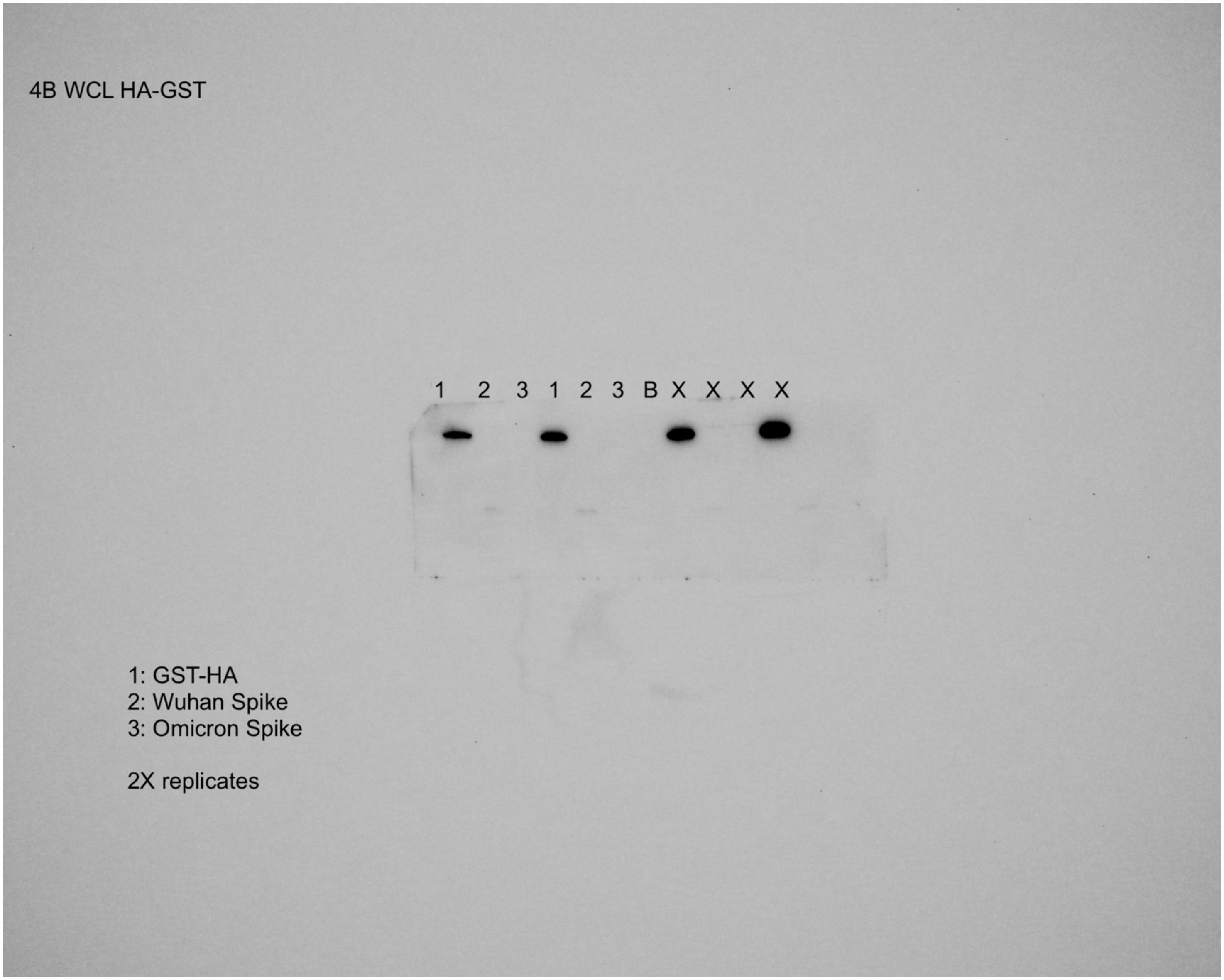
4B WCL HA-GST
1 2 3 1 2 3 B X X X X
1: GST-HA
2: Wuhan Spike
3: Omicron Spike
2X replicates

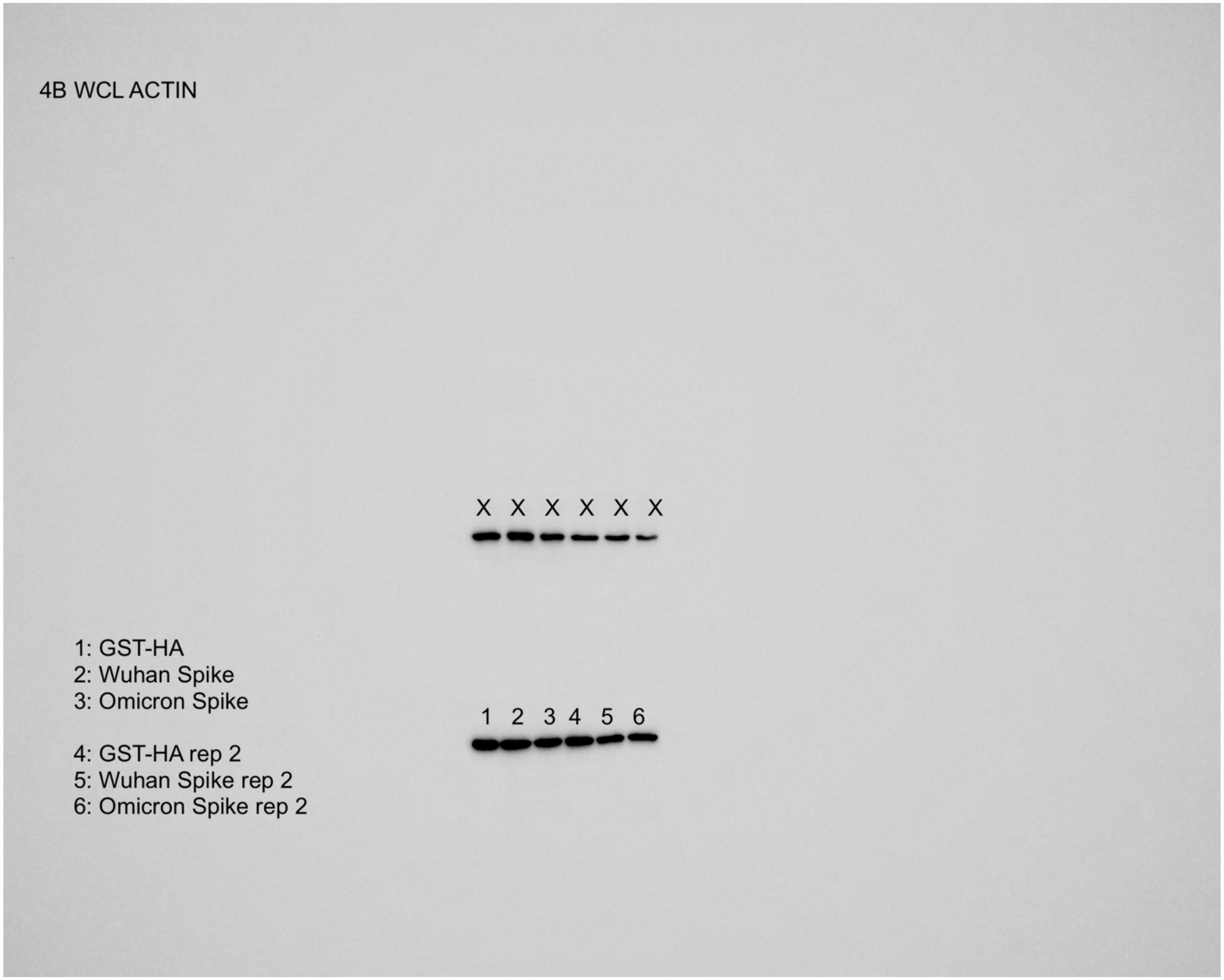
4B WCL ACTIN
X X X X X X
1 2 3 4 5 6
1: GST-HA
2: Wuhan Spike
3: Omicron Spike
4: GST-HA rep 2
5: Wuhan Spike rep 2
6: Omicron Spike rep 2

5B IP Spike

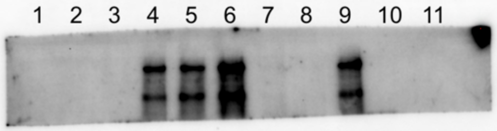

1: GFP + BST2
2: VPU + BST2
3: Vpu + TfR-TM
4: Swu + BST2
5: Swu + deltaCT
6: Swu + TfR-TM
7: Swu + deltaEC1
8: Swu + deltaCC
9: Swu + EC2Ala
10: Beads
11: IgG

5B IP BST2

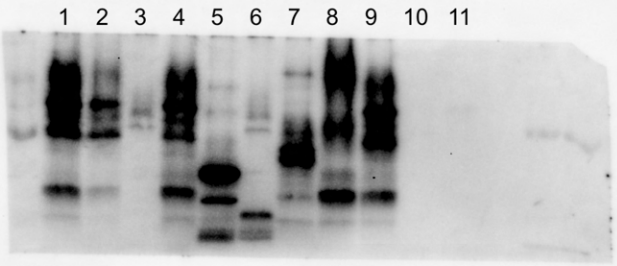

1: GFP + BST2
2: VPU + BST2
3: Vpu + TfR-TM
4: Swu + BST2
5: Swu + deltaCT
6: Swu + TfR-TM
7: Swu + deltaEC1
8: Swu + deltaCC
9: Swu + EC2Ala
10: Beads
11: IgG

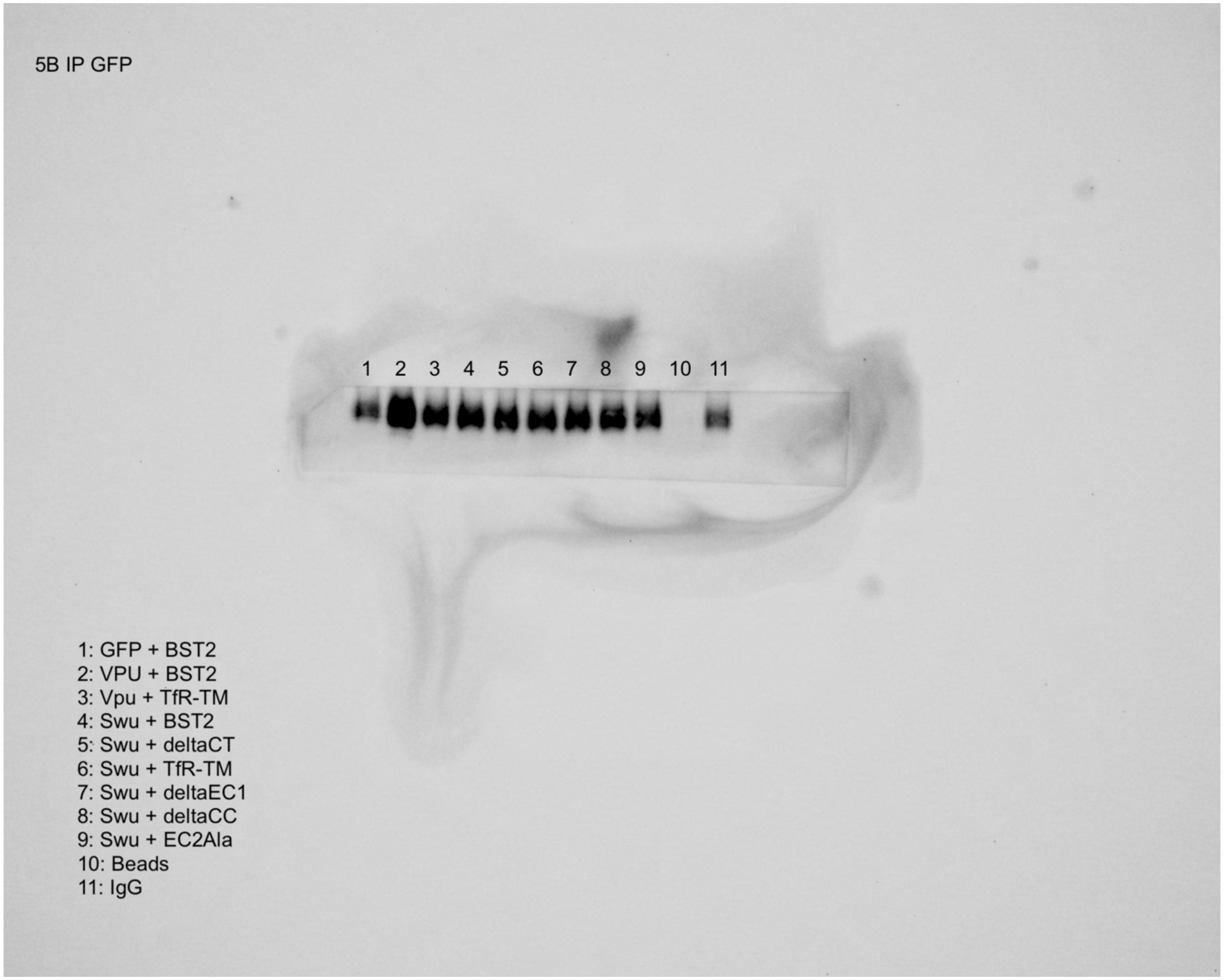
5B IP GFP
1 2 3 4 5 6 7 8 9 10 11
1: GFP + BST2
2: VPU + BST2
3: Vpu + TfR-TM
4: Swu + BST2
5: Swu + deltaCT
6: Swu + TfR-TM
7: Swu + deltaEC1
8: Swu + deltaCC
9: Swu + EC2Ala
10: Beads
11: IgG

5B IP Vpu

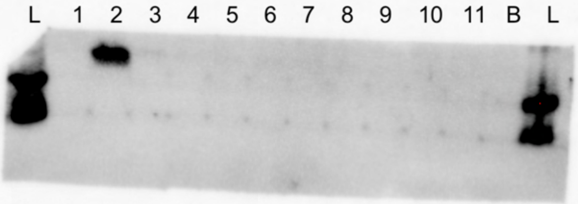

L: ladder
1: GFP + BST2
2: VPU + BST2
3: Vpu + TfR-TM
4: Swu + BST2
5: Swu + deltaCT
6: Swu + TfR-TM
7: Swu + deltaEC1
8: Swu + deltaCC
9: Swu + EC2Ala
10: Beads
11: IgG
B: Blank

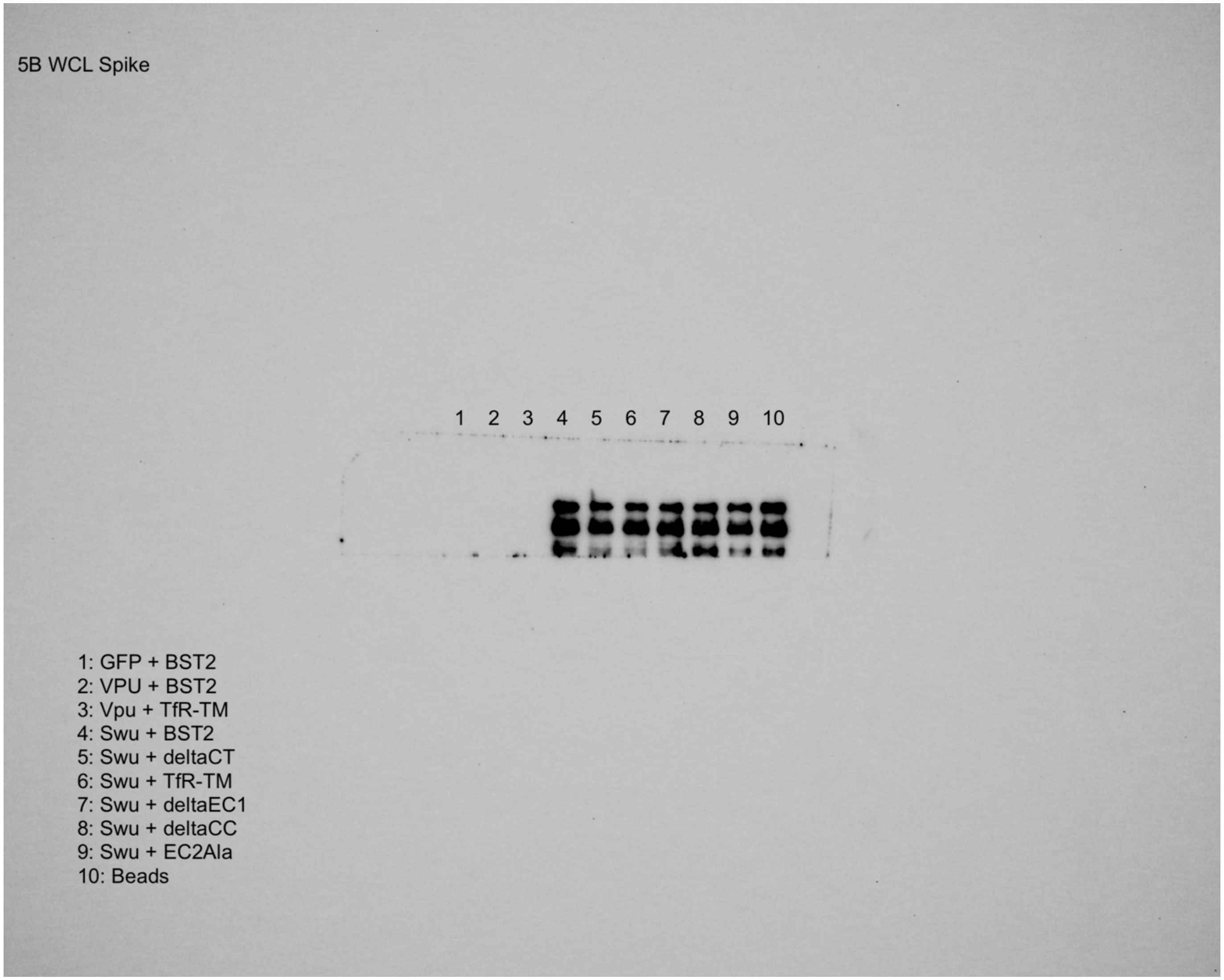
5B WCL Spike
1 2 3 4 5 6 7 8 9 10
1: GFP + BST2
2: VPU + BST2
3: Vpu + TfR-TM
4: Swu + BST2
5: Swu + deltaCT
6: Swu + TfR-TM
7: Swu + deltaEC1
8: Swu + deltaCC
9: Swu + EC2Ala
10: Beads

5B WCL HA-BST2

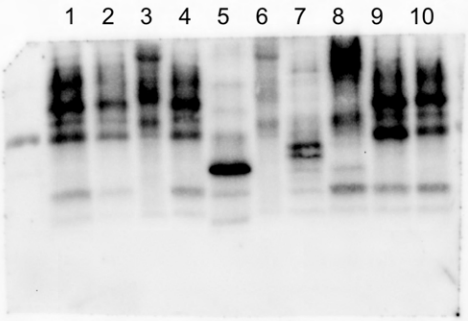

1: GFP + BST2
2: VPU + BST2
3: Vpu + TfR-TM
4: Swu + BST2
5: Swu + deltaCT
6: Swu + TfR-TM
7: Swu + deltaEC1
8: Swu + deltaCC
9: Swu + EC2Ala
10: Beads

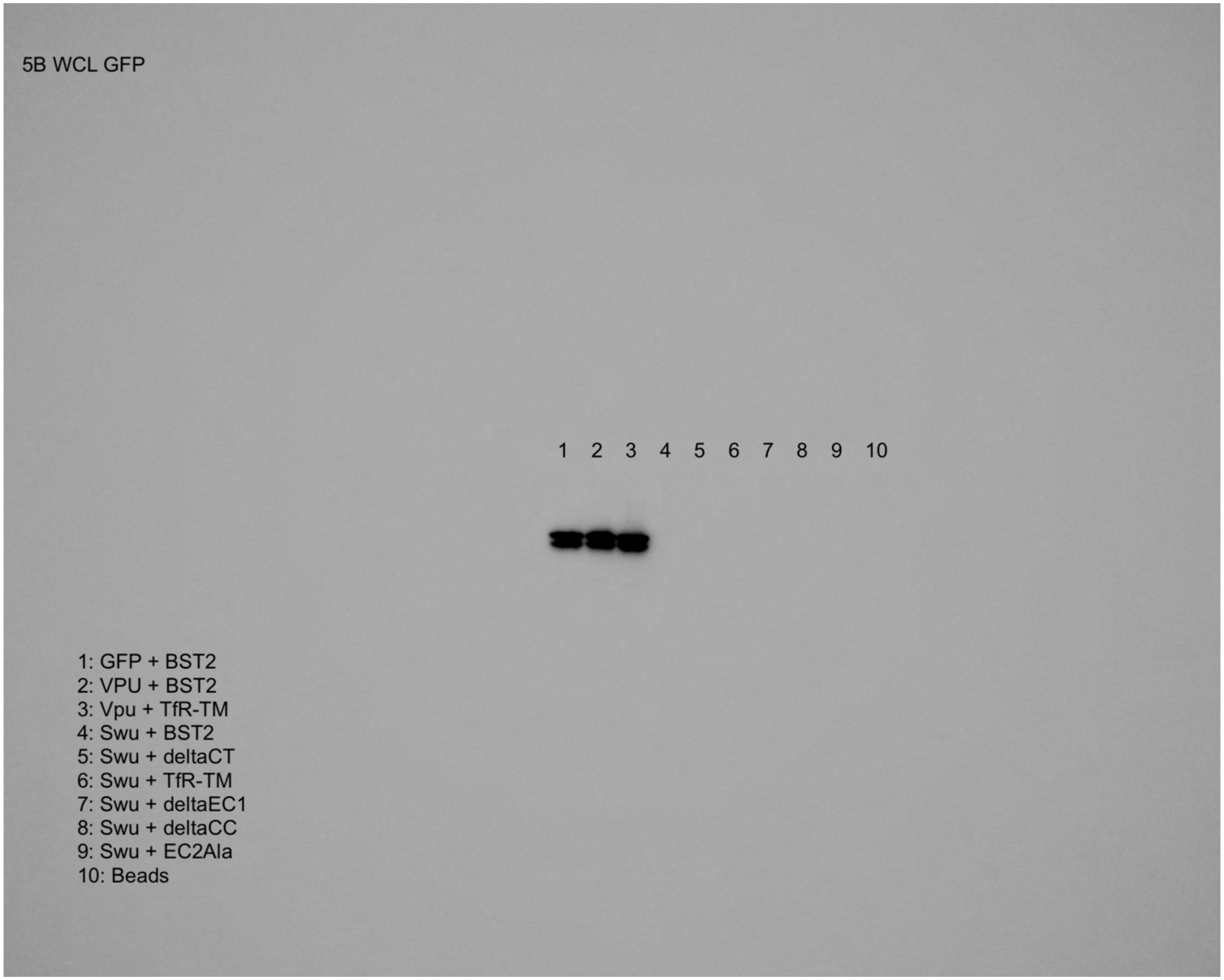
5B WCL GFP
1 2 3 4 5 6 7 8 9 10
1: GFP + BST2
2: VPU + BST2
3: Vpu + TfR-TM
4: Swu + BST2
5: Swu + deltaCT
6: Swu + TfR-TM
7: Swu + deltaEC1
8: Swu + deltaCC
9: Swu + EC2Ala
10: Beads

5B WCL VPU

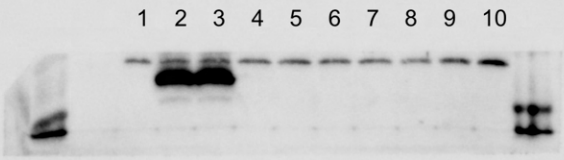

1: GFP + BST2
2: VPU + BST2
3: Vpu + TfR-TM
4: Swu + BST2
5: Swu + deltaCT
6: Swu + TfR-TM
7: Swu + deltaEC1
8: Swu + deltaCC
9: Swu + EC2Ala
10: Beads

5B WCL Actin

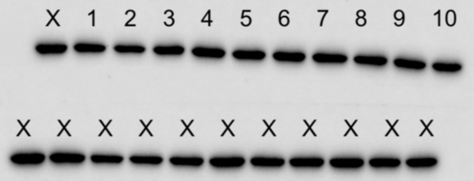

1: GFP + BST2
2: VPU + BST2
3: Vpu + TfR-TM
4: Swu + BST2
5: Swu + deltaCT
6: Swu + TfR-TM
7: Swu + deltaEC1
8: Swu + deltaCC
9: Swu + EC2Ala
10: Beads

5C IP Spike

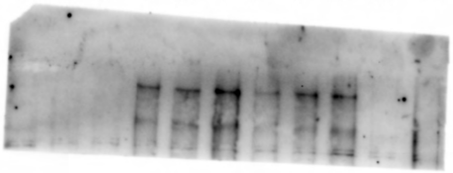

1: GFP + BST2
2: VPU + BST2
3: Vpu + TfR-TM
4: Somi + BST2
5: Somi + deltaCT
6: Somi + TfR-TM
7: Somi + deltaEC1
8: Somi + deltaCC
9: Somi + EC2Ala
10: Beads
11: IgG

5C IP HA-BST2

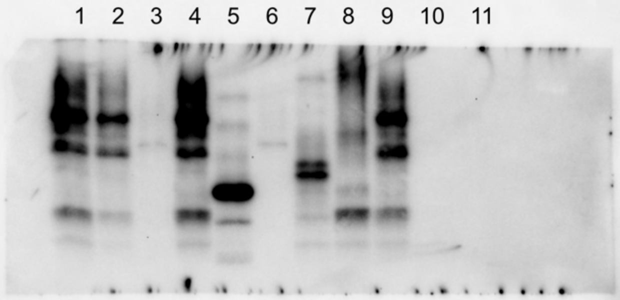

1: GFP + BST2
2: VPU + BST2
3: Vpu + TfR-TM
4: Somi + BST2
5: Somi + deltaCT
6: Somi + TfR-TM
7: Somi + deltaEC1
8: Somi + deltaCC
9: Somi + EC2Ala
10: Beads
11: IgG

5C IP GFP

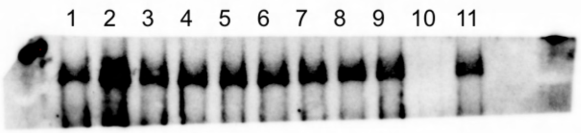

1: GFP + BST2
2: VPU + BST2
3: Vpu + TfR-TM
4: Somi + BST2
5: Somi + deltaCT
6: Somi + TfR-TM
7: Somi + deltaEC1
8: Somi + deltaCC
9: Somi + EC2Ala
10: Beads
11: IgG

5C IP VPU

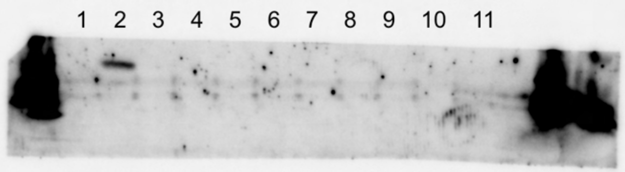

1: GFP + BST2
2: VPU + BST2
3: Vpu + TfR-TM
4: Somi + BST2
5: Somi + deltaCT
6: Somi + TfR-TM
7: Somi + deltaEC1
8: Somi + deltaCC
9: Somi + EC2Ala
10: Beads
11: IgG

5C WCL Spike

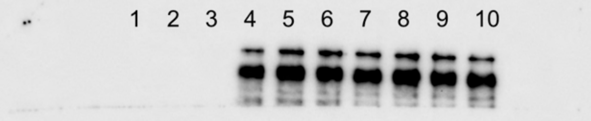

1: GFP + BST2
2: VPU + BST2
3: Vpu + TfR-TM
4: Somi + BST2
5: Somi + deltaCT
6: Somi + TfR-TM
7: Somi + deltaEC1
8: Somi + deltaCC
9: Somi + EC2Ala
10: Beads

5C WCL HA-BST2

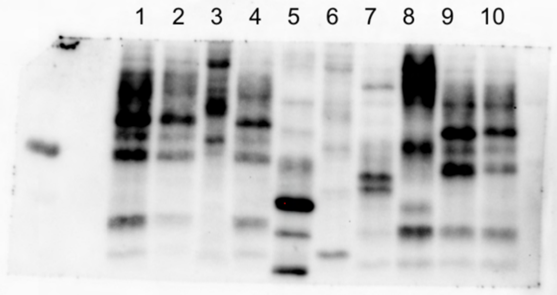

1: GFP + BST2
2: VPU + BST2
3: Vpu + TfR-TM
4: Somi + BST2
5: Somi + deltaCT
6: Somi + TfR-TM
7: Somi + deltaEC1
8: Somi + deltaCC
9: Somi + EC2Ala
10: Beads

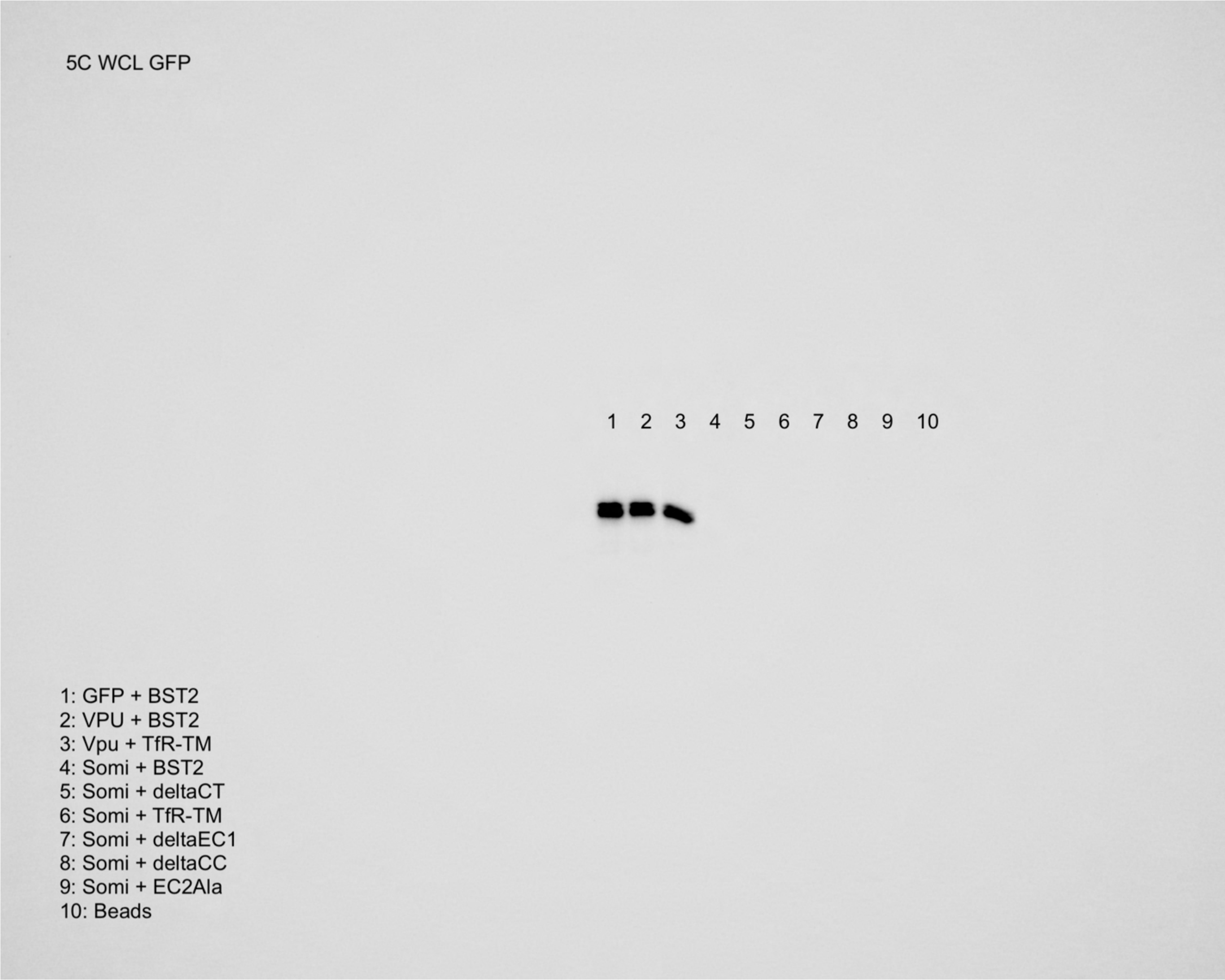
5C WCL GFP
1 2 3 4 5 6 7 8 9 10
1: GFP + BST2
2: VPU + BST2
3: Vpu + TfR-TM
4: Somi + BST2
5: Somi + deltaCT
6: Somi + TfR-TM
7: Somi + deltaEC1
8: Somi + deltaCC
9: Somi + EC2Ala
10: Beads

5C WCL VPU

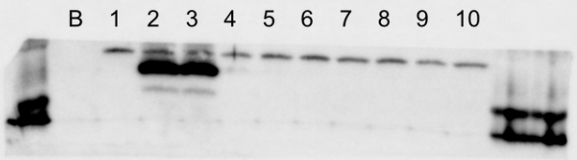

1: GFP + BST2
2: VPU + BST2
3: Vpu + TfR-TM
4: Somi + BST2
5: Somi + deltaCT
6: Somi + TfR-TM
7: Somi + deltaEC1
8: Somi + deltaCC
9: Somi + EC2Ala
10: Beads
B: Blank

5C WCL Actin

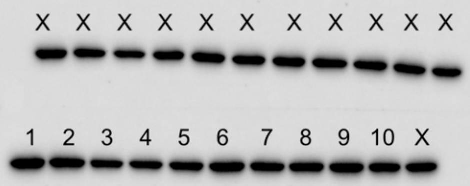

1: GFP + BST2
2: VPU + BST2
3: Vpu + TfR-TM
4: Somi + BST2
5: Somi + deltaCT
6: Somi + TfR-TM
7: Somi + deltaEC1
8: Somi + deltaCC
9: Somi + EC2Ala
10: Beads

8A, Spike

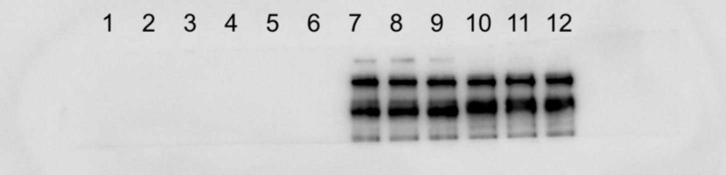

1: GST + DMSO
2: GST + Chlor
3: GST + MG132
4: Vpu + DMSO
5: Vpu + Chlor
6: Vpu + MG132
7: Swuhan + DMSO
8: Swuhan + Chlor
9: Swuhan + MG132
10: Somicron + DMSO
11: Somicron + Chlor
12: Somicron + MG132

8A, BST2

1: GST + DMSO
2: GST + Chlor
3: GST + MG132
4: Vpu + DMSO
5: Vpu + Chlor
6: Vpu + MG132
7: Swuhan + DMSO
8: Swuhan + Chlor
9: Swuhan + MG132
10: Somicron + DMSO
11: Somicron + Chlor
12: Somicron + MG132

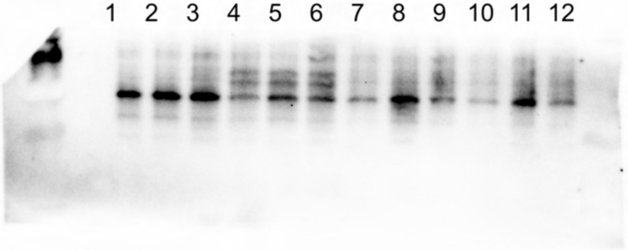

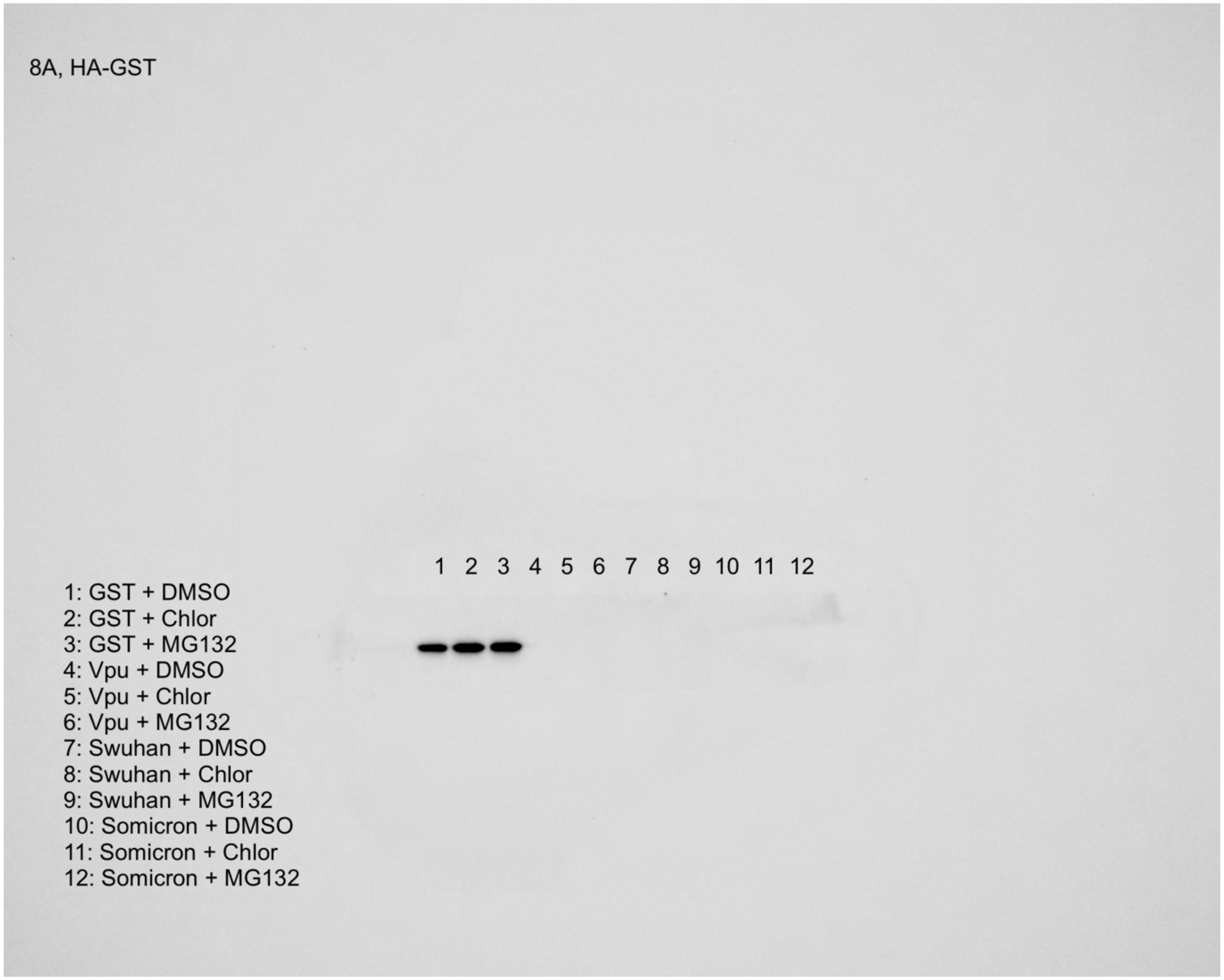
8A, HA-GST
1 2 3 4 5 6 7 8 9 10 11 12
1: GST + DMSO
2: GST + Chlor
3: GST + MG132
4: Vpu + DMSO
5: Vpu + Chlor
6: Vpu + MG132
7: Swuhan + DMSO
8: Swuhan + Chlor
9: Swuhan + MG132
10: Somicron + DMSO
11: Somicron + Chlor
12: Somicron + MG132

8A, Vpu

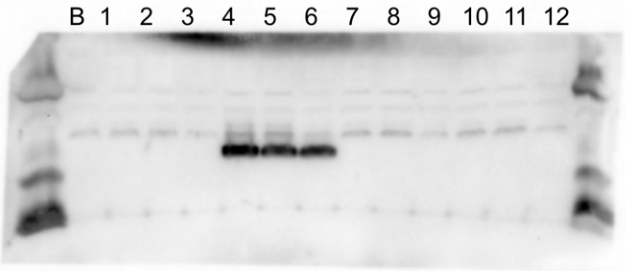

B: Blank
1: GST + DMSO
2: GST + Chlor
3: GST + MG132
4: Vpu + DMSO
5: Vpu + Chlor
6: Vpu + MG132
7: Swuhan + DMSO
8: Swuhan + Chlor
9: Swuhan + MG132
10: Somicron + DMSO
11: Somicron + Chlor
12: Somicron + MG132

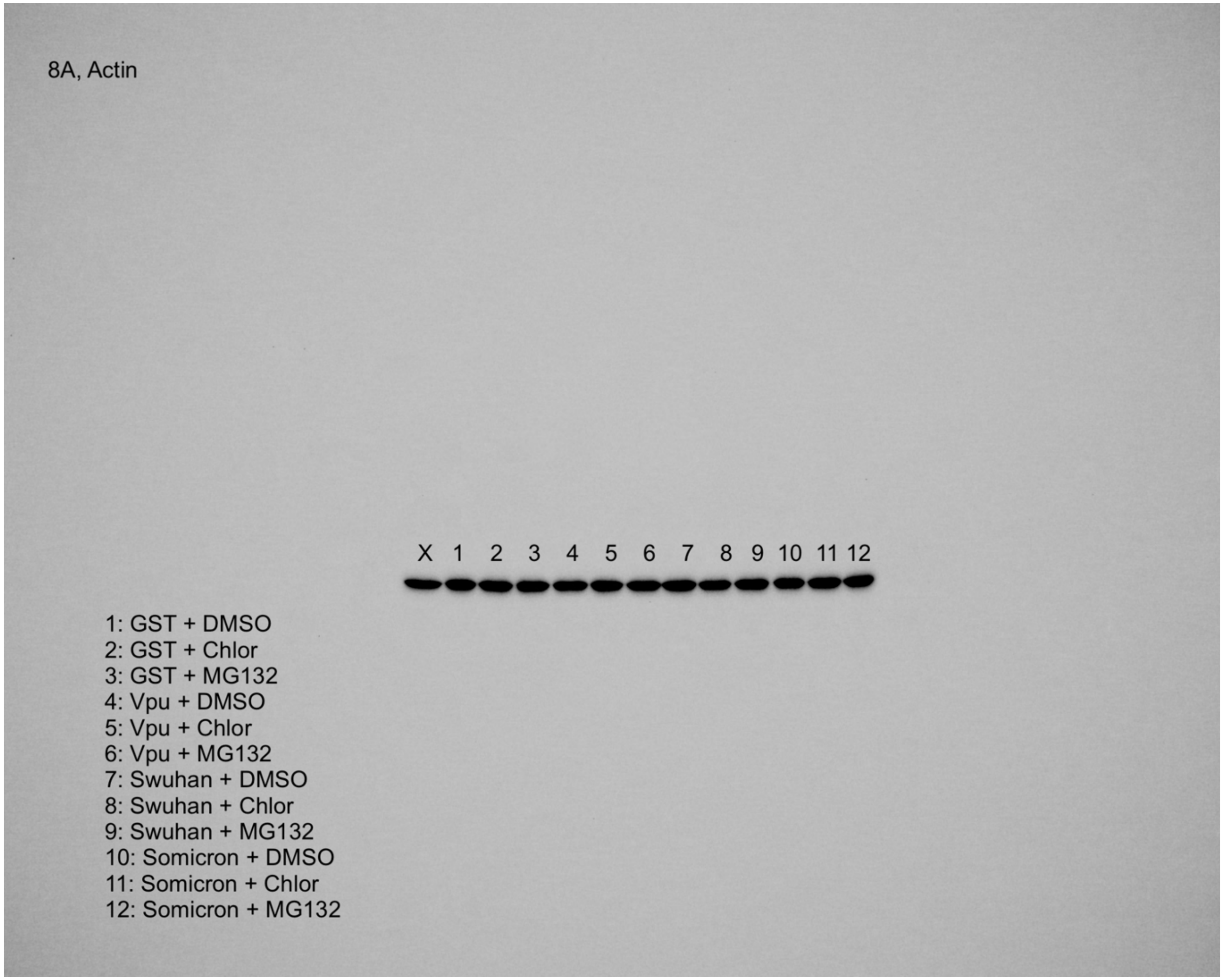
8A, Actin
X 1 2 3 4 5 6 7 8 9 10 11 12
1: GST + DMSO
2: GST + Chlor
3: GST + MG132
4: Vpu + DMSO
5: Vpu + Chlor
6: Vpu + MG132
7: Swuhan + DMSO
8: Swuhan + Chlor
9: Swuhan + MG132
10: Somicron + DMSO
11: Somicron + Chlor
12: Somicron + MG132

9A and 9C, Spike

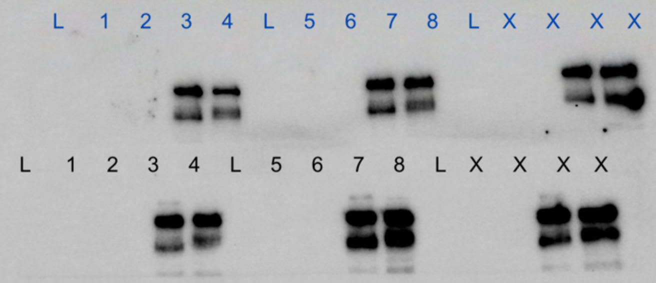

1: GST-HA + DMSO
2: Vpu+ DMSO
3: Wuhan Spike+ DMSO
4: Omicron Spike+ DMSO
5: GST-HA + TAK-243
6: Vpu+ TAK-243
7: Wuhan Spike+ TAK-243
8: Omicron Spike+ TAK-243

1: GST-HA + pCGCG
2: Vpu+ pCGCG
3: Wuhan Spike+ pCGCG
4: Omicron Spike+ pCGCG
5: GST-HA + AP180C
6: Vpu+ AP180C
7: Wuhan Spike+ AP180C
8: Omicron Spike+ AP180C

9A, BST2

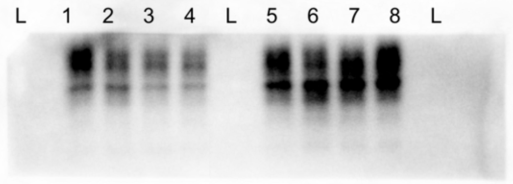

1: GST-HA + DMSO
2: Vpu+ DMSO
3: Wuhan Spike+ DMSO
4: Omicron Spike+ DMSO
5: GST-HA + TAK-243
6: Vpu+ TAK-243
7: Wuhan Spike+ TAK-243
8: Omicron Spike+ TAK-243

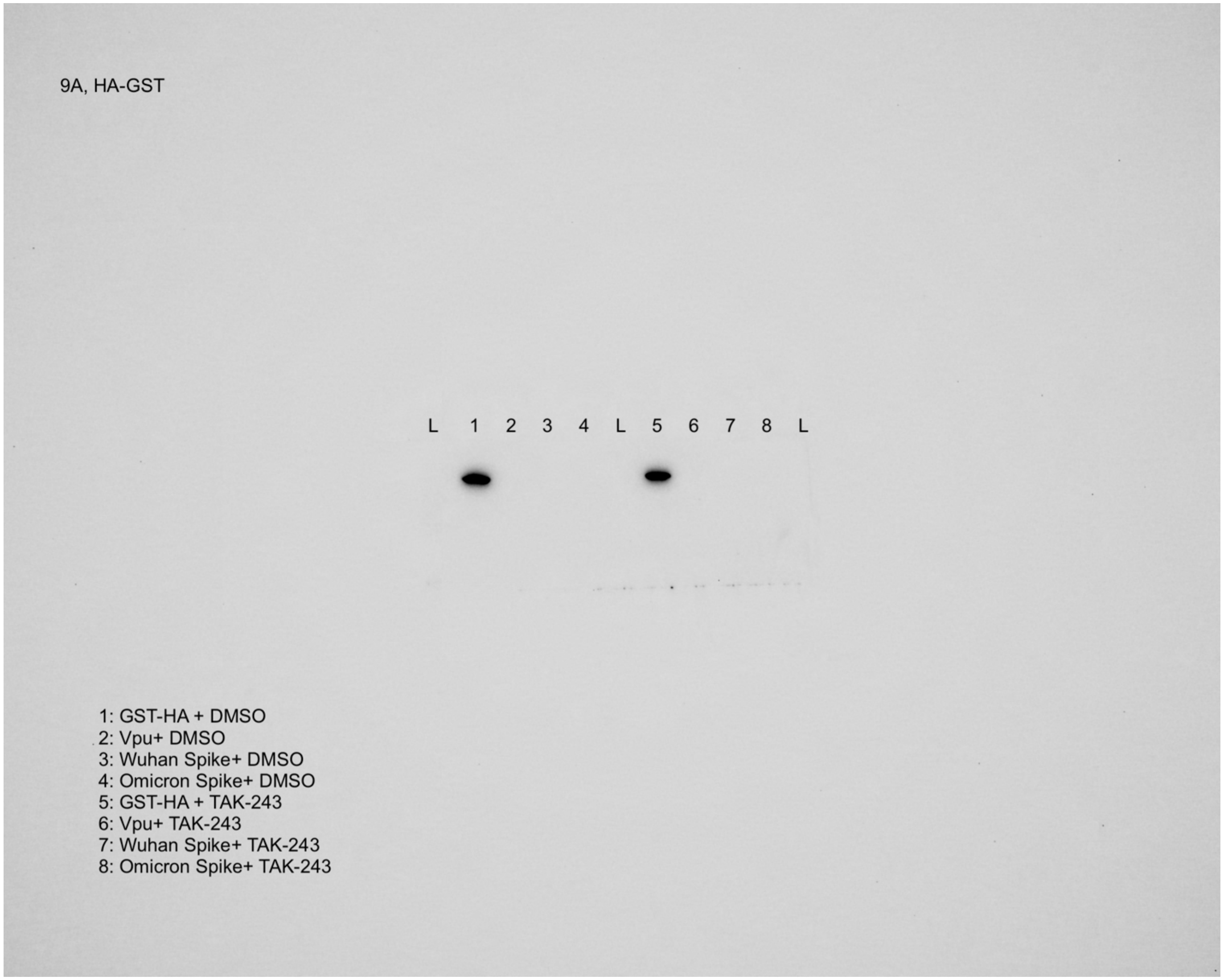
9A, HA-GST
L 1 2 3 4 L 5 6 7 8 L
1: GST-HA + DMSO
2: Vpu+ DMSO
3: Wuhan Spike+ DMSO
4: Omicron Spike+ DMSO
5: GST-HA + TAK-243
6: Vpu+ TAK-243
7: Wuhan Spike+ TAK-243
8: Omicron Spike+ TAK-243

9A, Vpu

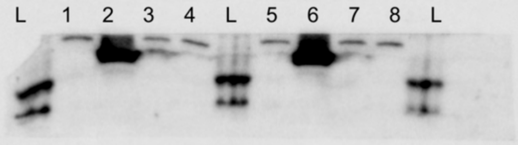

1: GST-HA + DMSO
2: Vpu+ DMSO
3: Wuhan Spike+ DMSO
4: Omicron Spike+ DMSO
5: GST-HA + TAK-243
6: Vpu+ TAK-243
7: Wuhan Spike+ TAK-243
8: Omicron Spike+ TAK-243

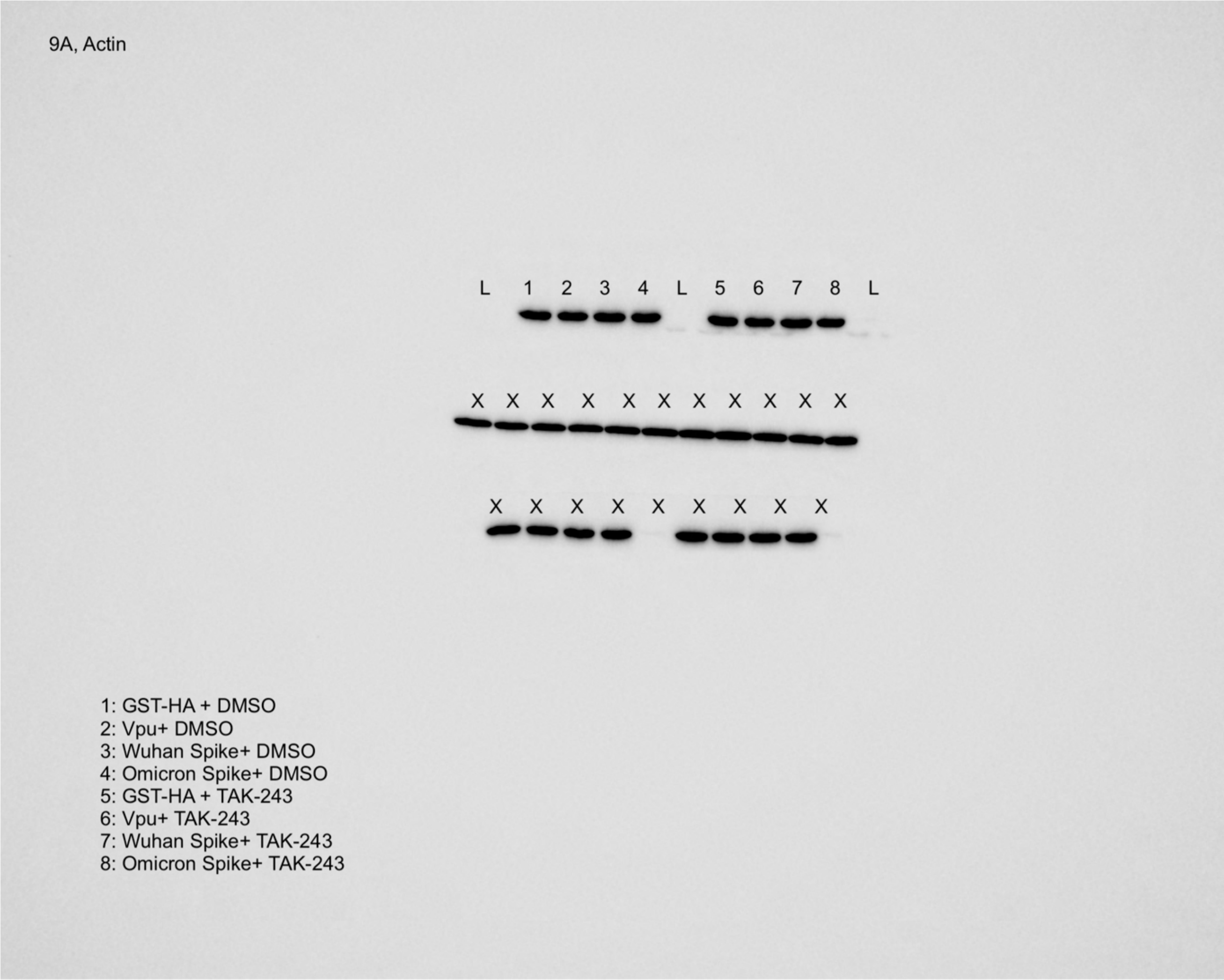
9A, Actin
L 1 2 3 4 L 5 6 7 8 L
X X X X X X X X X X X
X X X X X X X X X
1: GST-HA + DMSO
2: Vpu+ DMSO
3: Wuhan Spike+ DMSO
4: Omicron Spike+ DMSO
5: GST-HA + TAK-243
6: Vpu+ TAK-243
7: Wuhan Spike+ TAK-243
8: Omicron Spike+ TAK-243

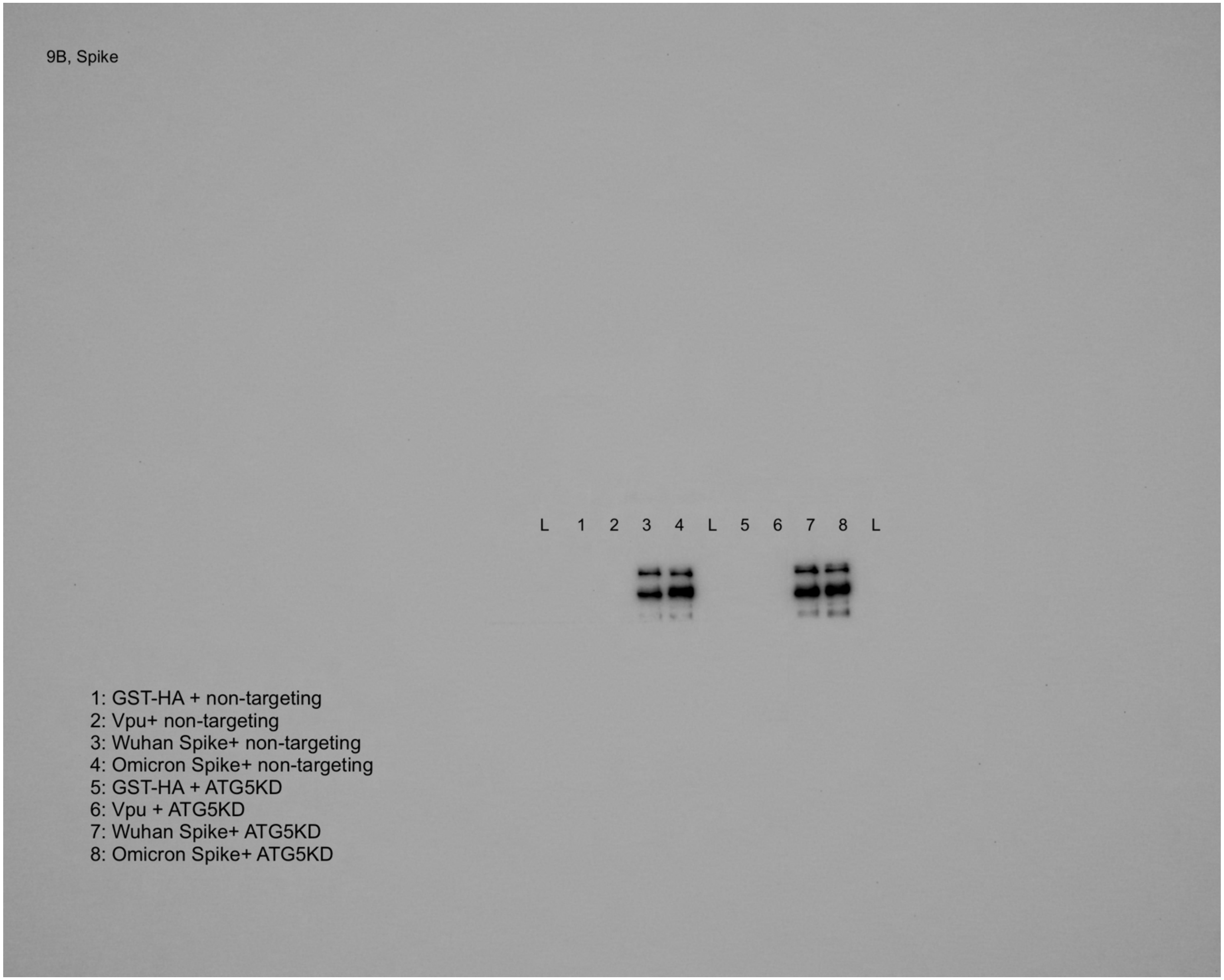
9B, Spike
L 1 2 3 4 L 5 6 7 8 L
1: GST-HA + non-targeting
2: Vpu+ non-targeting
3: Wuhan Spike+ non-targeting
4: Omicron Spike+ non-targeting
5: GST-HA + ATG5KD
6: Vpu + ATG5KD
7: Wuhan Spike+ ATG5KD
8: Omicron Spike+ ATG5KD

9B, BST2

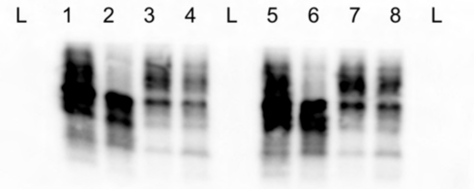

1: GST-HA + non-targeting
2: Vpu+ non-targeting
3: Wuhan Spike+ non-targeting
4: Omicron Spike+ non-targeting
5: GST-HA + ATG5KD
6: Vpu + ATG5KD
7: Wuhan Spike+ ATG5KD
8: Omicron Spike+ ATG5KD

9B, ATG5

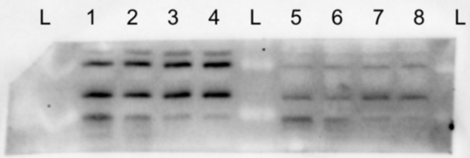

1: GST-HA + non-targeting
2: Vpu+ non-targeting
3: Wuhan Spike+ non-targeting
4: Omicron Spike+ non-targeting
5: GST-HA + ATG5KD
6: Vpu + ATG5KD
7: Wuhan Spike+ ATG5KD
8: Omicron Spike+ ATG5KD

9B, LC3

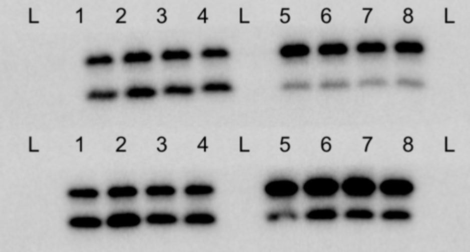

1: GST-HA + non-targeting
2: Vpu+ non-targeting
3: Wuhan Spike+ non-targeting
4: Omicron Spike+ non-targeting
5: GST-HA + ATG5KD
6: Vpu + ATG5KD
7: Wuhan Spike+ ATG5KD
8: Omicron Spike+ ATG5KD
2X replicates

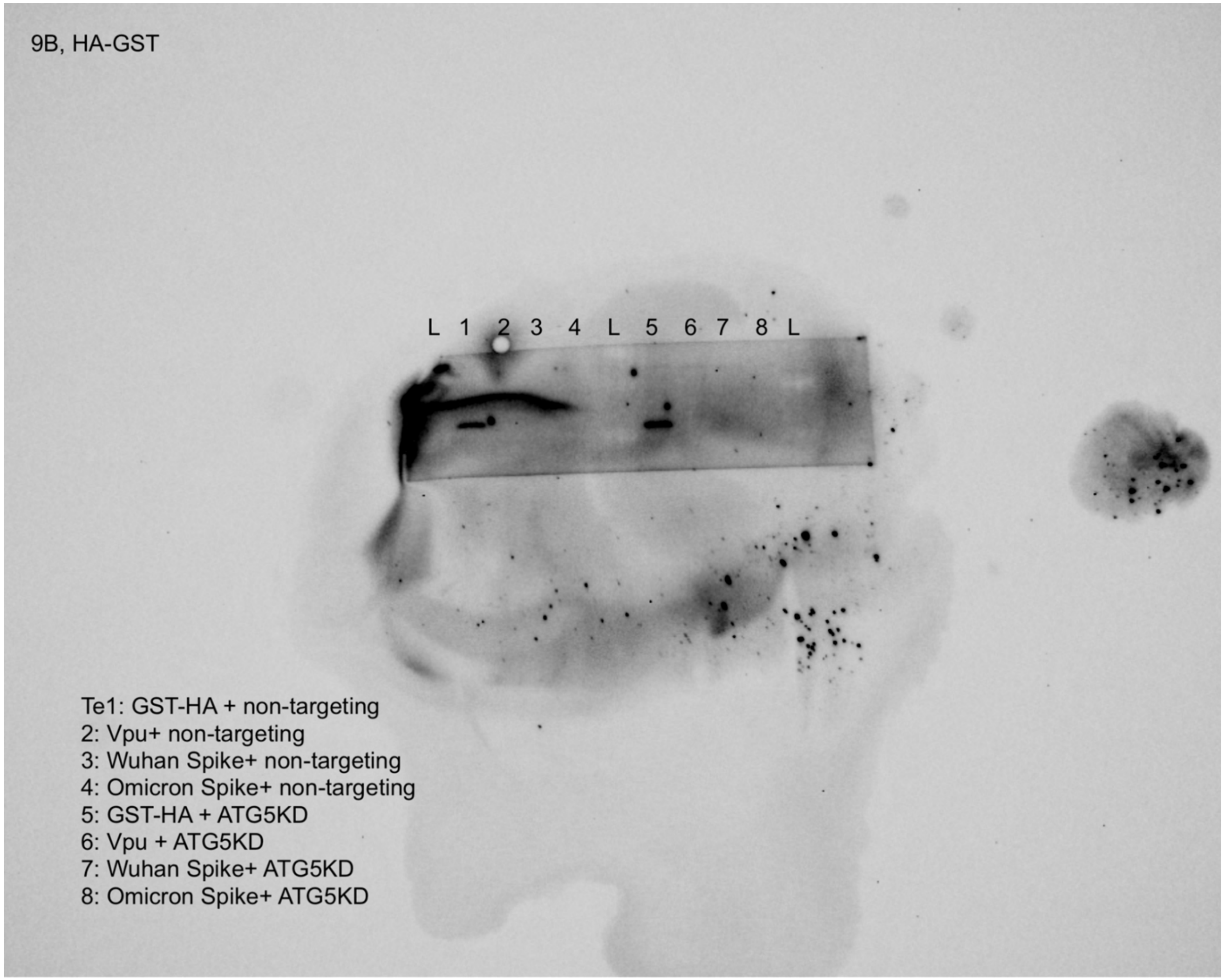
9B, HA-GST
L 1 2 3 4 L 5 6 7 8 L
Te1: GST-HA + non-targeting
2: Vpu+ non-targeting
3: Wuhan Spike+ non-targeting
4: Omicron Spike+ non-targeting
5: GST-HA + ATG5KD
6: Vpu + ATG5KD
7: Wuhan Spike+ ATG5KD
8: Omicron Spike+ ATG5KD

9B, Vpu

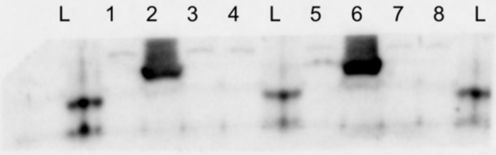

1: GST-HA + non-targeting
2: Vpu+ non-targeting
3: Wuhan Spike+ non-targeting
4: Omicron Spike+ non-targeting
5: GST-HA + ATG5KD
6: Vpu + ATG5KD
7: Wuhan Spike+ ATG5KD
8: Omicron Spike+ ATG5KD

9B, Actin

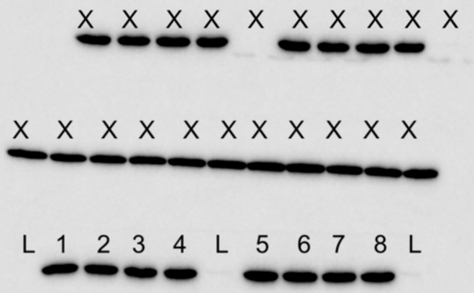

1: GST-HA + non-targeting
2: Vpu+ non-targeting
3: Wuhan Spike+ non-targeting
4: Omicron Spike+ non-targeting
5: GST-HA + ATG5KD
6: Vpu + ATG5KD
7: Wuhan Spike+ ATG5KD
8: Omicron Spike+ ATG5KD

9C, AP180C-Flag

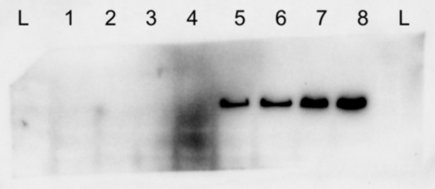

1: GST-HA + pCGCG
2: Vpu+ pCGCG
3: Wuhan Spike+ pCGCG
4: Omicron Spike+ pCGCG
5: GST-HA + AP180C
6: Vpu+ AP180C
7: Wuhan Spike+ AP180C
8: Omicron Spike+ AP180C

9C, BST2

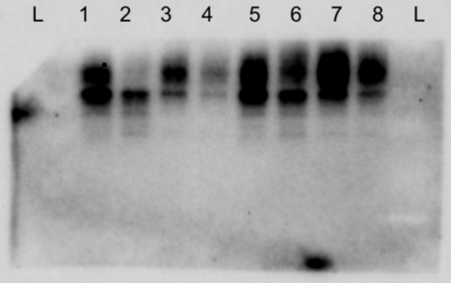

1: GST-HA + pCGCG
2: Vpu+ pCGCG
3: Wuhan Spike+ pCGCG
4: Omicron Spike+ pCGCG
5: GST-HA + AP180C
6: Vpu+ AP180C
7: Wuhan Spike+ AP180C
8: Omicron Spike+ AP180C

9C, HA-GST

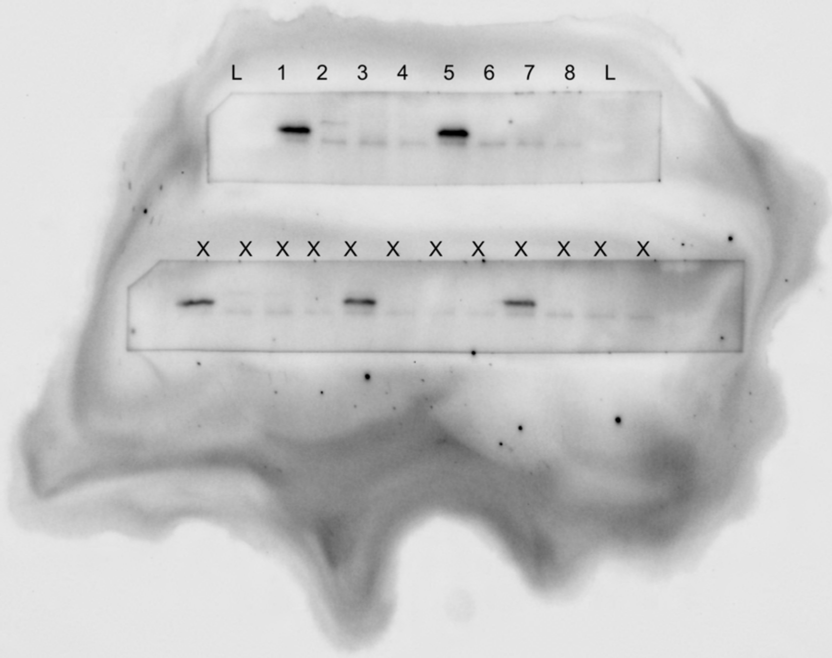

1: GST-HA + pCGCG
2: Vpu+ pCGCG
3: Wuhan Spike+ pCGCG
4: Omicron Spike+ pCGCG
5: GST-HA + AP180C
6: Vpu+ AP180C
7: Wuhan Spike+ AP180C
8: Omicron Spike+ AP180C

9C, GFP

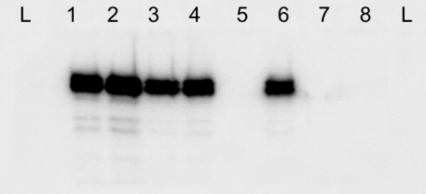

1: GST-HA + pCGCG
2: Vpu+ pCGCG
3: Wuhan Spike+ pCGCG
4: Omicron Spike+ pCGCG
5: GST-HA + AP180C
6: Vpu+ AP180C
7: Wuhan Spike+ AP180C
8: Omicron Spike+ AP180C

9C, Vpu

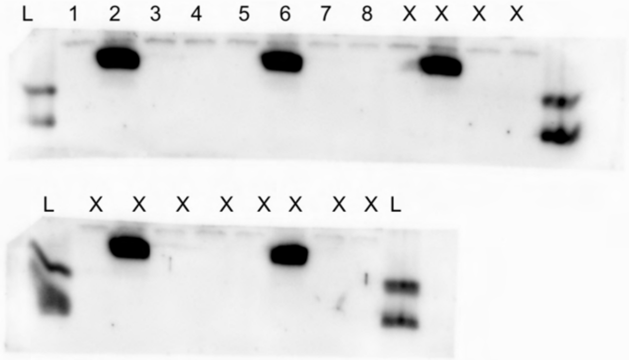

1: GST-HA + pCGCG
2: Vpu+ pCGCG
3: Wuhan Spike+ pCGCG
4: Omicron Spike+ pCGCG
5: GST-HA + AP180C
6: Vpu+ AP180C
7: Wuhan Spike+ AP180C
8: Omicron Spike+ AP180C

9C, Actin

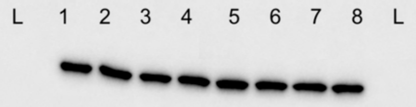

1: GST-HA + pCGCG
2: Vpu+ pCGCG
3: Wuhan Spike+ pCGCG
4: Omicron Spike+ pCGCG
5: GST-HA + AP180C
6: Vpu+ AP180C
7: Wuhan Spike+ AP180C
8: Omicron Spike+ AP180C

S1 BST2

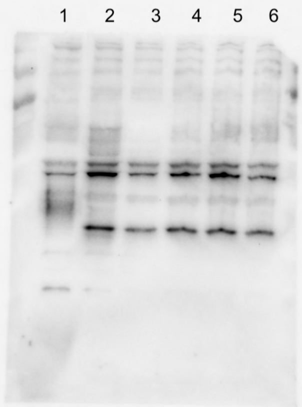

1: HeLa
2: A549-ACE2-BST2
3: A549 NO IFN
4: A549 100 U/ML IFN
5: A549: 1,000 U/ML IFN
6: A549 10,000 U/ML IFN

S1, ACTIN

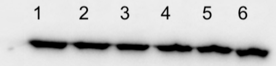

1: HeLa
2: A549-ACE2-BST2
3: A549 NO IFN
4: A549 100 U/ML IFN
5: A549: 1,000 U/ML IFN
6: A549 10,000 U/ML IFN

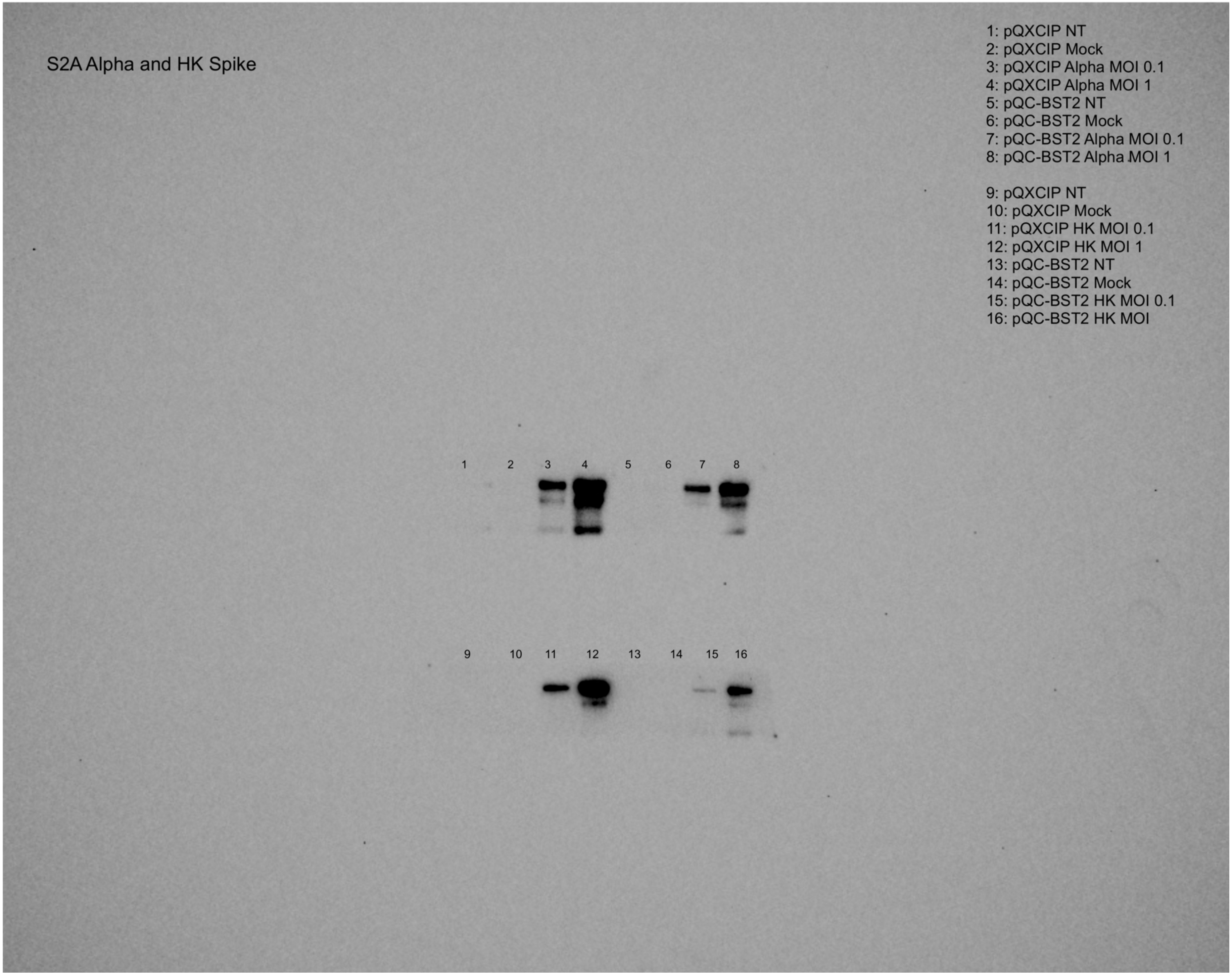
S2A Alpha and HK Spike
1: pQXCIP NT
2: pQXCIP Mock
3: pQXCIP Alpha MOI 0.1
4: pQXCIP Alpha MOI 1
5: pQC-BST2 NT
6: pQC-BST2 Mock
7: pQC-BST2 Alpha MOI 0.1
8: pQC-BST2 Alpha MOI 1
9: pQXCIP NT
10: pQXCIP Mock
11: pQXCIP HK MOI 0.1
12: pQXCIP HK MOI 1
13: pQC-BST2 NT
14: pQC-BST2 Mock
15: pQC-BST2 HK MOI 0.1
16: pQC-BST2 HK MOI
1 2 3 4 5 6 7 8
9 10 11 12 13 14 15 16

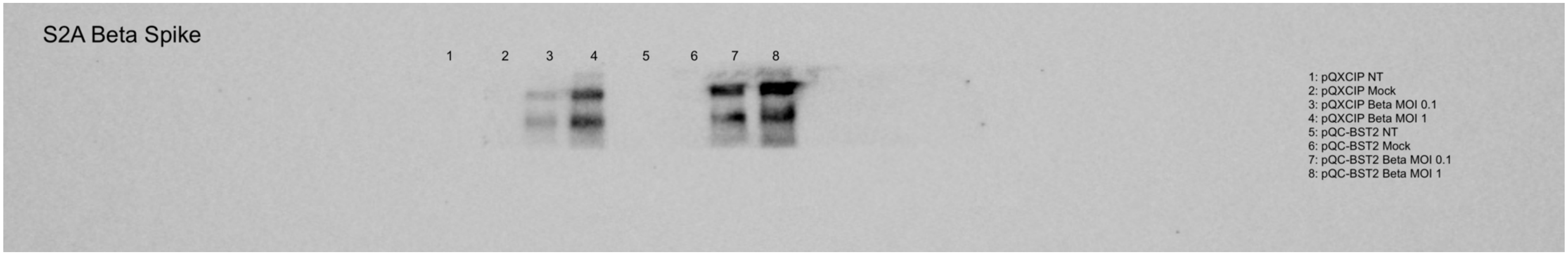
S2A Beta Spike
1 2 3 4 5 6 7 8
1: pQXCIP NT
2: pQXCIP Mock
3: pQXCIP Beta MOI 0.1
4: pQXCIP Beta MOI 1
5: pQC-BST2 NT
6: pQC-BST2 Mock
7: pQC-BST2 Beta MOI 0.1
8: pQC-BST2 Beta MOI 1

S2A HK and Alpha N

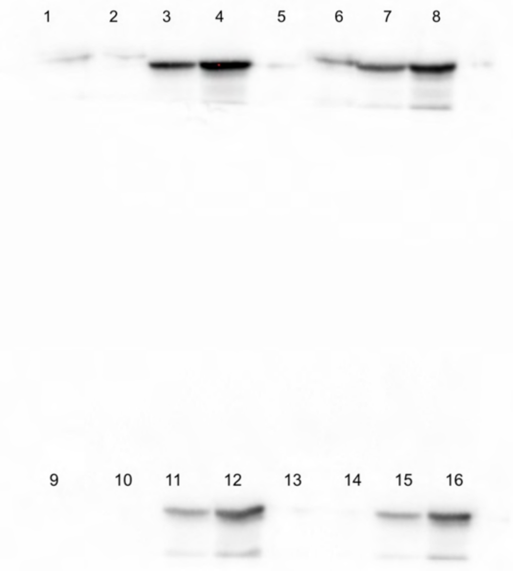

1: pQXCIP NT
2: pQXCIP Mock
3: pQXCIP HK MOI 0.1
4: pQXCIP HK MOI 1
5: pQC-BST2 NT
6: pQC-BST2 Mock
7: pQC-BST2 HK MOI 0.1
8: pQC-BST2 HK MOI 1

9: pQXCIP NT
10: pQXCIP Mock
11: pQXCIP Alpha MOI 0.1
12: pQXCIP Alpha MOI 1
13: pQC-BST2 NT
14: pQC-BST2 Mock
15: pQC-BST2 Alpha MOI 0.1
16: pQC-BST2 Alpha MOI 1

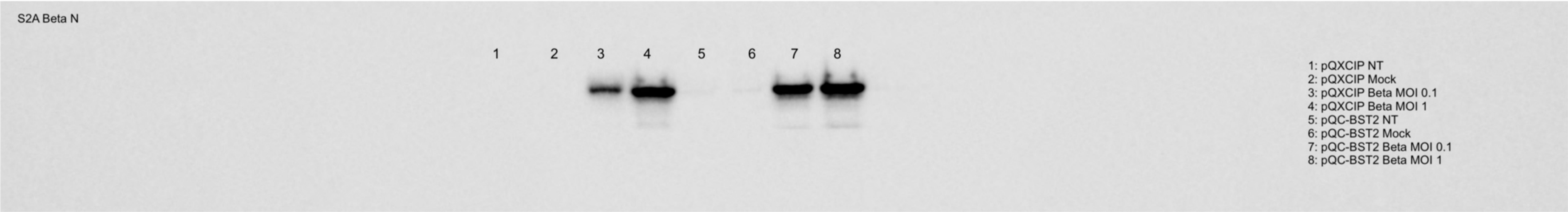
S2A Beta N
1
2
3
4
5
6
7
8
1: pQXCIP NT
2: pQXCIP Mock
3: pQXCIP Beta MOI 0.1
4: pQXCIP Beta MOI 1
5: pQC-BST2 NT
6: pQC-BST2 Mock
7: pQC-BST2 Beta MOI 0.1
8: pQC-BST2 Beta MOI 1

S2A HK and Alpha BST2

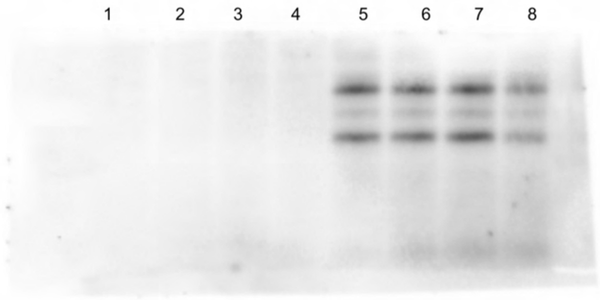

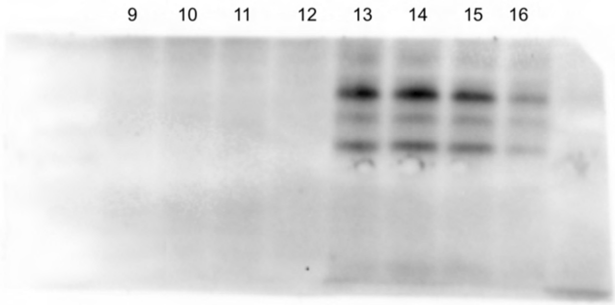

1: pQXCIP NT
2: pQXCIP Mock
3: pQXCIP HK MOI 0.1
4: pQXCIP HK MOI 1
5: pQC-BST2 NT
6: pQC-BST2 Mock
7: pQC-BST2 HK MOI 0.1
8: pQC-BST2 HK MOI 1

9: pQXCIP NT
10: pQXCIP Mock
11: pQXCIP Alpha MOI 0.1
12: pQXCIP Alpha MOI 1
13: pQC-BST2 NT
14: pQC-BST2 Mock
15: pQC-BST2 Alpha MOI 0.1
16: pQC-BST2 Alpha MOI 1

S2A Beta, BST2

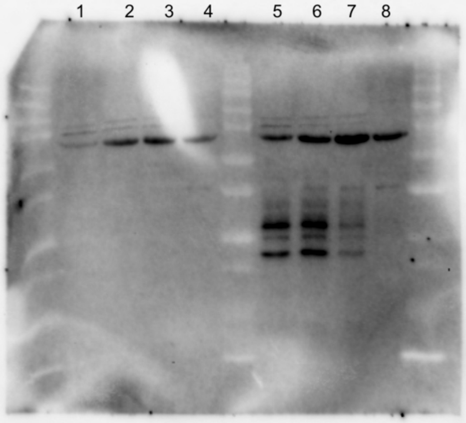

1: pQXCIP NT
2: pQXCIP Mock
3: pQXCIP Beta MOI 0.1
4: pQXCIP Beta MOI 1
5: pQC-BST2 NT
6: pQC-BST2 Mock
7: pQC-BST2 Beta MOI 0.1
8: pQC-BST2 Beta MOI 1

S2A HK and Beta actin

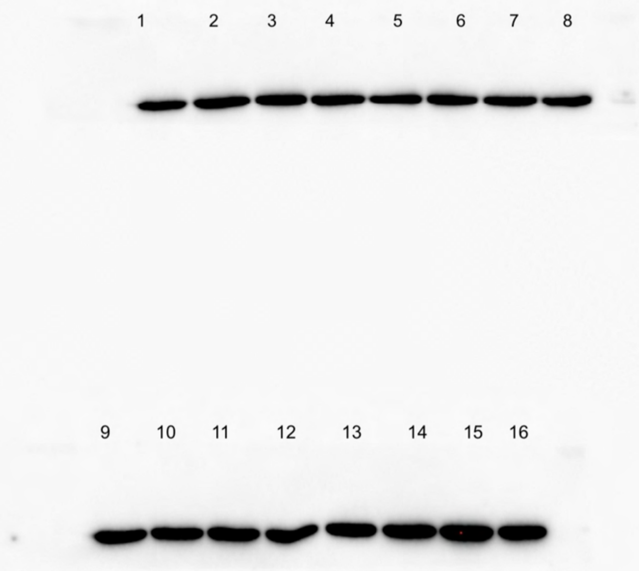

1: pQXCIP NT
2: pQXCIP Mock
3: pQXCIP HK MOI 0.1
4: pQXCIP HK MOI 1
5: pQC-BST2 NT
6: pQC-BST2 Mock
7: pQC-BST2 HK MOI 0.1
8: pQC-BST2 HK MOI 1

9: pQXCIP NT
10: pQXCIP Mock
11: pQXCIP Beta MOI 0.1
12: pQXCIP Beta MOI 1
13: pQC-BST2 NT
14: pQC-BST2 Mock
15: pQC-BST2 Beta MOI 0.1
16: pQC-BST2 Beta MOI 1

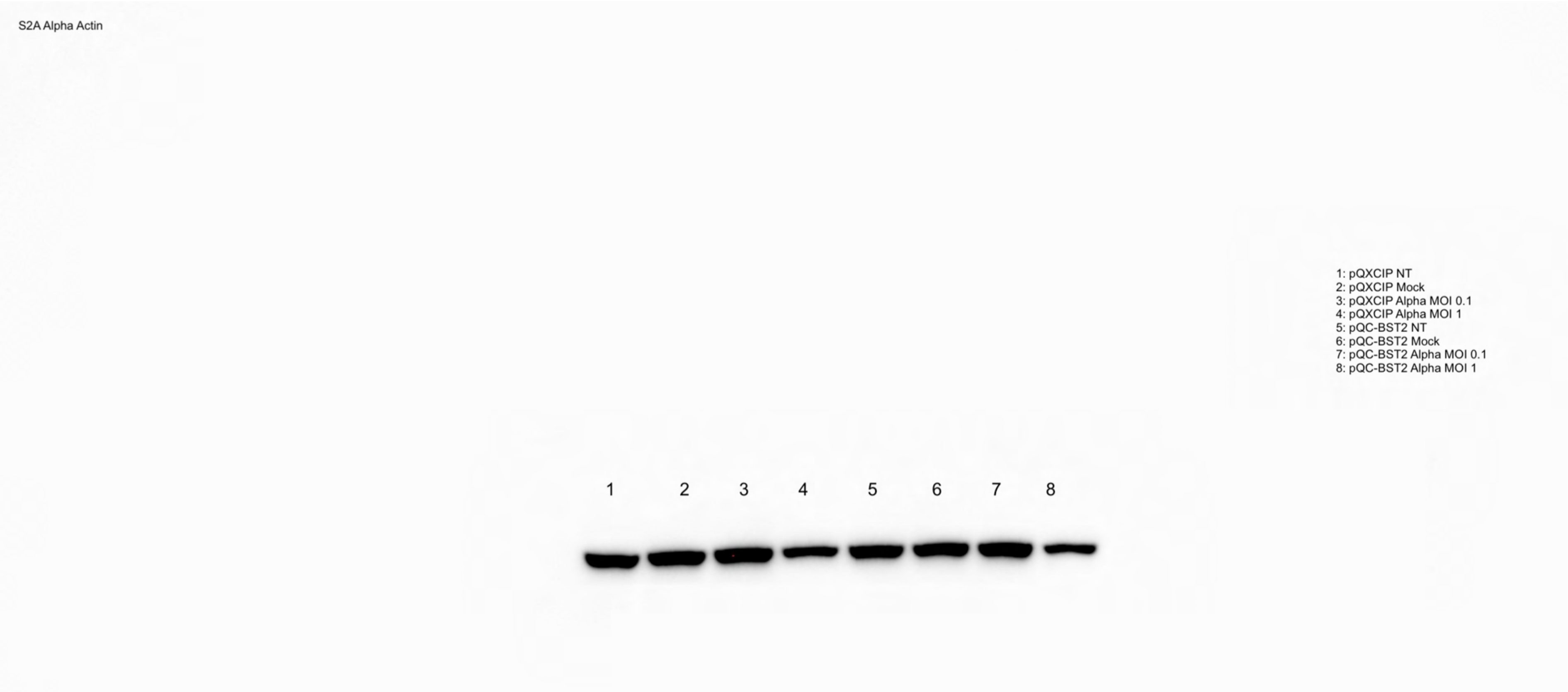
S2A Alpha Actin
1
2
3
4
5
6
7
8
1: pQXCIP NT
2: pQXCIP Mock
3: pQXCIP Alpha MOI 0.1
4: pQXCIP Alpha MOI 1
5: pQC-BST2 NT
6: pQC-BST2 Mock
7: pQC-BST2 Alpha MOI 0.1
8: pQC-BST2 Alpha MOI 1

S2A, Delta Spike

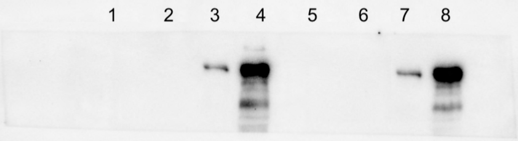

1: pQXCIP NT
2: pQXCIP Mock
3: pQXCIP Delta MOI 0.1
4: pQXCIP Delta MOI 1
5: pQC-BST2 NT
6: pQC-BST2 Mock
7: pQC-BST2 Delta MOI 0.1
8: pQC-BST2 Delta MOI 1

S1A, Delta N

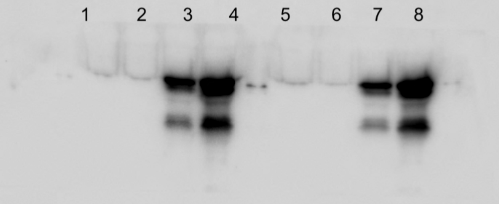

1: pQXCIP NT
2: pQXCIP Mock
3: pQXCIP Delta MOI 0.1
4: pQXCIP Delta MOI 1
5: pQC-BST2 NT
6: pQC-BST2 Mock
7: pQC-BST2 Delta MOI 0.1
8: pQC-BST2 Delta MOI 1

S2A, Delta BST2

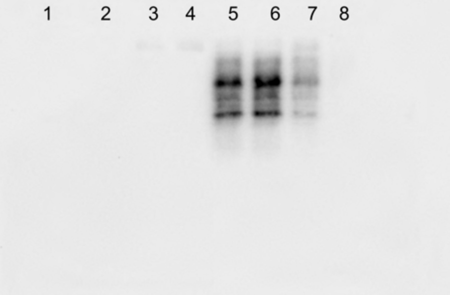

1: pQXCIP NT
2: pQXCIP Mock
3: pQXCIP Delta MOI 0.1
4: pQXCIP Delta MOI 1
5: pQC-BST2 NT
6: pQC-BST2 Mock
7: pQC-BST2 Delta MOI 0.1
8: pQC-BST2 Delta MOI 1

S2A, Delta Actin

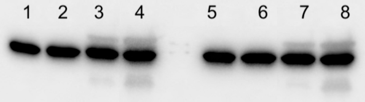

1: pQXCIP NT
2: pQXCIP Mock
3: pQXCIP Delta MOI 0.1
4: pQXCIP Delta MOI 1
5: pQC-BST2 NT
6: pQC-BST2 Mock
7: pQC-BST2 Delta MOI 0.1
8: pQC-BST2 Delta MOI 1

S2A, Omicron Spike

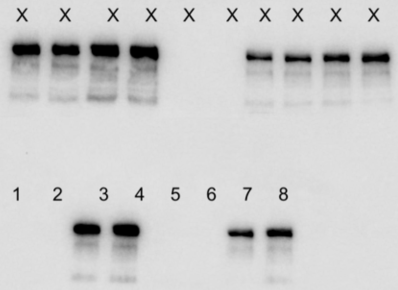

1: pQXCIP NT
2: pQXCIP Mock
3: pQXCIP Omicron MOI 0.1
4: pQXCIP Omicron MOI 1
5: pQC-BST2 NT
6: pQC-BST2 Mock
7: pQC-BST2 Omicron MOI 0.1
8: pQC-BST2 Omicron MOI 1

S2A, Omicron N

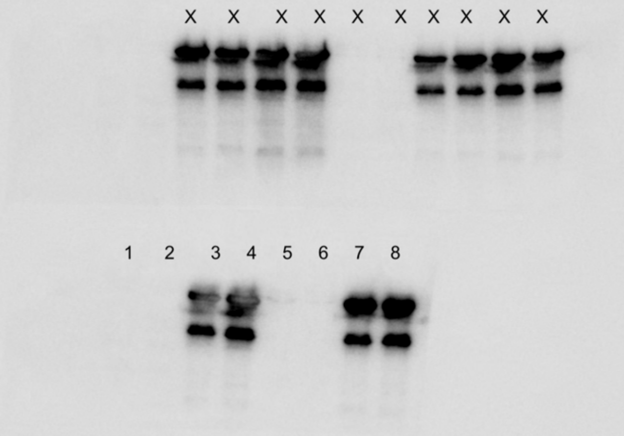

1: pQXCIP NT
2: pQXCIP Mock
3: pQXCIP Omicron MOI 0.1
4: pQXCIP Omicron MOI 1
5: pQC-BST2 NT
6: pQC-BST2 Mock
7: pQC-BST2 Omicron MOI 0.1
8: pQC-BST2 Omicron MOI 1

S2A, Omicron BST2

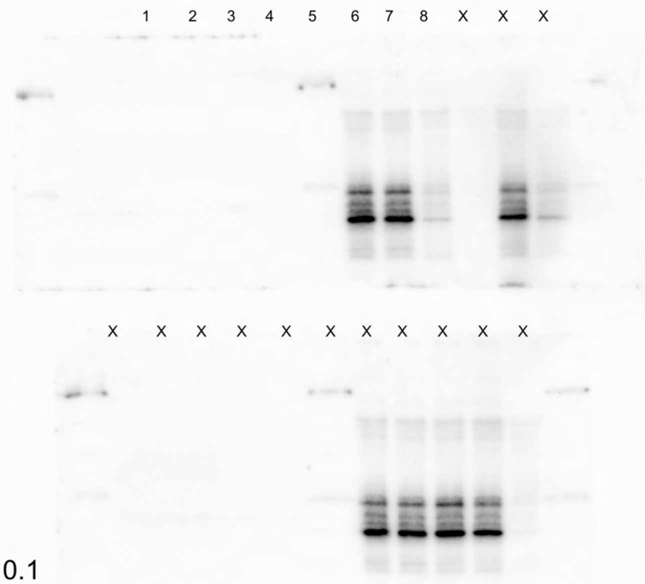

1: pQXCIP NT
2: pQXCIP Mock
3: pQXCIP Omicron MOI 0.1
4: pQXCIP Omicron MOI 1
5: pQC-BST2 NT
6: pQC-BST2 Mock
7: pQC-BST2 Omicron MOI 0.1
8: pQC-BST2 Omicron MOI 1

S2A, Omicron Actin

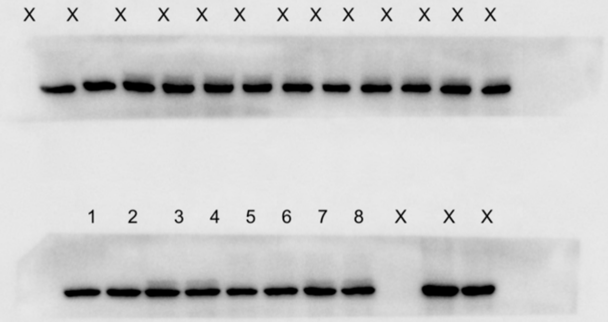

1: pQXCIP NT
2: pQXCIP Mock
3: pQXCIP Omicron MOI 0.1
4: pQXCIP Omicron MOI 1
5: pQC-BST2 NT
6: pQC-BST2 Mock
7: pQC-BST2 Omicron MOI 0.1
8: pQC-BST2 Omicron MOI 1

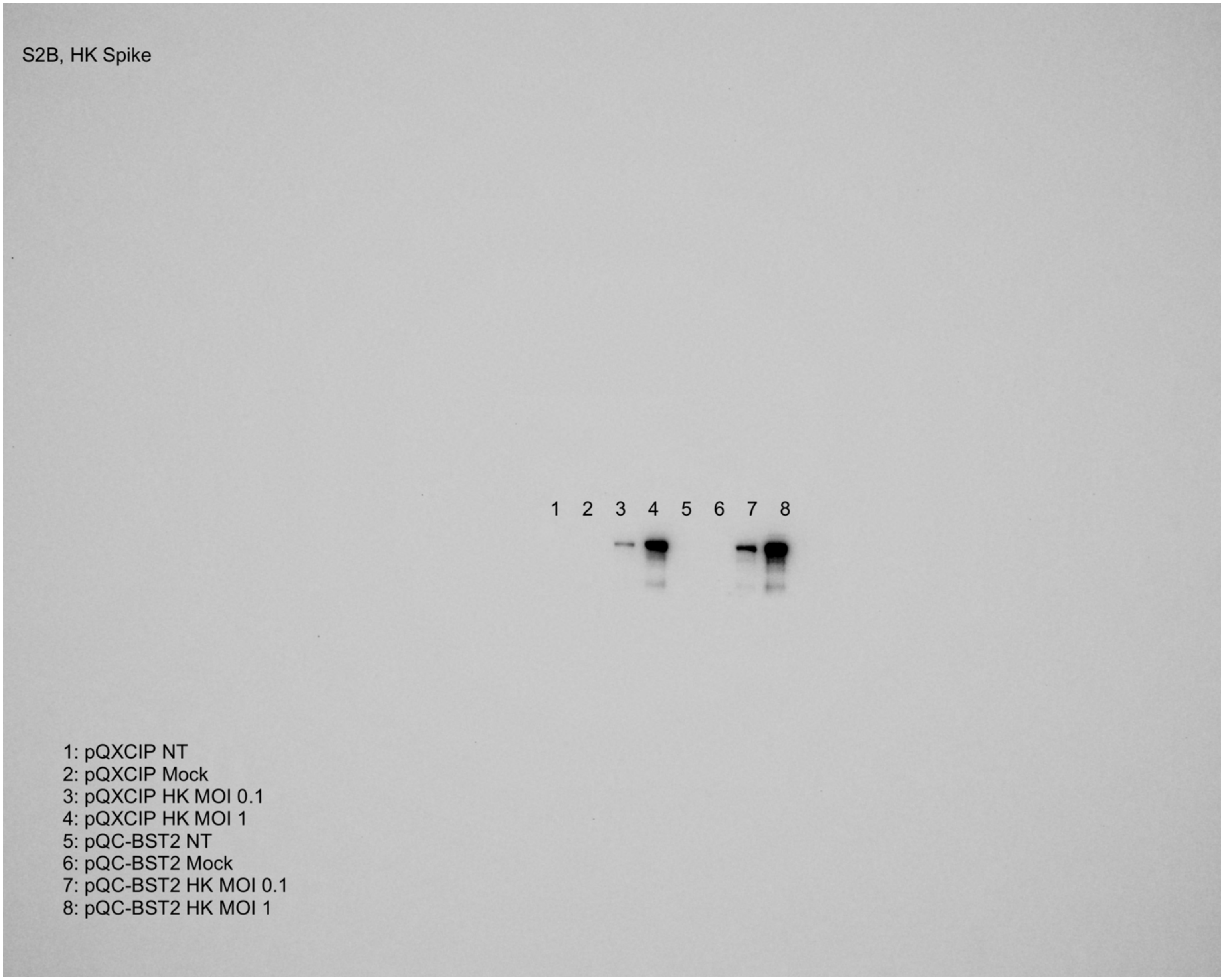
S2B, HK Spike
1 2 3 4 5 6 7 8
1: pQXCIP NT
2: pQXCIP Mock
3: pQXCIP HK MOI 0.1
4: pQXCIP HK MOI 1
5: pQC-BST2 NT
6: pQC-BST2 Mock
7: pQC-BST2 HK MOI 0.1
8: pQC-BST2 HK MOI 1

S2B, N **HK**

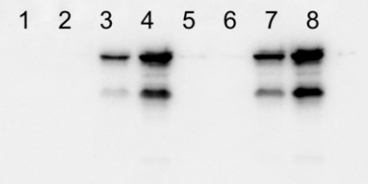

1: pQXCIP NT
2: pQXCIP Mock
3: pQXCIP HK MOI 0.1
4: pQXCIP HK MOI 1
5: pQC-BST2 NT
6: pQC-BST2 Mock
7: pQC-BST2 HK MOI 0.1
8: pQC-BST2 HK MOI 1

S2B, HK BST2

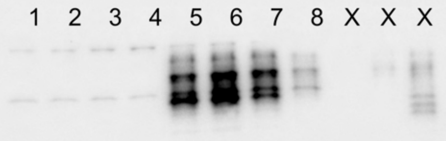

1: pQXCIP NT
2: pQXCIP Mock
3: pQXCIP HK MOI 0.1
4: pQXCIP HK MOI 1
5: pQC-BST2 NT
6: pQC-BST2 Mock
7: pQC-BST2 HK MOI 0.1
8: pQC-BST2 HK MOI 1

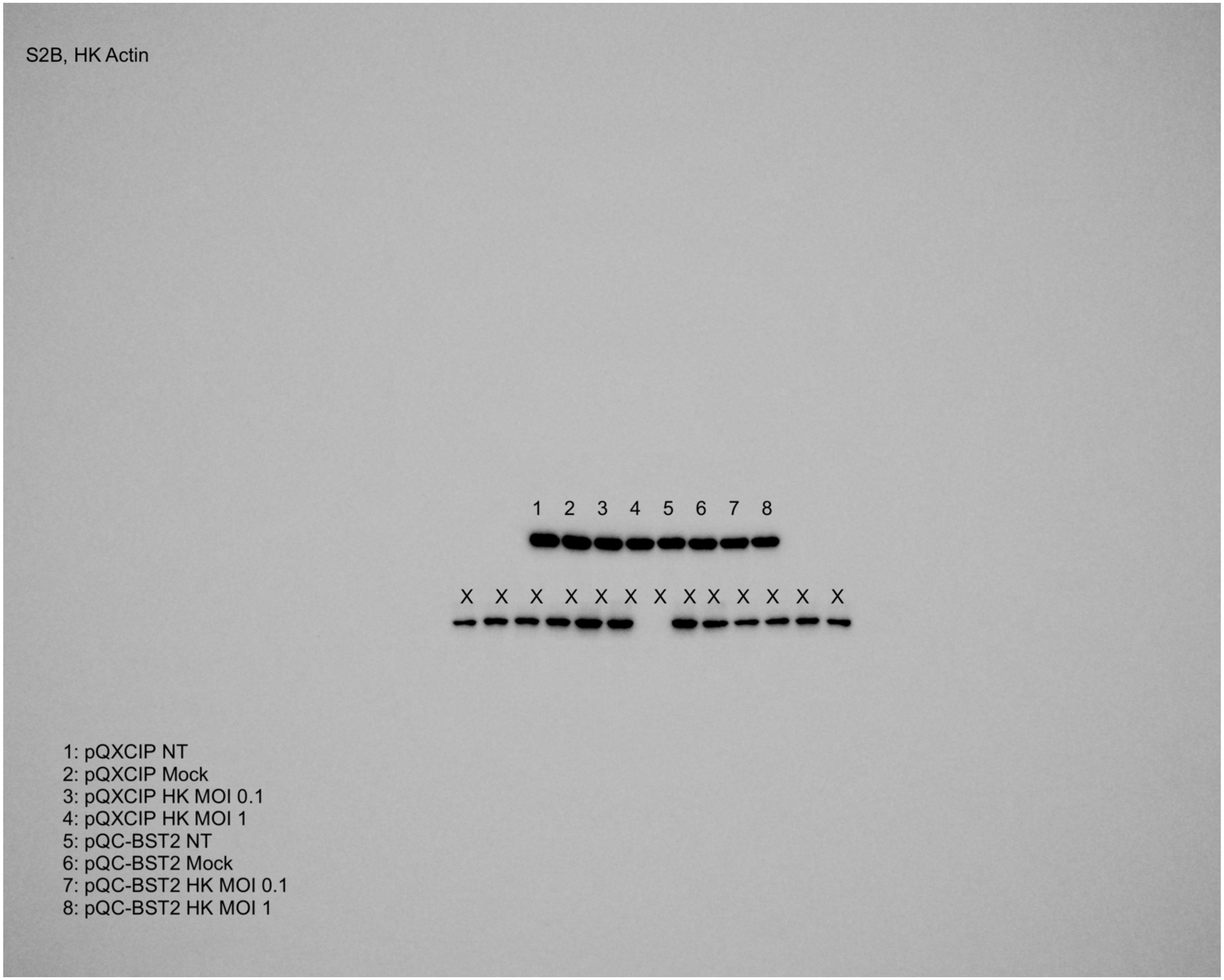
S2B, HK Actin
1 2 3 4 5 6 7 8
X X X X X X X X X X X X X
1: pQXCIP NT
2: pQXCIP Mock
3: pQXCIP HK MOI 0.1
4: pQXCIP HK MOI 1
5: pQC-BST2 NT
6: pQC-BST2 Mock
7: pQC-BST2 HK MOI 0.1
8: pQC-BST2 HK MOI 1

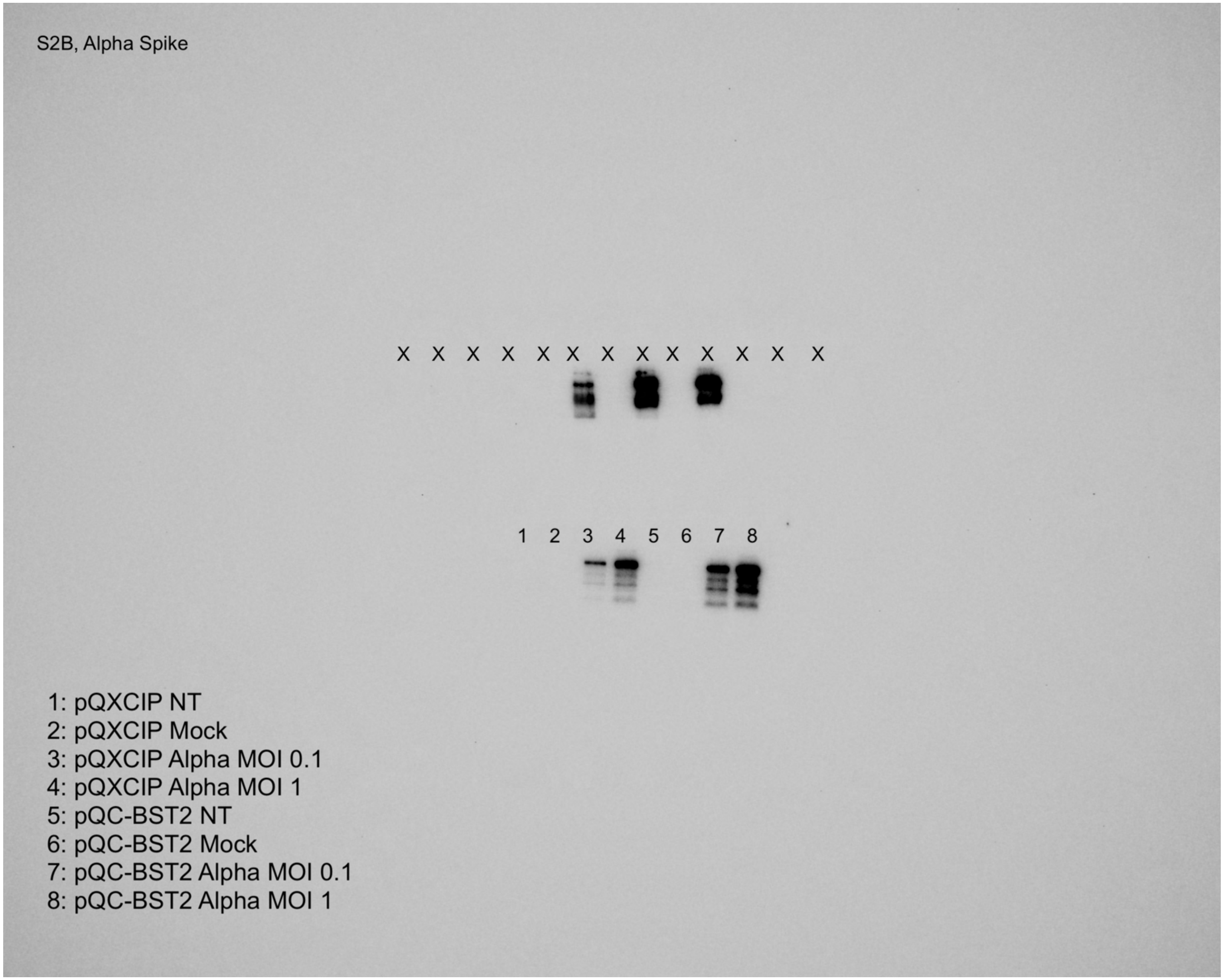
S2B, Alpha Spike
X X X X X X X X X X X X X
1 2 3 4 5 6 7 8
1: pQXCIP NT
2: pQXCIP Mock
3: pQXCIP Alpha MOI 0.1
4: pQXCIP Alpha MOI 1
5: pQC-BST2 NT
6: pQC-BST2 Mock
7: pQC-BST2 Alpha MOI 0.1
8: pQC-BST2 Alpha MOI 1

S2B, Alpha N

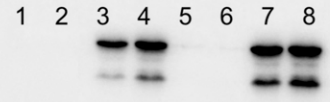

1: pQXCIP NT
2: pQXCIP Mock
3: pQXCIP Alpha MOI 0.1
4: pQXCIP Alpha MOI 1
5: pQC-BST2 NT
6: pQC-BST2 Mock
7: pQC-BST2 Alpha MOI 0.1
8: pQC-BST2 Alpha MOI 1

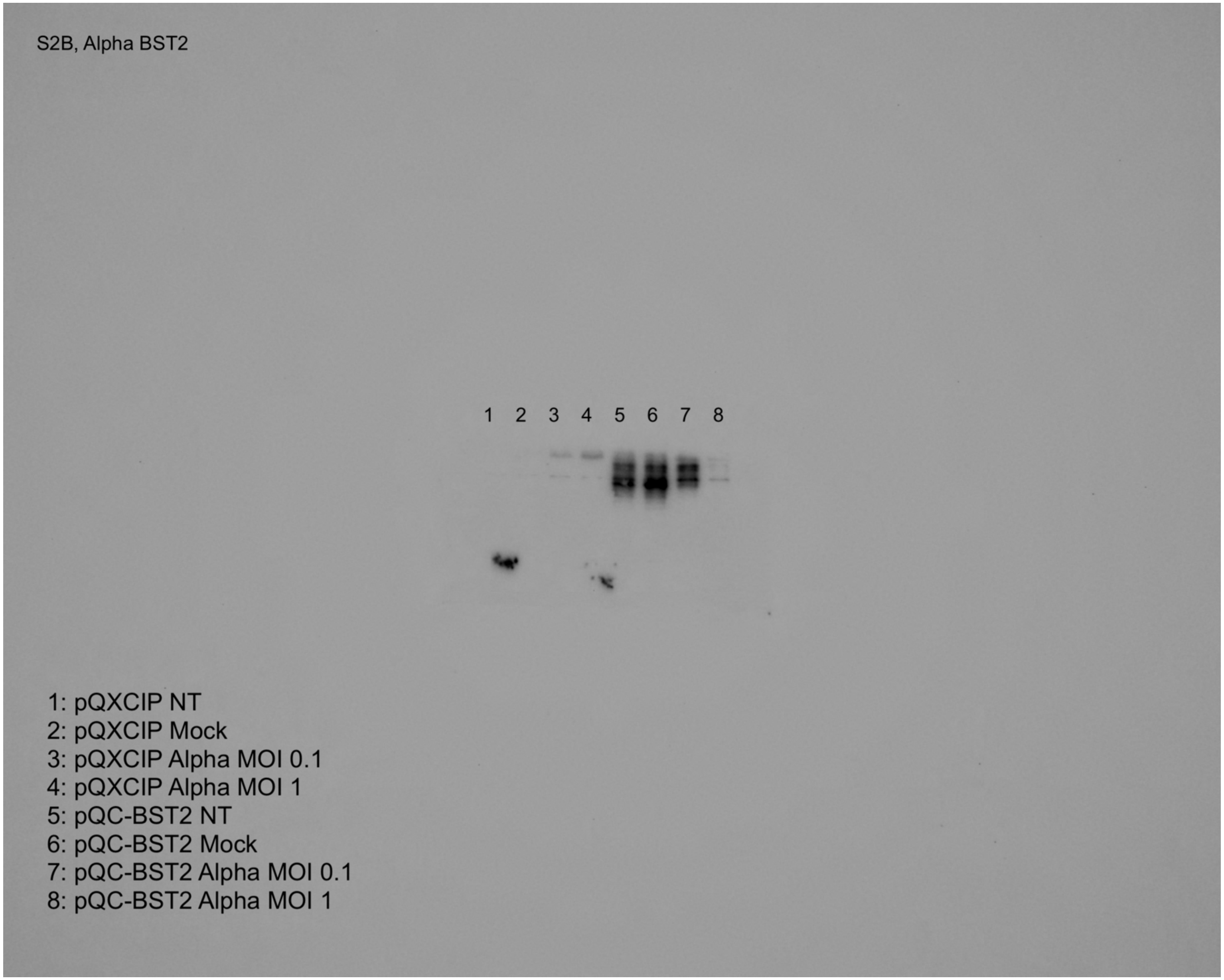
S2B, Alpha BST2
1 2 3 4 5 6 7 8
1: pQXCIP NT
2: pQXCIP Mock
3: pQXCIP Alpha MOI 0.1
4: pQXCIP Alpha MOI 1
5: pQC-BST2 NT
6: pQC-BST2 Mock
7: pQC-BST2 Alpha MOI 0.1
8: pQC-BST2 Alpha MOI 1

S2B, Alpha Actin

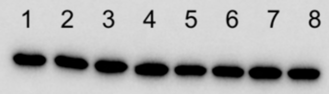

1: pQXCIP NT
2: pQXCIP Mock
3: pQXCIP Alpha MOI 0.1
4: pQXCIP Alpha MOI 1
5: pQC-BST2 NT
6: pQC-BST2 Mock
7: pQC-BST2 Alpha MOI 0.1
8: pQC-BST2 Alpha MOI 1

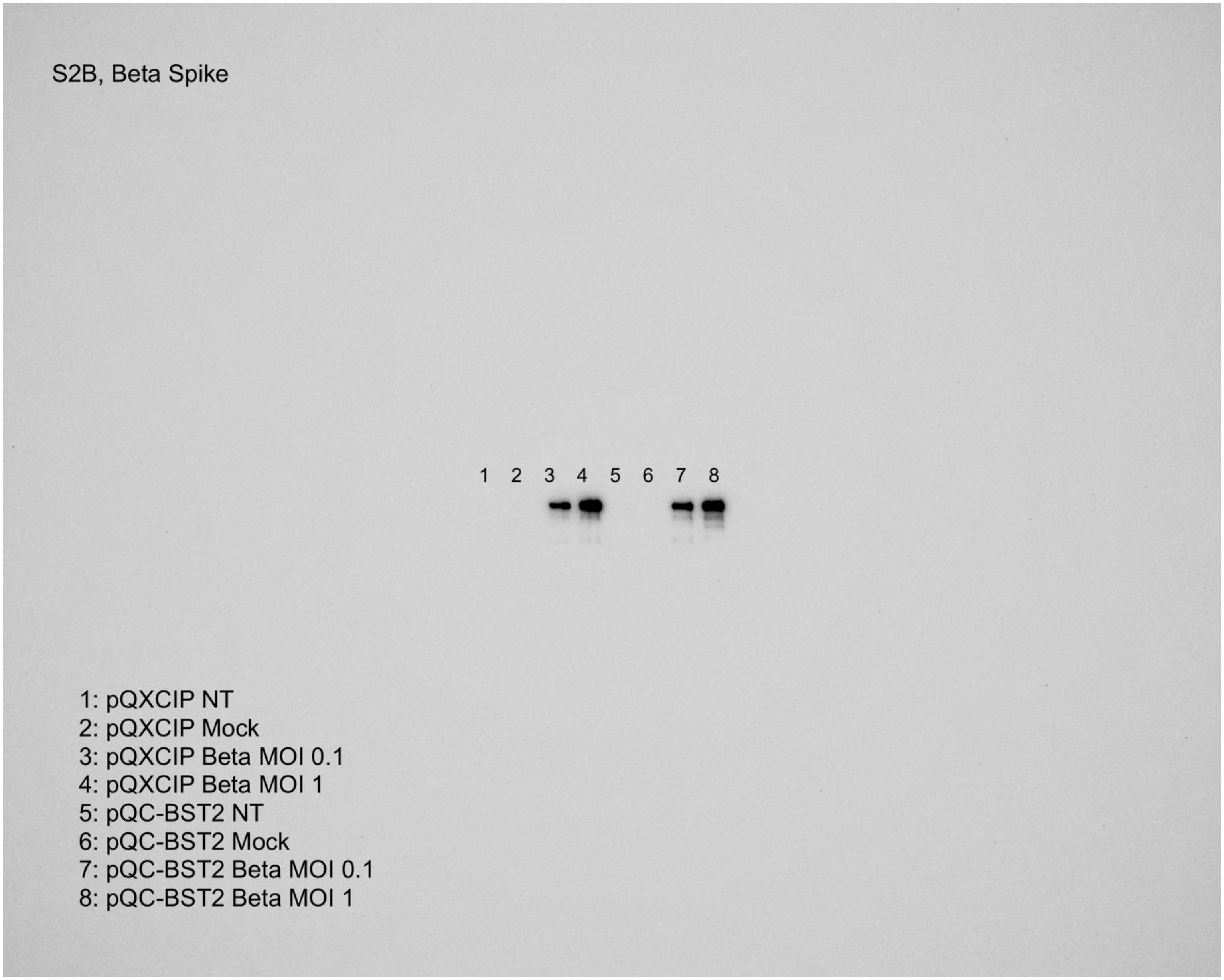
S2B, Beta Spike
1 2 3 4 5 6 7 8
1: pQXCIP NT
2: pQXCIP Mock
3: pQXCIP Beta MOI 0.1
4: pQXCIP Beta MOI 1
5: pQC-BST2 NT
6: pQC-BST2 Mock
7: pQC-BST2 Beta MOI 0.1
8: pQC-BST2 Beta MOI 1

S2B, Beta N

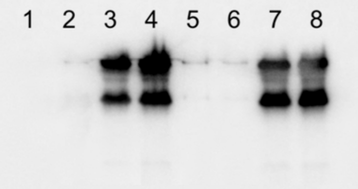

1: pQXCIP NT
2: pQXCIP Mock
3: pQXCIP Beta MOI 0.1
4: pQXCIP Beta MOI 1
5: pQC-BST2 NT
6: pQC-BST2 Mock
7: pQC-BST2 Beta MOI 0.1
8: pQC-BST2 Beta MOI 1

S2B, Beta BST2

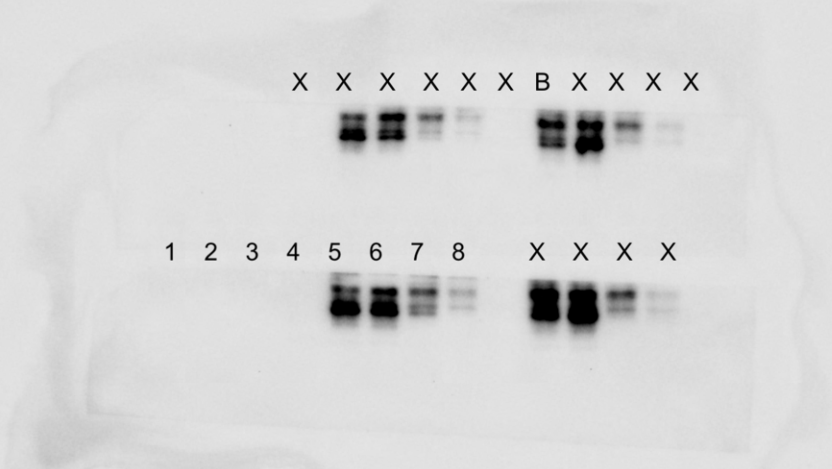

1: pQXCIP NT
2: pQXCIP Mock
3: pQXCIP Beta MOI 0.1
4: pQXCIP Beta MOI 1
5: pQC-BST2 NT
6: pQC-BST2 Mock
7: pQC-BST2 Beta MOI 0.1
8: pQC-BST2 Beta MOI 1
X: replicates

S2B, Beta Actin

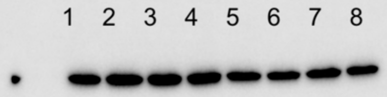

1: pQXCIP NT
2: pQXCIP Mock
3: pQXCIP Beta MOI 0.1
4: pQXCIP Beta MOI 1
5: pQC-BST2 NT
6: pQC-BST2 Mock
7: pQC-BST2 Beta MOI 0.1
8: pQC-BST2 Beta MOI 1

S2B, Delta Spike

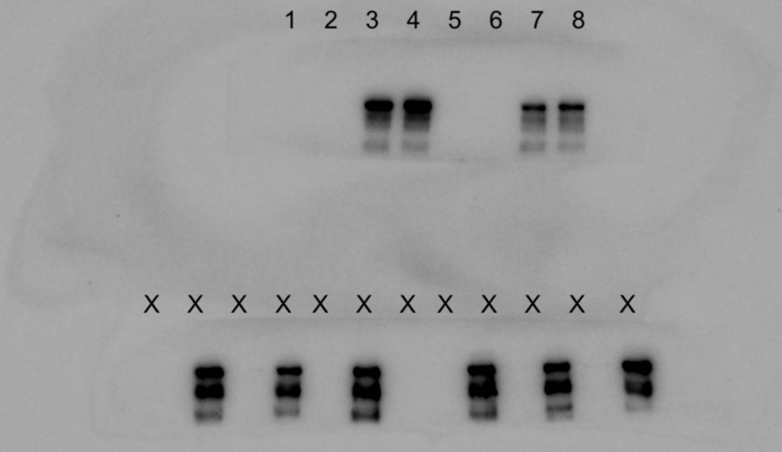

1: pQXCIP NT
2: pQXCIP Mock
3: pQXCIP Delta MOI 0.1
4: pQXCIP Delta MOI 1
5: pQC-BST2 NT
6: pQC-BST2 Mock
7: pQC-BST2 Delta MOI 0.1
8: pQC-BST2 Delta MOI 1

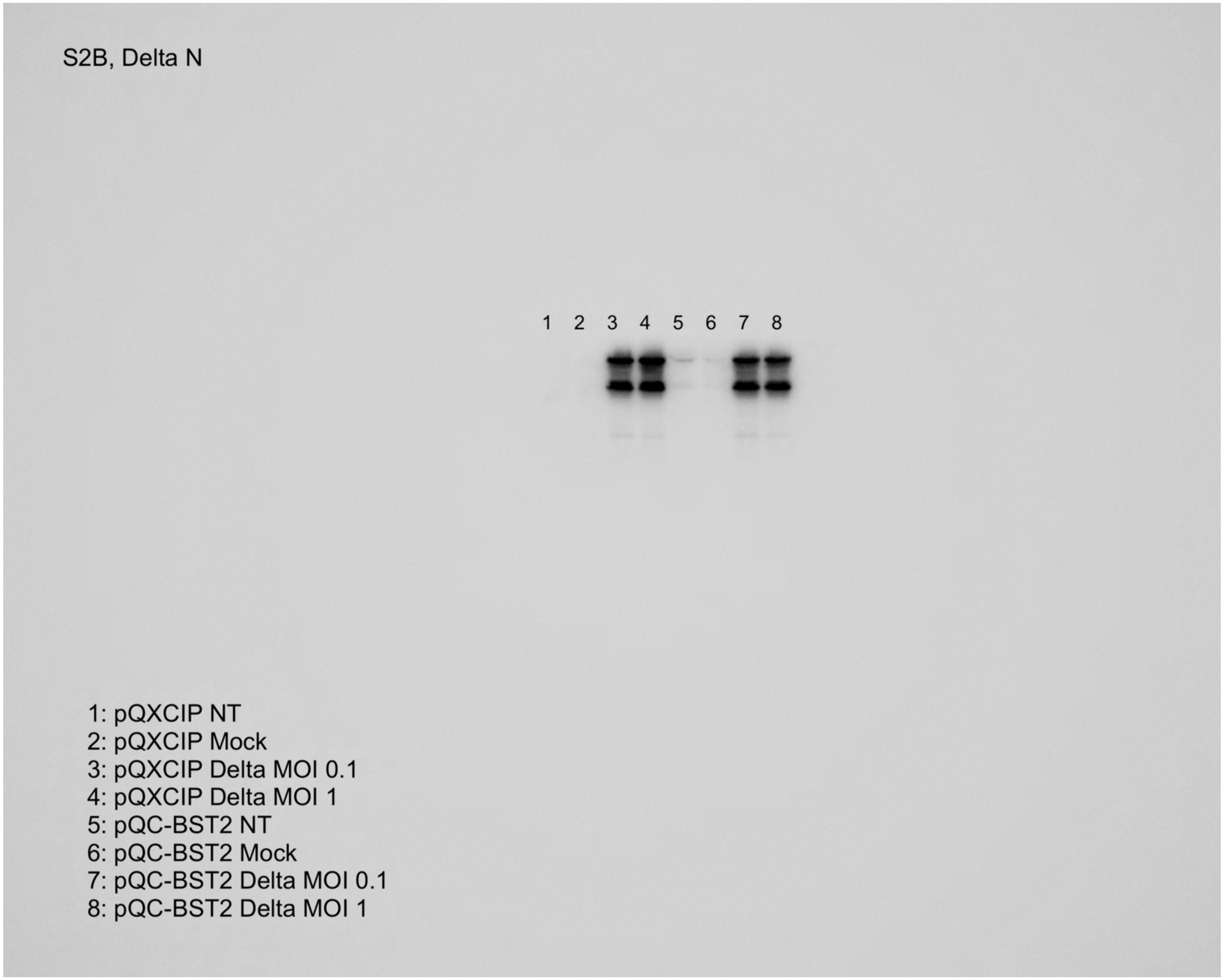
S2B, Delta N
1 2 3 4 5 6 7 8
1: pQXCIP NT
2: pQXCIP Mock
3: pQXCIP Delta MOI 0.1
4: pQXCIP Delta MOI 1
5: pQC-BST2 NT
6: pQC-BST2 Mock
7: pQC-BST2 Delta MOI 0.1
8: pQC-BST2 Delta MOI 1

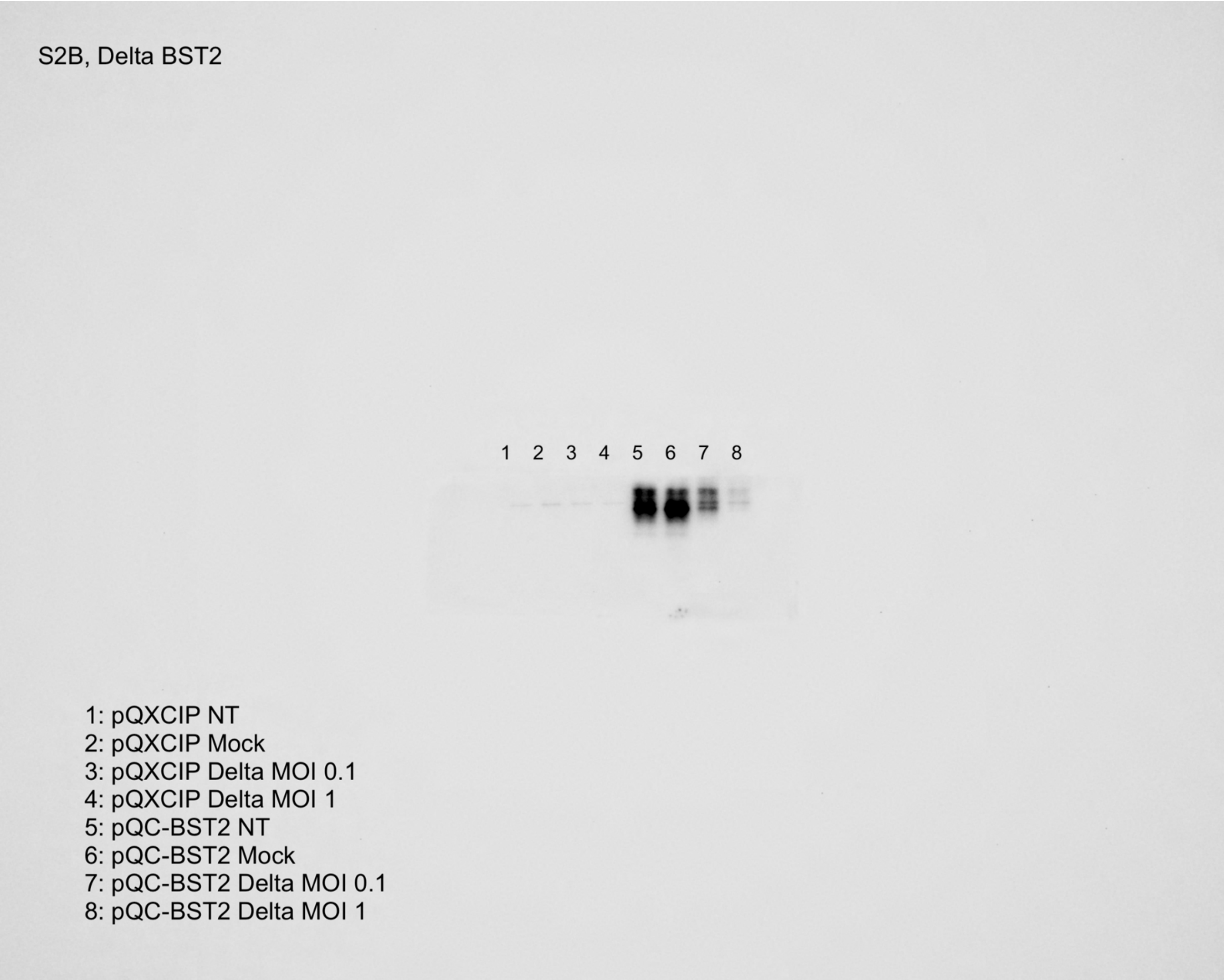
S2B, Delta BST2
1 2 3 4 5 6 7 8
1: pQXCIP NT
2: pQXCIP Mock
3: pQXCIP Delta MOI 0.1
4: pQXCIP Delta MOI 1
5: pQC-BST2 NT
6: pQC-BST2 Mock
7: pQC-BST2 Delta MOI 0.1
8: pQC-BST2 Delta MOI 1

S2B, Delta ACTIN

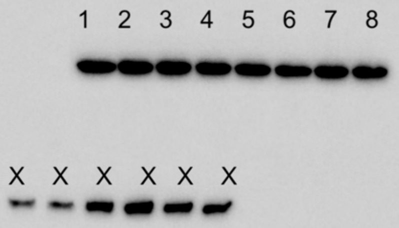

1: pQXCIP NT
2: pQXCIP Mock
3: pQXCIP Delta MOI 0.1
4: pQXCIP Delta MOI 1
5: pQC-BST2 NT
6: pQC-BST2 Mock
7: pQC-BST2 Delta MOI 0.1
8: pQC-BST2 Delta MOI 1

S2B, Omicron Spike

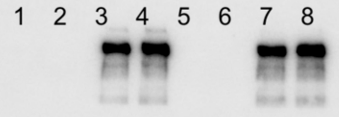

1: pQXCIP NT
2: pQXCIP Mock
3: pQXCIP Omicron MOI 0.1
4: pQXCIP Omicron MOI 1
5: pQC-BST2 NT
6: pQC-BST2 Mock
7: pQC-BST2 Omicron MOI 0.1
8: pQC-BST2 Omicron MOI 1

S2B, Omicron N

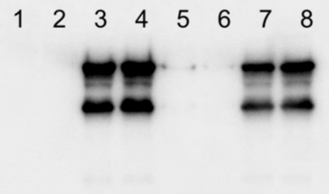

1: pQXCIP NT
2: pQXCIP Mock
3: pQXCIP Omicron MOI 0.1
4: pQXCIP Omicron MOI 1
5: pQC-BST2 NT
6: pQC-BST2 Mock
7: pQC-BST2 Omicron MOI 0.1
8: pQC-BST2 Omicron MOI 1

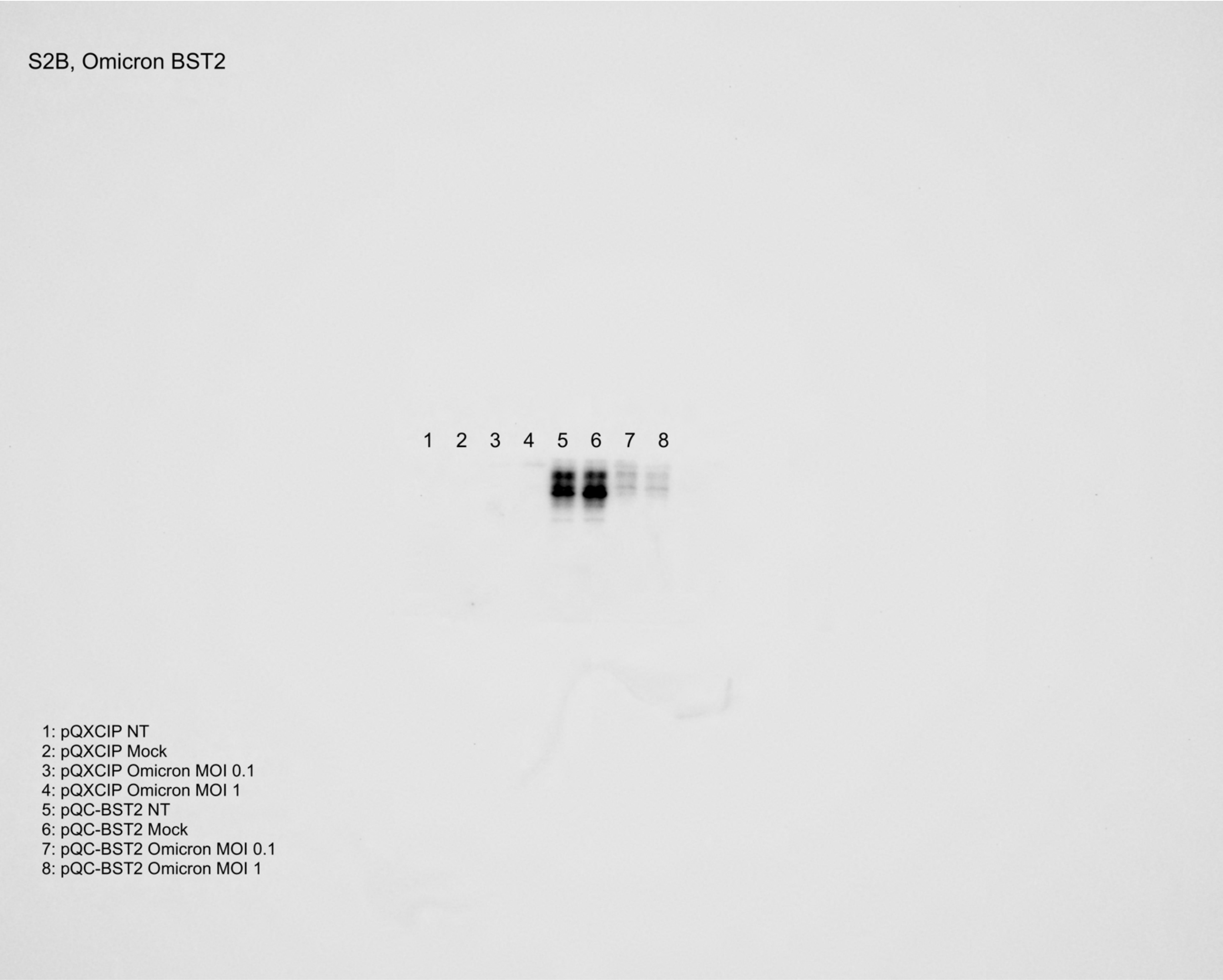
S2B, Omicron BST2
1 2 3 4 5 6 7 8
1: pQXCIP NT
2: pQXCIP Mock
3: pQXCIP Omicron MOI 0.1
4: pQXCIP Omicron MOI 1
5: pQC-BST2 NT
6: pQC-BST2 Mock
7: pQC-BST2 Omicron MOI 0.1
8: pQC-BST2 Omicron MOI 1

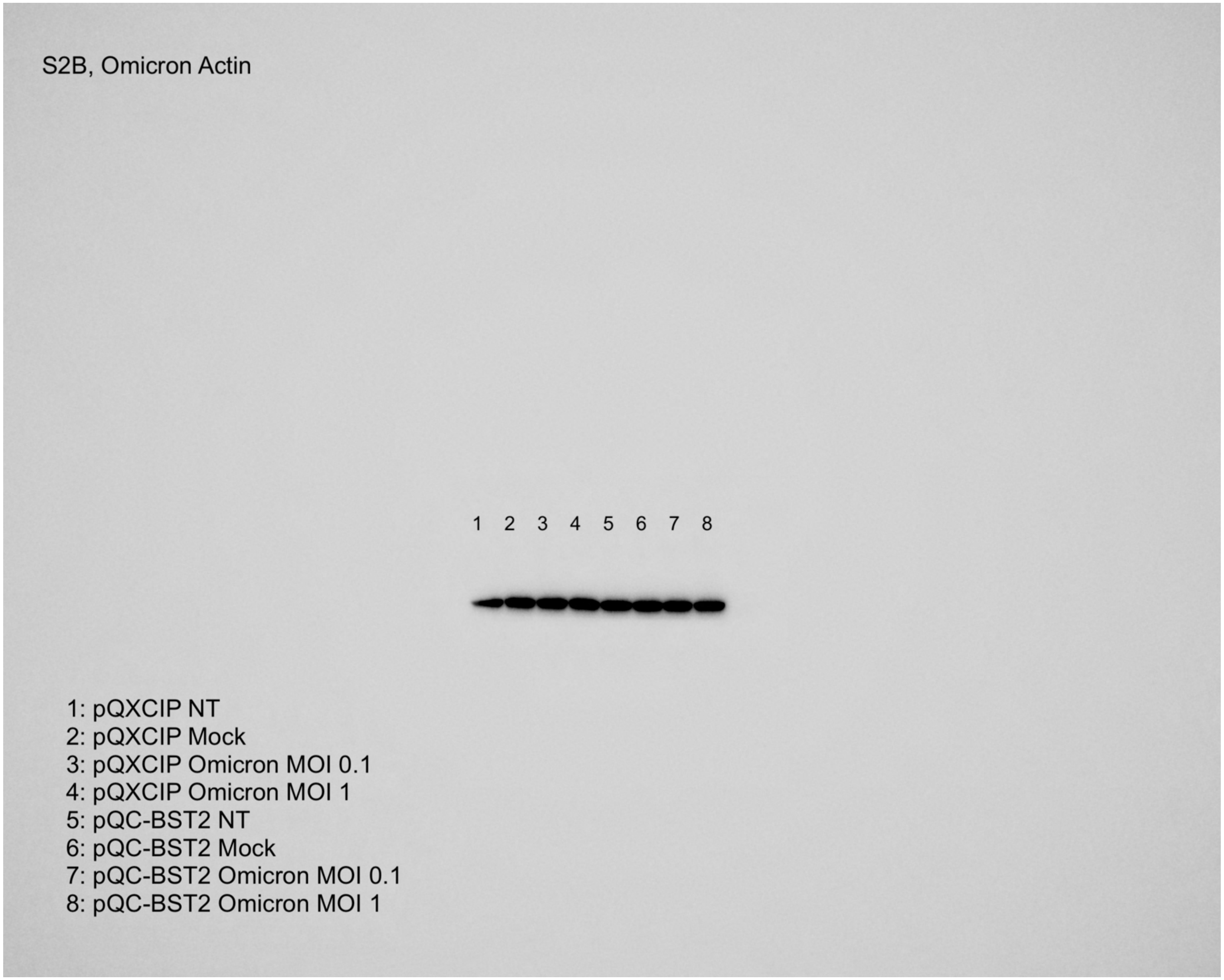
S2B, Omicron Actin
1 2 3 4 5 6 7 8
1: pQXCIP NT
2: pQXCIP Mock
3: pQXCIP Omicron MOI 0.1
4: pQXCIP Omicron MOI 1
5: pQC-BST2 NT
6: pQC-BST2 Mock
7: pQC-BST2 Omicron MOI 0.1
8: pQC-BST2 Omicron MOI 1

S5A, Spike

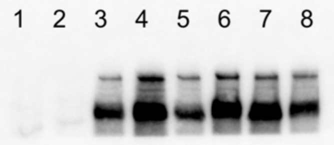

1: GST-HA
2: Vpu
3: Wuhan S
4: Alpha S
5: Beta S
6: Gamma S
7: Delta S
8: Omicron S

S5A, BST2

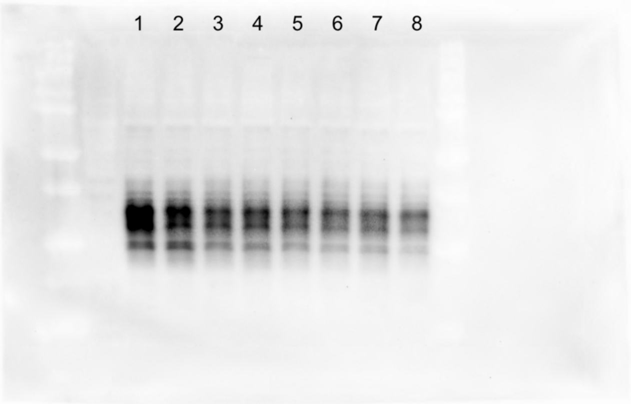

1: GST-HA
2: Vpu
3: Wuhan S
4: Alpha S
5: Beta S
6: Gamma S
7: Delta S
8: Omicron S

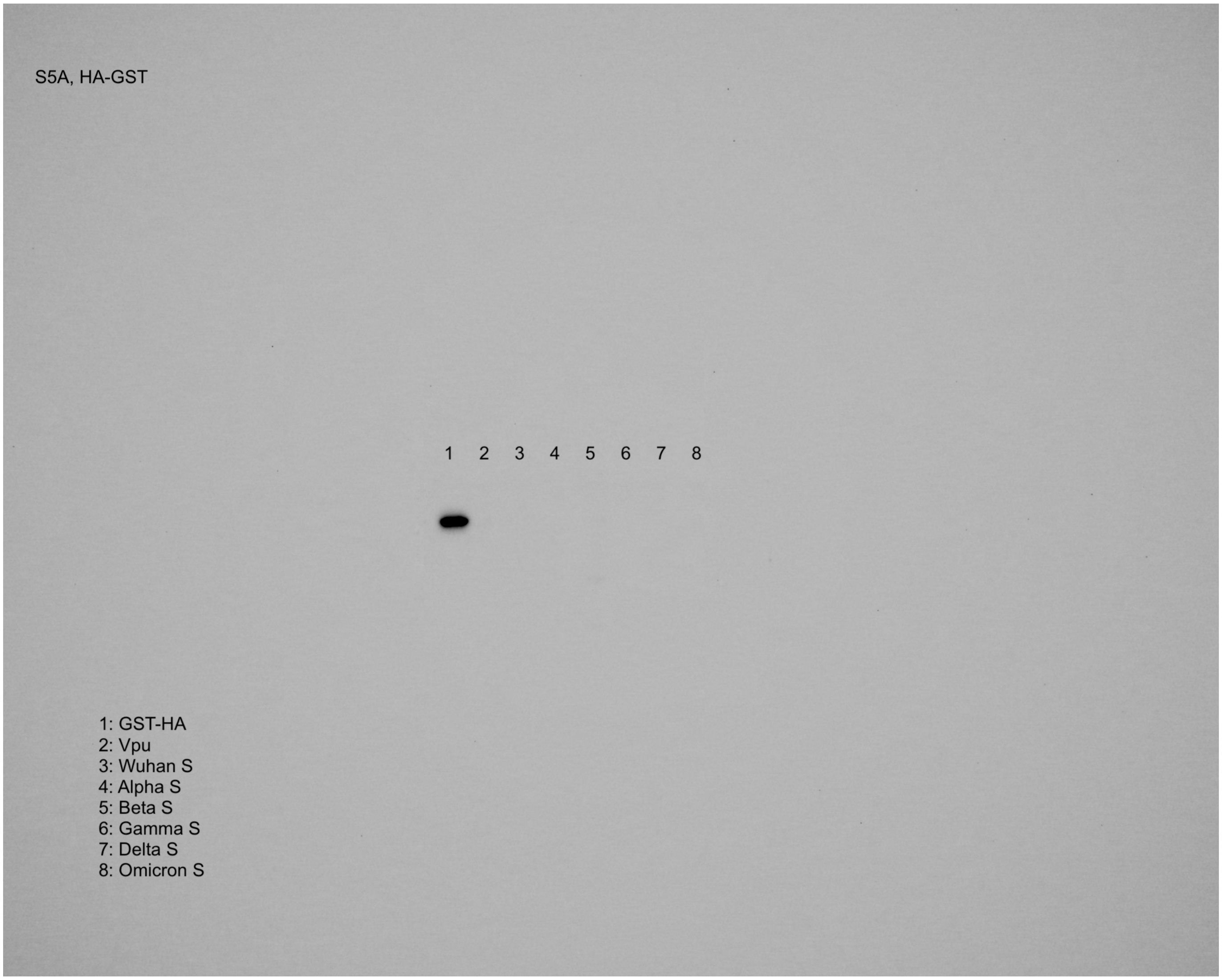
S5A, HA-GST
1 2 3 4 5 6 7 8
1: GST-HA
2: Vpu
3: Wuhan S
4: Alpha S
5: Beta S
6: Gamma S
7: Delta S
8: Omicron S

S5A, Vpu

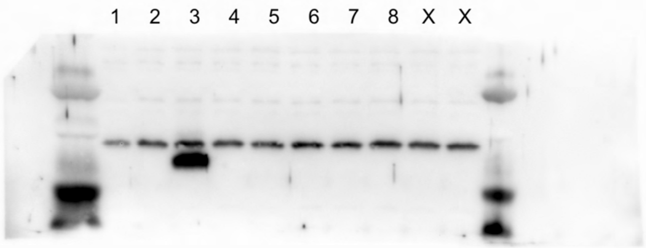

1: GST-HA
2: Vpu
3: Wuhan S
4: Alpha S
5: Beta S
6: Gamma S
7: Delta S
8: Omicron S

S5A, Actin

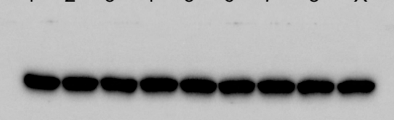

1: GST-HA
2: Vpu
3: Wuhan S
4: Alpha S
5: Beta S
6: Gamma S
7: Delta S
8: Omicron S

S6B, IP Spike

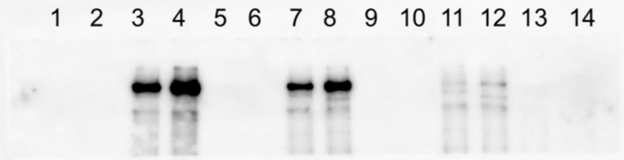

1: GFP + BST2
2: VPU + BST2
3: Swu + BST2
4: Somi + BST2
5: GFP + deltaCT
6: VPU + deltaCT
7: Swu + deltaCT
8: Somi + deltaCT
9: GFP + deltaCT-TM
10: VPU + deltaCT-TM
11: Swu + deltaCT-TM
12: Somi + deltaCT-TM
13: Beads
14: IgG

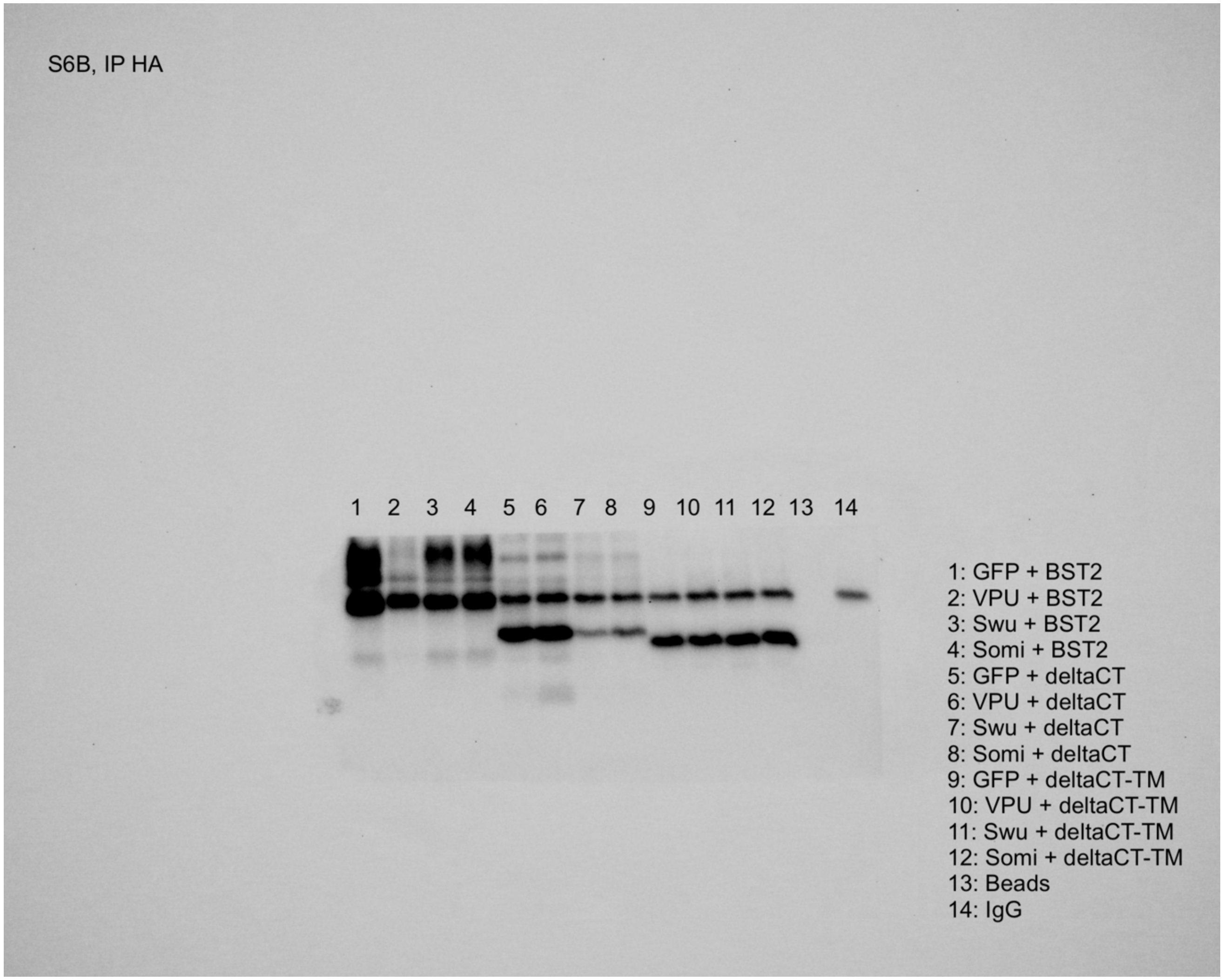
S6B, IP HA
1 2 3 4 5 6 7 8 9 10 11 12 13 14
1: GFP + BST2
2: VPU + BST2
3: Swu + BST2
4: Somi + BST2
5: GFP + deltaCT
6: VPU + deltaCT
7: Swu + deltaCT
8: Somi + deltaCT
9: GFP + deltaCT-TM
10: VPU + deltaCT-TM
11: Swu + deltaCT-TM
12: Somi + deltaCT-TM
13: Beads
14: IgG

S6B, IP GFP

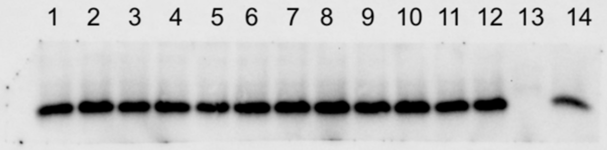

1: GFP + BST2
2: VPU + BST2
3: Swu + BST2
4: Somi + BST2
5: GFP + deltaCT
6: VPU + deltaCT
7: Swu + deltaCT
8: Somi + deltaCT
9: GFP + deltaCT-TM
10: VPU + deltaCT-TM
11: Swu + deltaCT-TM
12: Somi + deltaCT-TM
13: Beads
14: IgG

S6B, IP Vpu

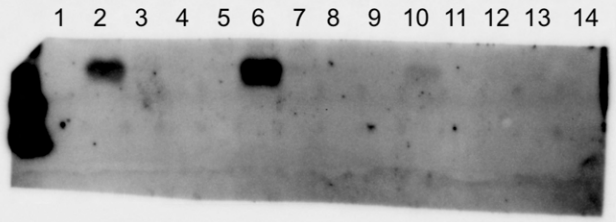

1: GFP + BST2
2: VPU + BST2
3: Swu + BST2
4: Somi + BST2
5: GFP + deltaCT
6: VPU + deltaCT
7: Swu + deltaCT
8: Somi + deltaCT
9: GFP + deltaCT-TM
10: VPU + deltaCT-TM
11: Swu + deltaCT-TM
12: Somi + deltaCT-TM
13: Beads
14: IgG

S6B, WCL Spike

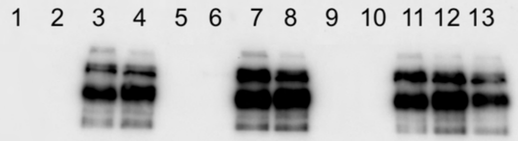

1: GFP + BST2
2: VPU + BST2
3: Swu + BST2
4: Somi + BST2
5: GFP + deltaCT
6: VPU + deltaCT
7: Swu + deltaCT
8: Somi + deltaCT
9: GFP + deltaCT-TM
10: VPU + deltaCT-TM
11: Swu + deltaCT-TM
12: Somi + deltaCT-TM
13: Beads

S6B, WCL BST2

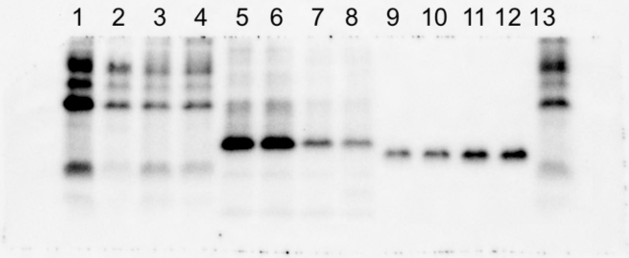

1: GFP + BST2
2: VPU + BST2
3: Swu + BST2
4: Somi + BST2
5: GFP + deltaCT
6: VPU + deltaCT
7: Swu + deltaCT
8: Somi + deltaCT
9: GFP + deltaCT-TM
10: VPU + deltaCT-TM
11: Swu + deltaCT-TM
12: Somi + deltaCT-TM
13: Beads

S6B, WCL GFP

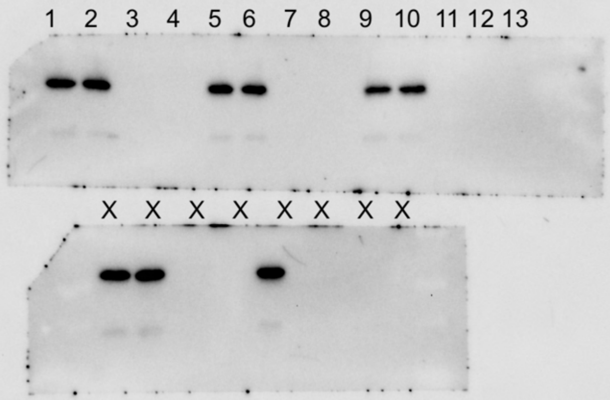

1: GFP + BST2
2: VPU + BST2
3: Swu + BST2
4: Somi + BST2
5: GFP + deltaCT
6: VPU + deltaCT
7: Swu + deltaCT
8: Somi + deltaCT
9: GFP + deltaCT-TM
10: VPU + deltaCT-TM
11: Swu + deltaCT-TM
12: Somi + deltaCT-TM
13: Beads

S6B, WCL VPU

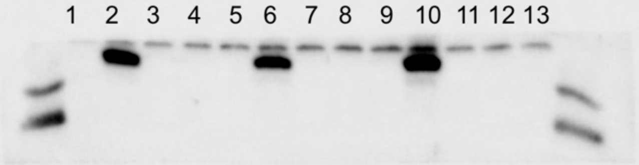

1: GFP + BST2
2: VPU + BST2
3: Swu + BST2
4: Somi + BST2
5: GFP + deltaCT
6: VPU + deltaCT
7: Swu + deltaCT
8: Somi + deltaCT
9: GFP + deltaCT-TM
10: VPU + deltaCT-TM
11: Swu + deltaCT-TM
12: Somi + deltaCT-TM
13: Beads

S6B, WCL Actin

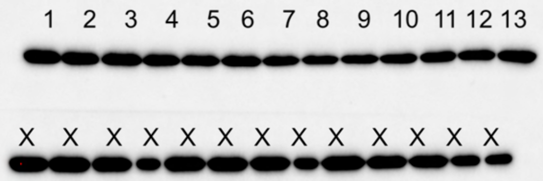

1: GFP + BST2
2: VPU + BST2
3: Swu + BST2
4: Somi + BST2
5: GFP + deltaCT
6: VPU + deltaCT
7: Swu + deltaCT
8: Somi + deltaCT
9: GFP + deltaCT-TM
10: VPU + deltaCT-TM
11: Swu + deltaCT-TM
12: Somi + deltaCT-TM
13: Beads

S6C, IP Spike

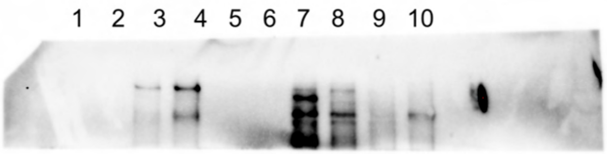

1: GFP + BST2
2: VPU + BST2
3: Swu + BST2
4: Somi + BST2
5: GFP + TfR-TM
6: VPU + TfR-TM
7: Swu + TfR-TM
8: Somi + TfR-TM
9: Beads
10: IgG

S6C, IP HA

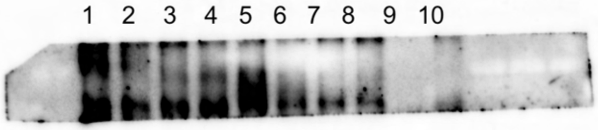

1: GFP + BST2
2: VPU + BST2
3: Swu + BST2
4: Somi + BST2
5: GFP + TfR-TM
6: VPU + TfR-TM
7: Swu + TfR-TM
8: Somi + TfR-TM
9: Beads
10: IgG

S6C, IP GFP

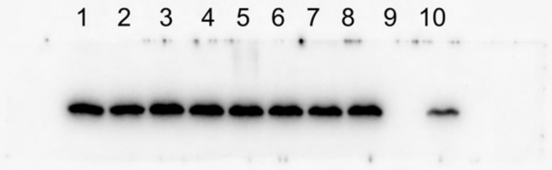

1: GFP + BST2
2: VPU + BST2
3: Swu + BST2
4: Somi + BST2
5: GFP + TfR-TM
6: VPU + TfR-TM
7: Swu + TfR-TM
8: Somi + TfR-TM
9: Beads
10: IgG

S6C, IP VPU

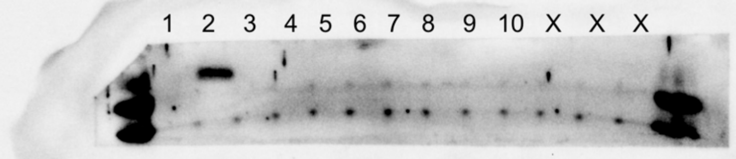

1: GFP + BST2
2: VPU + BST2
3: Swu + BST2
4: Somi + BST2
5: GFP + TfR-TM
6: VPU + TfR-TM
7: Swu + TfR-TM
8: Somi + TfR-TM
9: Beads
10: IgG

S6C, WCL Spike

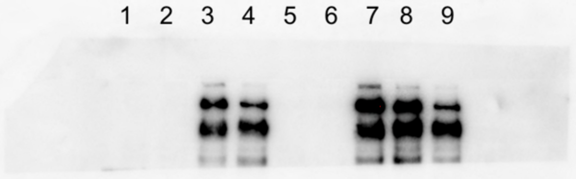

1: GFP + BST2
2: VPU + BST2
3: Swu + BST2
4: Somi + BST2
5: GFP + TfR-TM
6: VPU + TfR-TM
7: Swu + TfR-TM
8: Somi + TfR-TM
9: Beads

S6C, WCL HA

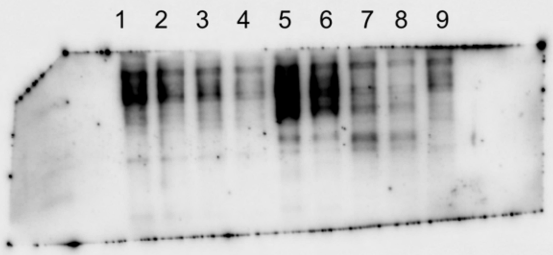

1: GFP + BST2
2: VPU + BST2
3: Swu + BST2
4: Somi + BST2
5: GFP + TfR-TM
6: VPU + TfR-TM
7: Swu + TfR-TM
8: Somi + TfR-TM
9: Beads

S6C, WCL GFP

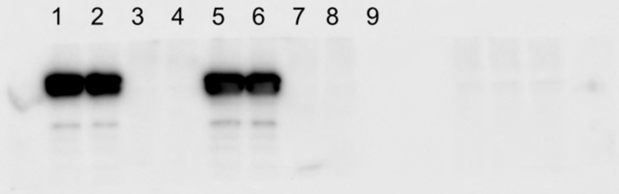

1: GFP + BST2
2: VPU + BST2
3: Swu + BST2
4: Somi + BST2
5: GFP + TfR-TM
6: VPU + TfR-TM
7: Swu + TfR-TM
8: Somi + TfR-TM
9: Beads

S6C, WCL VPU

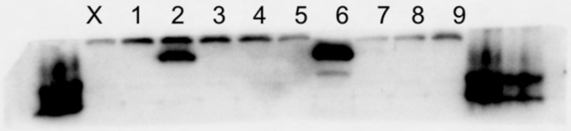

1: GFP + BST2
2: VPU + BST2
3: Swu + BST2
4: Somi + BST2
5: GFP + TfR-TM
6: VPU + TfR-TM
7: Swu + TfR-TM
8: Somi + TfR-TM
9: Beads

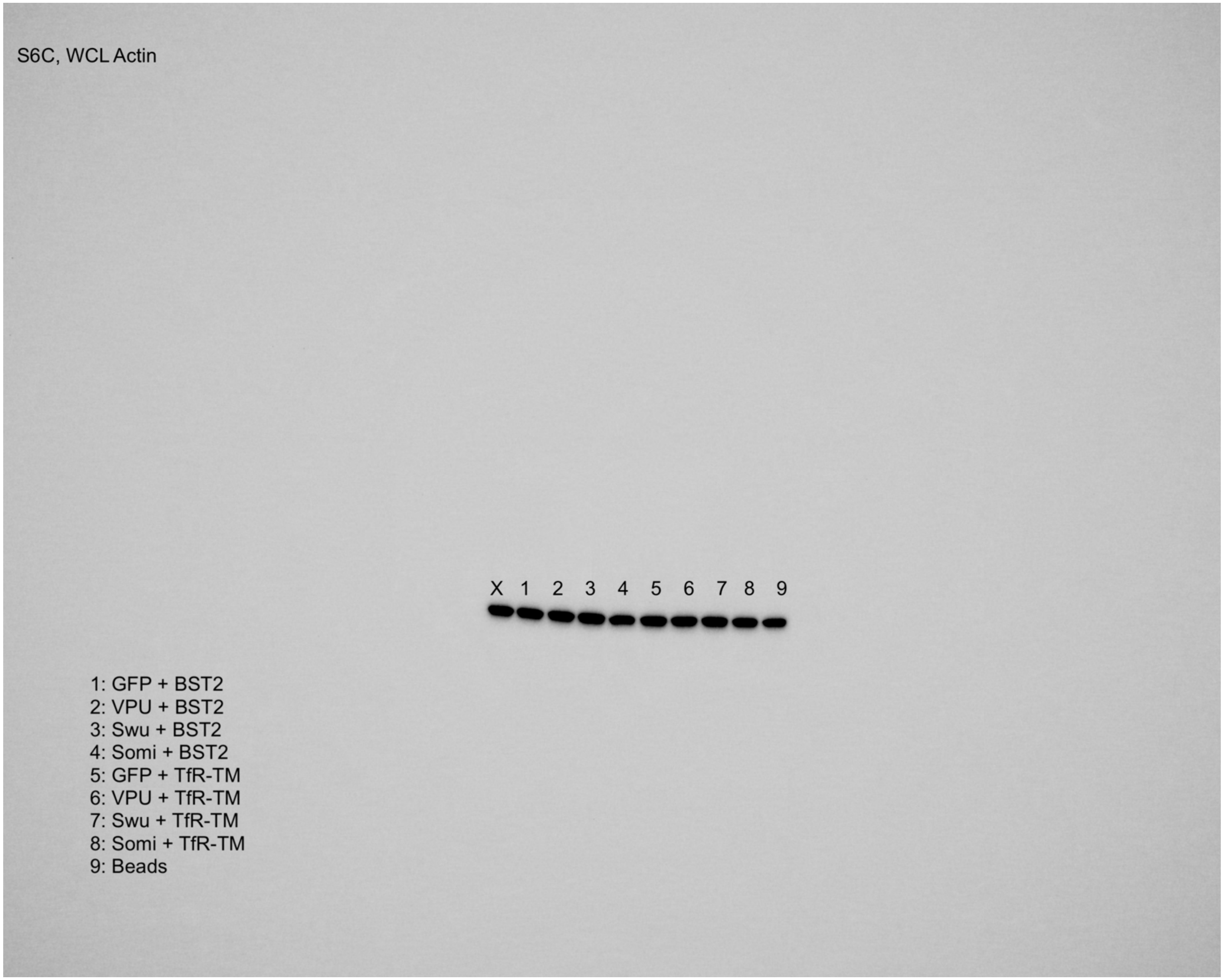
S6C, WCL Actin
X 1 2 3 4 5 6 7 8 9
1: GFP + BST2
2: VPU + BST2
3: Swu + BST2
4: Somi + BST2
5: GFP + TfR-TM
6: VPU + TfR-TM
7: Swu + TfR-TM
8: Somi + TfR-TM
9: Beads

S6D, IP Spike

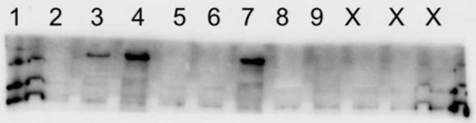

1: GFP + BST2 (flow over with ladder)
2: VPU + BST2
3: Swu + BST2
4: Somi + BST2
5: GFP + deltaCC
6: Swu + deltaCC
7: Somi + deltaCC
8: Beads
9: IgG

S6D, IP BST2

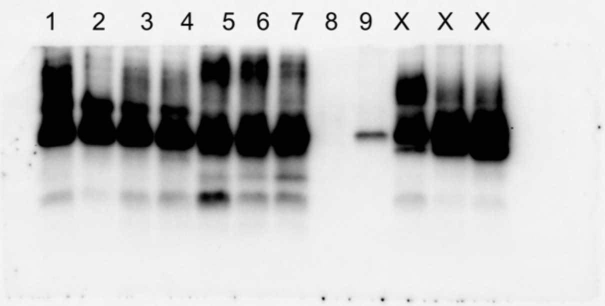

1: GFP + BST2
2: VPU + BST2
3: Swu + BST2
4: Somi + BST2
5: GFP + deltaCC
6: Swu + deltaCC
7: Somi + deltaCC
8: Beads
9: IgG

S6D, IP GFP

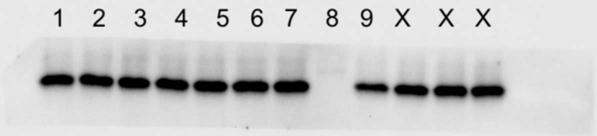

1: GFP + BST2
2: VPU + BST2
3: Swu + BST2
4: Somi + BST2
5: GFP + deltaCC
6: Swu + deltaCC
7: Somi + deltaCC
8: Beads
9: IgG

S6D, IP VPU

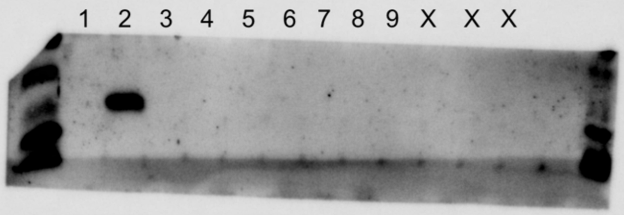

1: GFP + BST2
2: VPU + BST2
3: Swu + BST2
4: Somi + BST2
5: GFP + deltaCC
6: Swu + deltaCC
7: Somi + deltaCC
8: Beads
9: IgG

S6D, WCL Spike

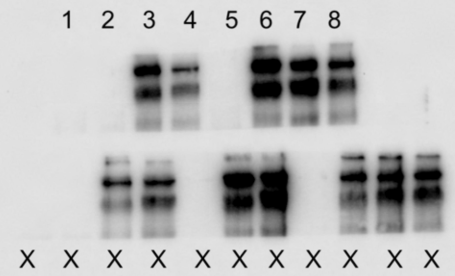

1: GFP + BST2
2: VPU + BST2
3: Swu + BST2
4: Somi + BST2
5: GFP + deltaCC
6: Swu + deltaCC
7: Somi + deltaCC
8: Beads

S6D, WCL HA

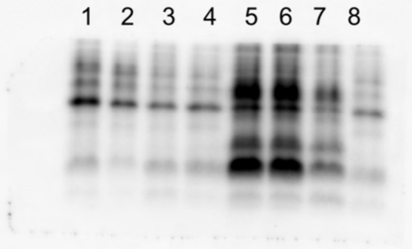

1: GFP + BST2
2: VPU + BST2
3: Swu + BST2
4: Somi + BST2
5: GFP + deltaCC
6: Swu + deltaCC
7: Somi + deltaCC
8: Beads

S6D, WCL GFP

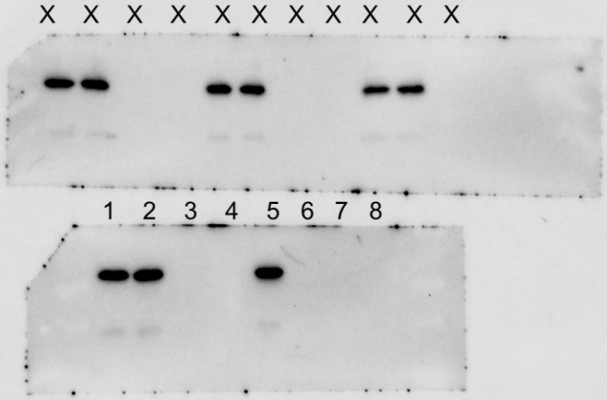

1: GFP + BST2
2: VPU + BST2
3: Swu + BST2
4: Somi + BST2
5: GFP + deltaCC
6: Swu + deltaCC
7: Somi + deltaCC
8: Beads

S6D, WCL Vpu

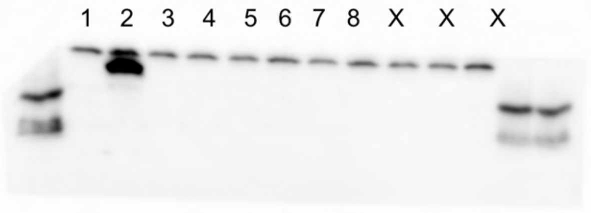

1: GFP + BST2
2: VPU + BST2
3: Swu + BST2
4: Somi + BST2
5: GFP + deltaCC
6: Swu + deltaCC
7: Somi + deltaCC
8: Beads

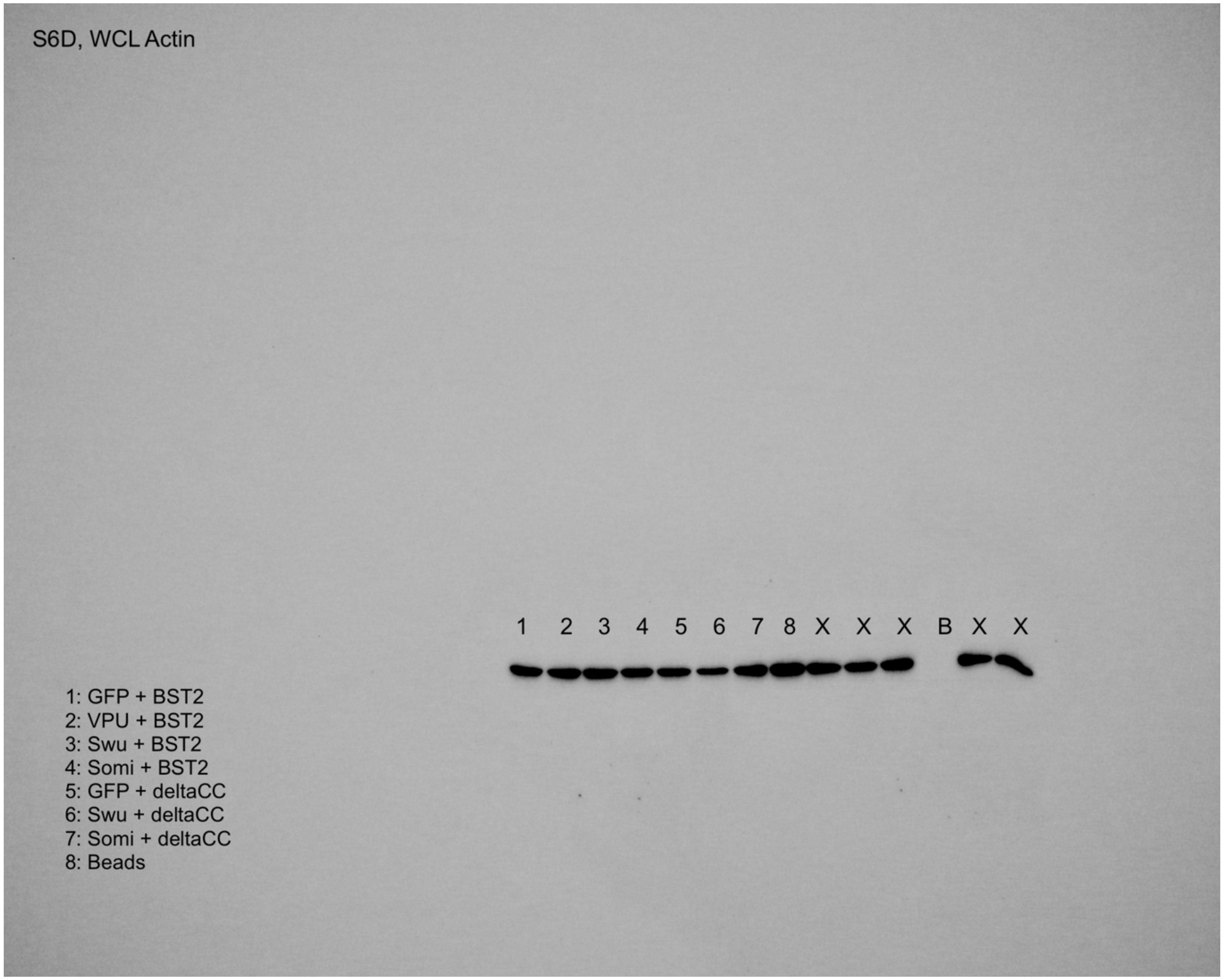
S6D, WCL Actin
1 2 3 4 5 6 7 8 X X X B X X
1: GFP + BST2
2: VPU + BST2
3: Swu + BST2
4: Somi + BST2
5: GFP + deltaCC
6: Swu + deltaCC
7: Somi + deltaCC
8: Beads

S6E, IP, Spike

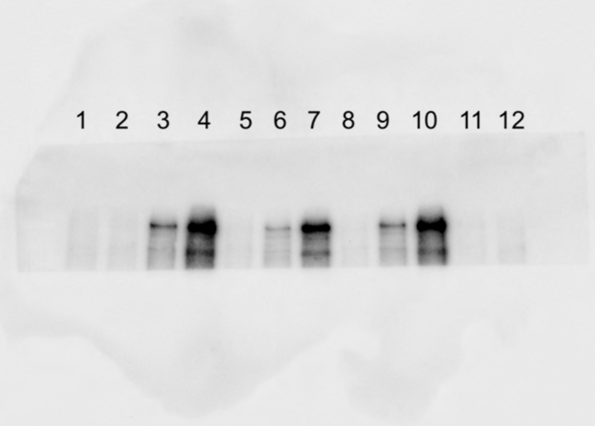

1: GFP + BST2
2: VPU + BST2
3: Swu + BST2
4: Somi + BST2
5: GFP + deltaEC1
6: Swu + deltaEC1
7: Somi + deltaEC1
8: GFP + EC2Ala
9: Swu + EC2Ala
10: Somi + EC2Ala
11: Beads
12: IgG

S6E, IP HA-BST2

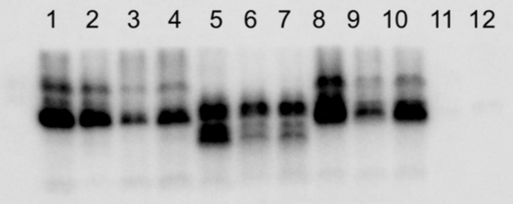

1: GFP + BST2
2: VPU + BST2
3: Swu + BST2
4: Somi + BST2
5: GFP + deltaEC1
6: Swu + deltaEC1
7: Somi + deltaEC1
8: GFP + EC2Ala
9: Swu + EC2Ala
10: Somi + EC2Ala
11: Beads
12: IgG

S6E, IP, GFP

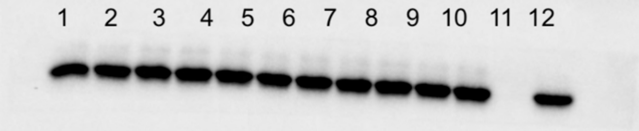

1: GFP + BST2
2: VPU + BST2
3: Swu + BST2
4: Somi + BST2
5: GFP + deltaEC1
6: Swu + deltaEC1
7: Somi + deltaEC1
8: GFP + EC2Ala
9: Swu + EC2Ala
10: Somi + EC2Ala
11: Beads
12: IgG

S6E, IP, VPU

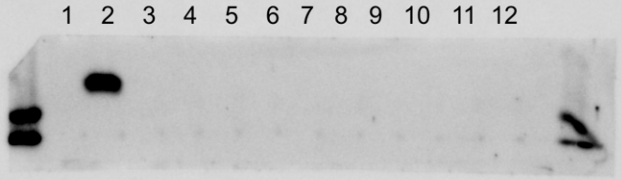

1: GFP + BST2
2: VPU + BST2
3: Swu + BST2
4: Somi + BST2
5: GFP + deltaEC1
6: Swu + deltaEC1
7: Somi + deltaEC1
8: GFP + EC2Ala
9: Swu + EC2Ala
10: Somi + EC2Ala
11: Beads
12: IgG

S6E WCL Spike

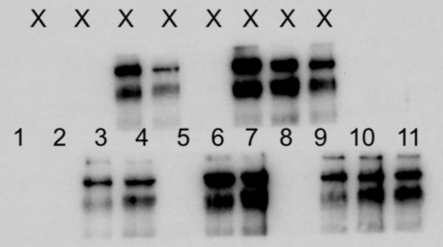

1: GFP + BST2
2: VPU + BST2
3: Swu + BST2
4: Somi + BST2
5: GFP + deltaEC1
6: Swu + deltaEC1
7: Somi + deltaEC1
8: GFP + EC2Ala
9: Swu + EC2Ala
10: Somi + EC2Ala
11: Beads

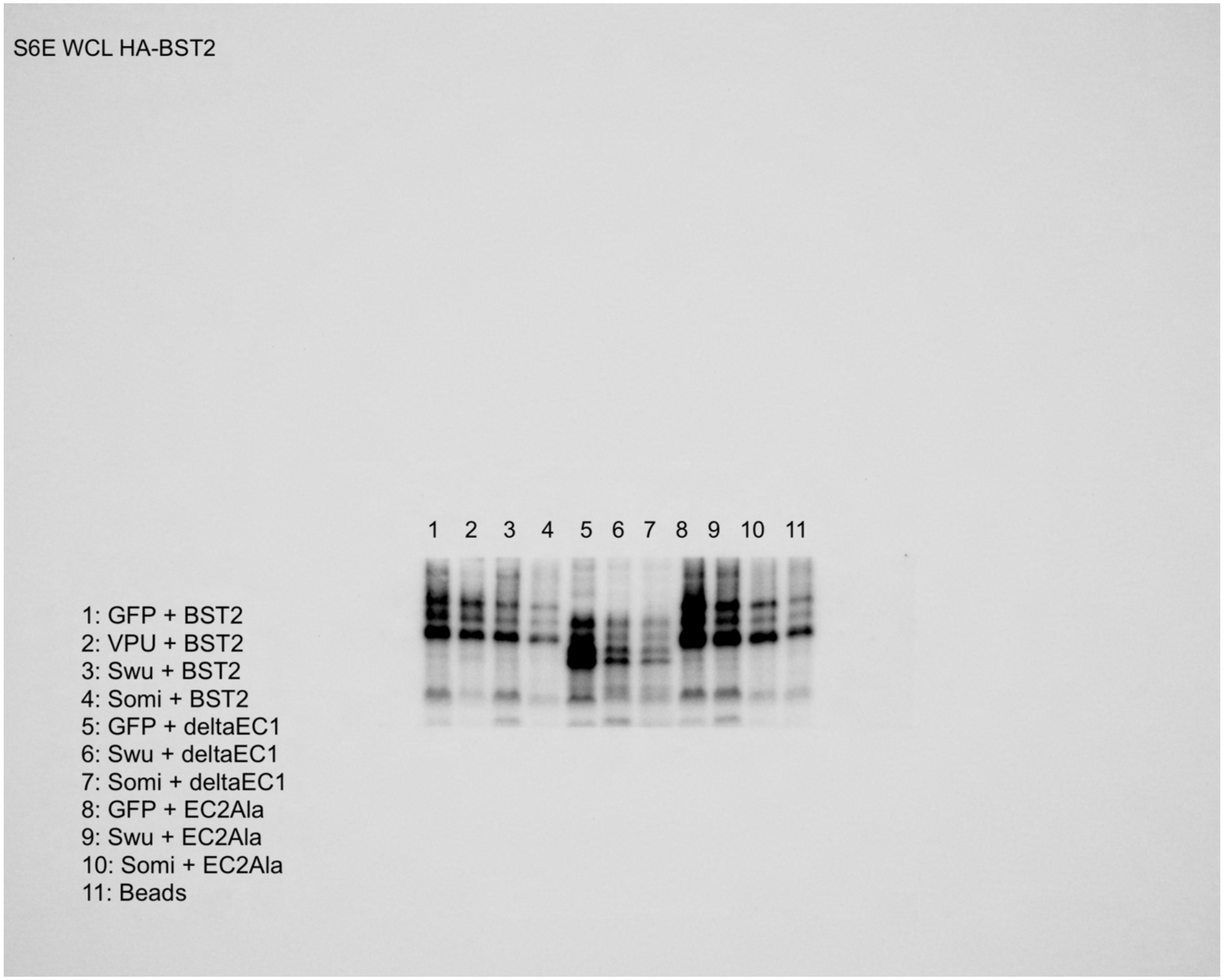
S6E WCL HA-BST2
1 2 3 4 5 6 7 8 9 10 11
1: GFP + BST2
2: VPU + BST2
3: Swu + BST2
4: Somi + BST2
5: GFP + deltaEC1
6: Swu + deltaEC1
7: Somi + deltaEC1
8: GFP + EC2Ala
9: Swu + EC2Ala
10: Somi + EC2Ala
11: Beads

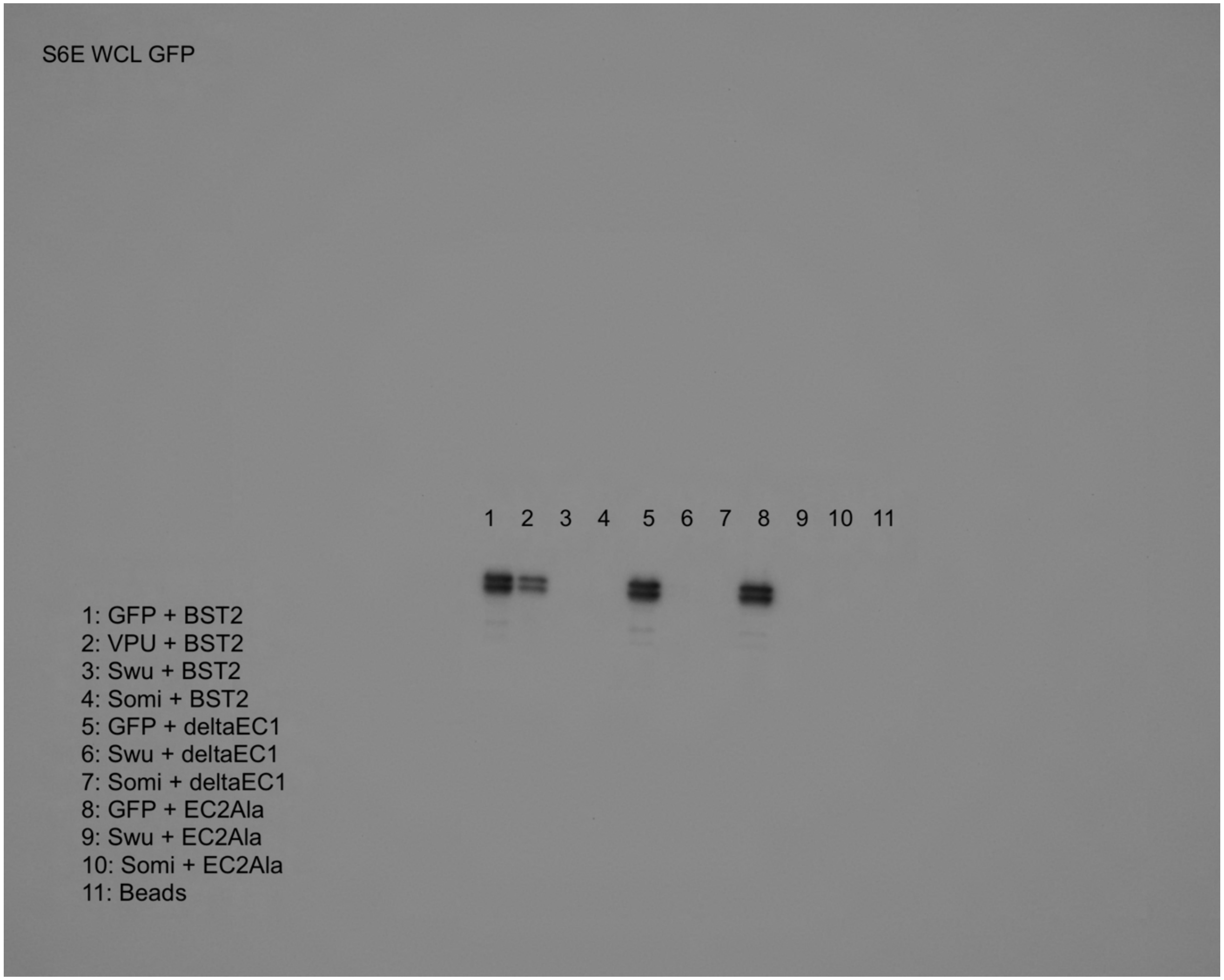
S6E WCL GFP
1 2 3 4 5 6 7 8 9 10 11
1: GFP + BST2
2: VPU + BST2
3: Swu + BST2
4: Somi + BST2
5: GFP + deltaEC1
6: Swu + deltaEC1
7: Somi + deltaEC1
8: GFP + EC2Ala
9: Swu + EC2Ala
10: Somi + EC2Ala
11: Beads

S6E WCL VPU

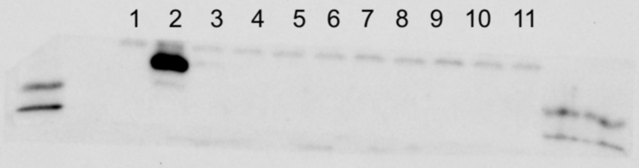

1: GFP + BST2
2: VPU + BST2
3: Swu + BST2
4: Somi + BST2
5: GFP + deltaEC1
6: Swu + deltaEC1
7: Somi + deltaEC1
8: GFP + EC2Ala
9: Swu + EC2Ala
10: Somi + EC2Ala
11: Beads

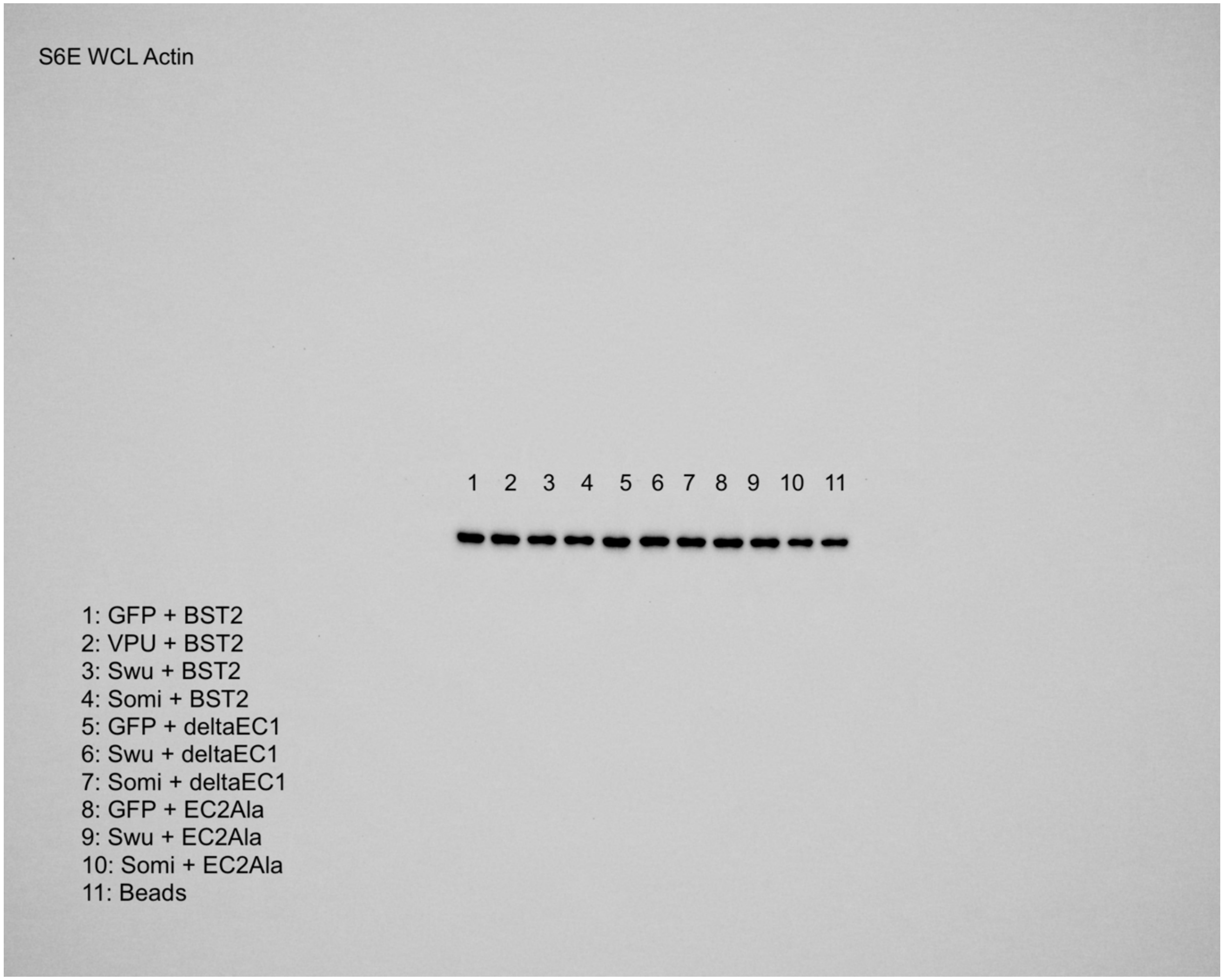
S6E WCL Actin
1 2 3 4 5 6 7 8 9 10 11
1: GFP + BST2
2: VPU + BST2
3: Swu + BST2
4: Somi + BST2
5: GFP + deltaEC1
6: Swu + deltaEC1
7: Somi + deltaEC1
8: GFP + EC2Ala
9: Swu + EC2Ala
10: Somi + EC2Ala
11: Beads
