# Supplementary material for: Mutations accumulated in the Spike of SARS-CoV-2 Omicron allow for more efficient counteraction of the restriction factor BST2/Tetherin
Source: PLoS Pathog. 2024 Jan 8;20(1):e1011912. doi: 10.1371/journal.ppat.1011912 (PMC10798645; doi:10.1371/journal.ppat.1011912)
Supplement: Attachment — Submitted filename: Response to reviewers.docx [file ppat.1011912.s009.docx]

**Reviewer #1**

The manuscript describes that SARS-CoV-2 S proteins can downregulate BST-2 through clathrin-dependent endocytosis by binding its multiple surfaces in the extracellular domain, which results in ubiquitination-dependent lysosomal degradation, and that the S-mediated BST-2 downregulation is more efficient in recent variants than in early isolates, suggesting the continuous adaptation of SARS-CoV-2 in human host cells. The experiments are well designed, and the results are of interest. Still, there are a few concerns that need to be addressed, as follows.

1) Lines 167-168: Although the authors’ state that ORF7a caused an enrichment of a lower molecular band of BST2, Fig. 3A does not show this phenotype (probably due to the overexposure?).

We thank the reviewer for this comment, since our explanation for ORF7a’s effect on BST2 may not have been clear. BST2 detection by western blot shows multiple bands, as evidenced in our figures. The native protein is predicted to migrate around 20-25 kDa and often bands above this molecular size are observed, reflecting post-translational modifications (PTMs) in the protein (mainly glycosylation). The lowest band of BST2 represents the molecule with only limited PTMs. This is the band that seems to be enriched in cells expressing SARS-CoV-2 ORF7a. We have clarified this concept in the text (lines 209-211) and have adjusted the brightness and contrast in the WB picture in Fig. 3A, so the enrichment of the lower band of BST2 by ORF7a is more evident.

2) Lines 289-290: The authors statement “deletion of the EC1 region drastically reduced the association with BST2 and S-wuhan and caused a partial loss in binding with S-omicron” is inconsistent with the results showing that deltaEC1 is still sensitive to degradation mediated by these S proteins (see Whole cell lysate in the same figure).

We agree with the reviewer that these results can be confusing. Despite the significant reduction in binding between the ΔEC1 mutant and S_wuhan_, this association is still detectable and appears to be sufficient to trigger the degradation of BST2. However, this is not observed when the interaction is completely lost (see S_wuhan_ and ΔCC compared to the control GFP and ΔCC co-expressing cells, Figure S6D). We have modified the text to avoid confusion. Also, in response to reviewer #2, we have modified figure 5 to better streamline the data and focus more on mapping the binding. The results originally presented in Figure 5 are now part of Supplementary Figure 6.

3) Fig. 7D and 7E: Because CD63 is the marker of the late endosomal and lysosomal membranes, its colocalization with BST-2 (Fig. 7D) should not be too different from that in lysotracker (Fig. 7E).

CD63 is a marker that is found in several membranes, including late endosomes, lysosomes, exosomes, multivesicular bodies and the plasma membrane (PMID: 18930046; 37198427). In HeLa cells (the cells used in Fig. 7), CD63 co-localizes extensively with the late endosomal marker Rab7 and also distributes at or close to the plasma membrane (PMID: 34282141; 16735575), which is consistent with our images. So, given its wide distribution, we were not surprised that the degree of co-localization between BST2 and CD63 in this cell line was lower than with the lysotracker. We are adding a comment in the text about CD63 subcellular distribution (lines 349-350). We are also providing quantifications for the co-localization of BST2 with each of these markers to better illustrate our findings.

**Reviewer #2**

In this manuscript, Shi et al. investigate the mechanism of BST2/tetherin counteraction by SARS-CoV-2 Spike and explore how the ongoing evolution of Spike influences this function. BST2 is an interferon stimulated gene (ISG) that restricts enveloped viruses by tethering them to cells upon budding. Several reports have demonstrated restriction of SARS-CoV-2 by BST2, but viral counteraction has focused on the role of ORF7a. SARS-CoV-1 Spike is known to counteract BST2, but little has been reported on the role of SARS-CoV-2 Spike. The authors show that SARS-CoV-2 infection increases BST2 expression in A549s, while higher MOI infection in cell lines overexpressing BST2 leads to BST2 degradation, an effect which may be stronger in more recent variants of concern. The authors demonstrate that this degradation occurs through lysosomal re-localization in a Clathrin-dependent manner, and suggest this is driven by multiple physical interactions on the extracellular surface.

Major concerns

1. Degradation of BST2 in the context of infection or S overexpression is only demonstrated in cell lines overexpressing BST2. To provide convincing evidence that Spike-mediated BST2 degradation is relevant to infection, revisions should demonstrate infection- and S-mediated degradation of endogenous BST2 in a lung epithelial cell line such as A549 or Calu-3, or ideally in primary lung epithelial cells. The non-BST2 overexpressing lanes in Figure S1 may be sufficient given proper exposure times. Cells may need to undergo treatment with exogenous IFN to induce suitable BST2 levels for blotting, which would need to be paired with higher MOI infection. This reviewer would also recommend inclusion of a remdesivir treated control in this experiment to demonstrate that BST2 downregulation is dependent on infection and de novo Spike expression and is not triggered by the challenge itself.

We agree with the reviewer that, despite generating cells stably expressing BST2, the degree of expression of BST2 could be higher than under conditions of IFN stimulation, and that using cells that naturally express BST2 at endogenous levels will be more physiologically relevant. Still, we want to stress that even though BST2 levels may be high in our system, SARS-CoV-2 is still capable of downregulating it and enhance virion release. Plus, we can still detect differences in the counteraction capabilities of different variants of concern under conditions of infection.

Following the reviewer’s advice, we infected A549-ACE2^+^ cells with three different MOIs of SARS-CoV-2 HK or Omicron (0.1, 1 and 5). 1 h later, cells were washed, fresh medium was added, and cells were treated with either DMSO or IFNα [1,000 U/mL as described by others (PMID: 24418563; 31213558)] to induce BST2. The degree of BST2 downregulation was examined 24 hours post-infection. We agree with the suggestion of including a control that allow us to discriminate the effect of productive and abortive infections on BST2. However, rather than using Remdesivir, we opted to use SARS-CoV-2 like particles instead. These virus-like particles (VLPs) have been extensively studied and characterized by others (PMID: 36129193; 36030323; 34735219; 33015007; 35632656). They harbor all the virus structural proteins but are deficient of the virus genome, which allows us to discriminate between effects caused by incoming Spike versus *de novo* Spike. SARS-CoV-2 VLPs were pelleted, analyzed by western blotting for their structural protein content and compared to pelleted virions of SARS-CoV-2 HK and Omicron of known titers, to estimate the amounts needed to infect with a comparable MOI of 1.

Similar to our experiments with cells engineered to constitutively express BST2, SARS-CoV-2 infection downregulated IFN-induced BST2. Consistent with our previous findings, Omicron caused a more dramatic downregulation of BST2 than HK. Hence, these observations demonstrate that SARS-CoV-2-mediated downregulation of BST2 is relevant to infection. These data are presented in Figure 2C and discussed in lines 171-191.

2. It would also be helpful to compare endogenous BST2 expression in non-overexpressing cell lines before and after IFN treatment to that shown in the overexpressing lines used in the paper, to contextualize how these lines compare to what we might expect to see in vivo.

As requested by the reviewer, we are now providing data comparing the levels of BST2 in A549-ACE2-BST2 (engineered to constitutively express BST2), parental A549-ACE2 treated with increasing concentrations of IFNα (100 U/mL, 1,000 U/mL and 10,000 U/mL) and HeLa cells, which express BST2 at endogenous levels and have been a gold standard cell line for studies on BST2. The data is presented in supplementary figure 1, and show that our A549-ACE2-BST2 cells have comparable BST2 levels to A549 cells treated with IFNα (lines 165-169).

3. The data regarding enhanced degradation by each successive variant of concern is complicated and the argument that this is due to enhanced affinity (Figure 4B) is not overly convincing. In the Figure 2 infection panels, Spike and infection levels vary despite uniform challenge due to the many dynamic factors influencing replication, Spike expression, and Spike localization/trafficking. These factors could easily impact the BST2 degradation phenotype. Indeed, the Figure 3C experiment that attempts to fix these issues by direct transfection of the different Spike plasmids shows a reduced effect, maybe only a two-fold difference between Wuhan and Omicron Spike proteins, and many of the other variants seem equivalent in contrast to the stepwise evolution argument (though this would require stats to determine). Looking at Spike expression relative to degradation (Figure 3B), Alpha even seems to perform worse on a per molecule basis. Given that no VOC evolved from any previous VOC, the evolution argument doesn’t really make conceptual sense either. This reviewer would recommend focusing the main text on looking at the Wuhan and Omicron Spike proteins only, relegating the other VOCs to the supplement. This should be paired with a thorough revision of the paper to remove any evolutionary argument, though this is something that could be speculated on in the Discussion. As the authors acknowledge, there were many changing selective pressures on SARS-CoV-2, including adaptive immunity, various non-pharmaceutical interventions, and a host of other ISGs and other facets of innate immunity. Discussion of any role BST2 may have had in SARS-CoV-2 Spike adaptation should be rooted in that context.

We agree with the reviewer that under conditions of infection, there may be multiple factors influencing the efficiency of BST2 downregulation by different strains, like changes in subcellular distribution of the Spike. Reviewer #3 had similar concerns. Following their advice, we are focusing on comparing early isolates (Wuhan/Hong Kong) with Omicron, and have moved the rest of the data on other variants of concern to supplementary figures. Therefore, we are now presenting a simplified version of the previous Fig 2. Here, we show side-by-side infections with HK and Omicron, where it is more obvious that, despite comparable levels of viral proteins, Omicron more efficiently counteracts BST2 (Fig. 2A). We are also providing statistical comparisons between S_wuhan_ vs S_omicron_ (Fig. 3C). Finally, we are now moving any evolutionary argument to the discussion (lines 456-471; 476-479, 488-492, 522-525) and we have changed the title of the manuscript to better reflect our focus on Omicron.

4. The use of affinity purification to claim a physical interaction between membrane proteins is similarly complicated, but the proper controls are included and acceptable (though this caveat should be mentioned). However, the claim that Omicron improves BST2 degradation through enhanced affinity is reliant on a minor change in band intensity using this approach in Figure 4B. This would be much more convincing if shown by an orthogonal method.

In response to the reviewer, we are mentioning the limitations of co-IPs of membrane proteins (lines 284-287), and we are toning down our conclusions on the differences in binding between S_wuhan_ and S_omicron_ with BST2 (lines 475-478). As noted by the reviewer, a significantly higher amount of S_omicron_ in the pulldown fraction of BST2 does not necessarily mean that this Spike has increased its affinity for BST2.

5. The domain mapping experiments in Figure 5 lead to somewhat mixed results. The delta Ct truncation seems to consistently improve binding and/or degradation while the delta EC and delta CC domains do seem more resistant to this degradation. The issue is that the results are presented so piecemeal and over so many different blots with different exposures, it is exceptionally hard to interpret. This section would be massively improved by a repeat of this experiment with only the most relevant constructs included and run on a single gel. The rest could be shunted to supplement.

We thank the reviewer for this suggestion. We have summarized the mapping data in two blots. One devoted to S_wuhan_ (Fig. 5B) and the other one devoted to S_omicron_ (Fig. 5C) since the goal of the original experiment was to show whether there were differences in the regions these Spikes use for binding to BST2. To fit all samples in one gel, we have only included one GFP control for the full-length BST2 protein. Since GFP does not bind to BST2, it is not expected to bind its truncation mutants. Also, we are only including the Vpu control for full-length BST2 and BST2 mutants in which Vpu binding is compromised (TfR-TM). Again, the extended data with all controls are now presented in supplementary figure S6.

6. The imaging data in Figures 6 & 7 needs to be quantified more convincingly. The BST2 distribution with the Omicron Spike protein looks distinctively different from that of the Wuhan Spike in Figure 6; the Omicron cells should be quantified alongside the Wuhan cells in Figure 6b.

We have taken additional pictures and performed quantifications as the reviewer recommended. We did not observe significant differences in the distribution of BST2 in cells expressing S_omicron_ and S_wuhan_. In both cases, BST2 appears clustered or sequestered and two phenotypes became evident: redistribution of BST2 to few discrete locations and in multiple puncta. We hypothesize that these differences in BST2 distribution represent intermediate steps in the process of downregulating BST2 (Fig. 6) (lines 499-502).

Furthermore, an objective metric is needed to quantify clustering, such as lacunarity (can be measured using FracLac in ImageJ) or even the percentage of pixels above some BST2 signal threshold in each cell. The BST2 signal seems to be quite bright in both the homogenous and clustered distributions, so this type of analysis could further emphasize that the decrease in BST2 levels occurs with the clustered localization.

Following the reviewer’s advice, we have calculated the clustering (lacunarity) of BST2 in cells expressing S_wuhan_, S_omicron_ or an empty vector (Fig. 6D). Consistent with our observations presented above, no significant differences in the clustering of BST2 is observed between S_omicron_ and S_wuhan_. This is discussed in lines 339-344.

For Figure 7, it is necessary to quantify colocalization between BST2 and the various compartment markers. It would also be beneficial to perform this analysis for the Omicron Spike to further support claims of differential BST2 interactions between the different Spike proteins. Microscopy showing BST2 localization to the lysosome in SARS-CoV-2 infected cells would be ideal, though the requirement for a BSL3 environment may make this challenging.

Following the reviewer’s advice, we are now including images for the subcellular distribution of BST2 in cells expressing S_omicron_. We are also providing Pearson’s correlation coefficients for the co-localization of BST2 with these markers in the presence of S_wuhan_ and S_omicron_ (Fig. 7). BST2 exhibits a very similar subcellular distribution pattern in the presence of either Spike, mainly co-localizing with lysosomal markers. This is now discussed in lines 351-360 and 502-504.

Reviewer #3: The studies by Shi and co-authors examined the interactions between BST2 and SARS-CoV-2, and demonstrated that BST2 reduces SARS-CoV-2 virion release, and the virus uses S protein to downregulate BST2. They also demonstrated a direct interaction between S and BST2, which routes BST2 for lysosomal degradation in a Clathtin- and Ubiquitin-dependent manner. The studies are extensive and largely well-done. There are a few minor issues:

1) the statement of SARS-CoV-2 variants, and that each variant is more efficient than the previously circulating strain at counteracting BST2. The data to support this statement is weak, and not very strong, also there are only very limited variants examined.

We have removed that argument based on this comment and concerns brought up by reviewer #2. So, following their recommendation, we are now focusing mainly on differences between early isolates (Wuhan/Hong Kong) and Omicron, which show the most striking differences in their ability to downregulate BST2 and enhance virus release. The data with the other variants of concern have been moved to the supplementary figures (supplementary figures 2 and 5).

2) Fig. 2. The authors have shown that omicron has a striking, much strongest phenotype in diminishing BST2. Omicrons are more infectious and likely replicate faster and infect more cells. Would the strong degradation of BST2 be caused by higher levels of replication rather than a stronger capacity of the variant virus *per se*? If we look at data on Fig. 3B, when S protein was used alone, Swuhan and Somicron have comparable levels of BST2. Please clarify the differences between the virus and the S protein in antagonizing BST2

We agree with the reviewer that each variant of concern has intrinsic differences in their kinetics for replication. We also worried that these differences would account for their distinct abilities to downregulate BST2. However, when we compared protein expression levels between HK and Omicron 24 h post-infection, we did not observe major fluctuations in their levels (Fig. 2A and 2C, left panel). So, at least 24 h post-infection, virus protein expression levels seem comparable. We confirmed this by looking at other viral proteins like ORF7a and NSP1 (not shown). In fact, when we express similar amounts of S_wuhan_ and S_omicron_, we still detect significantly more reduction in BST2 by S_omicron_ (see updated Fig. 3B where both Spikes are compared side-by-side). This effect has been consistently observed throughout the manuscript.

3) the statement “Whereas BST2 was initially recognized as an antiviral factor for its ability to tether HIV virions to the host cell membrane…”

This is not an accurate statement. BST2 was first identified to be a membrane protein downregulated by KSHV and HIV Vpu, suggesting that it is likely an antiviral factor (Bartee, E., McCormack, A., and Fruh, K. 2006, Quantitative membrane proteomics reveals new cellular targets of viral immune modulators. PLoS Pathog. 2, e107. 10.1371/journal.ppat.0020107). The work led to studies for its anti-HIV activity later (please see, Damme, et al. 2008, Cell Host & Microbe 3, 245-252). Please acknowledge and cite the earlier PLoS Pathogen paper.

We thank the reviewer for pointing this oversight. We have modified the text accordingly and added the citations (lines 60-63).

Reviewer #4: In this manuscript, Shi et al. endeavor to elucidate a potential mechanism by which SARS-CoV-2 counteracts host antiviral factor bone marrow stomal antigen (BST2). BST2 is a type I interferon stimulated gene (ISG) that restricts the release of budding enveloped virus particles including HIV-1, other retroviruses, arenaviruses, influenza viruses, herpesviruses, filoviruses, and several coronaviruses.

To elucidate whether BST2 also restricts SARS-CoV-2 virus and whether SARS-CoV-2, in turn, counteracts BST2, they performed various experiments using HEK-293T-ACE2+ and A549-ACE2+ cells stably expressing BST2 and different variants of SARS-CoV-2. The authors conclude that 1) BST2 is induced in response to SARS-CoV-2 infection and, in turn, SARS-coV-2 downregulates BST2 protein levels, 2) SARS-CoV-2 variants of concern have improved their anti-BST2 activity, 3) SARS-CoV-2 uses the Spike (S) protein to downregulate BST2, 4) multiple binding sites of BST2 are required for S-mediated downregulation of BST2, and 5) SARS-CoV-2 S promotes lysosomal degradation of BST2 in a Clathrin- and Ubiquitin-dependent manner. This conclusion is based on five key pieces of data:

1) Infection of A549-ACE2+ cells stably expressing BST2 with SARS-CoV-2 Hong Kong (HK) variant and measuring the BST2 expression 6 hours post infection (hpi) by RT-qPCR. Infection of HEK293T-ACE2+ cells stably expressing BST2 with SARS-CoV-2 HK variant and analysing protein levels of BST2 24 hpi by Western blot.

2) Infection of HEK293T-ACE2+ and A549-ACE2+ cells stably expressing BST2 with SARS-CoV-2 HK, Alpha, Beta, Delta, or Omicron variants and analysing protein levels of BST2 24 hpi by Western blot.

3) Transient transfection of HEK293T-ACE2+ cells stably expressing BST2 with constructs expressing individual SARS-CoV-2 proteins. BST2 protein levels were analysed by Western blot.

4) Generation of a series of BST2 truncation and point mutants which were used to co-transfect HEK293T cells with a plasmid expressing either S-Wuhan or S-Omicron. Co-IP was used to determine their ability to interact with S-Wuhan and S-Omicron.

5) Transfection of HEK293T-ACE2+ cells stably expressing BST2 with plasmid expressing either S-Wuhan or S-Omicron. 4 hours post transfection (hpt), cells were treated with either MG132 (proteasomal inhibitor), hydroxychloroquine (lysosomal inhibitor), or TAK-243 (ubiquitination inhibitor) for 44 hours and BST2 protein levels were analysed by Western blot. The role of vesicular trafficking in the downregulation of BST2 was studied using a dominant-negative mutant of Clathrin (AP180C) that was co-transfected with either S-Wuhan or S-Omicron into HEK293T-ACE2+ cells stably expressing BST2. BST2 protein levels were analysed by Western blot.

• Fig. 3.B: There is no explanation of what the red star represents in the HIV-1 Vpu blot. I assume it’s non-specific binding. This should be defined.

The reviewer is correct. The asterisks reflect a non-specific band in the blot. In response to reviewer #2, this panel has been moved to supplementary figure 5, but we have added this clarification to all figure legends where that non-specificity is detected.

• Fig. 5B-5E: Label S with Wuhan as you have labelled S-Omicron for a clarification and consistency.
In response to reviewer #2, we have modified figure 5 to better streamline the data. But following reviewer #4’s advice, we have labeled S-wuhan in all figures to keep consistency. Results previously presented in figure 5 are now in supplementary figure 6, where we have also updated this label.

• Fig. 5F: Symbols for a statistical significance are missing on the green (CTdel) and light purple (TfR-TM) bars. Please add.

Statistics have been added to the ΔCT and TfR-TM mutants (Fig. 5D).

• Fig. 6A: What are three rows in top panel and what are the two rows in bottom panel? I assume these are just replicates, but it needs to be clarified. Same for Fig. 6B, what are the two rows?

The three top rows in the previous version of figure 6A corresponded to cells transfected with empty vector, whereas the bottom two rows corresponded to cells transfected with S_wuhan_. To avoid confusion, we have split that figure in 3 panels (A: vector; B: S_wuhan_; C: S_omicron_). The idea is to show 2-3 pictures for each condition to illustrate reproducibility, but quantifications are provided (Fig. 6D).

• Fig. 7: Lack of consistency. Label S as in Fig. 6, i.e., S-Wuhan. As in Fig. 6 it is unclear what are the two rows in each panel (probably replicates, but this needs to be clarified).

We thank the reviewer for noticing this. The two rows in each panel represent independent replicates. This has been clarified in the figure legend (lines 907-908).

• Fig. 8A and B, and Fig. 9 (all panels): Lack of consistency. Label S-Wuhan as S-Omicron.

Thanks for noticing. We have labeled S_wuhan_ and S_omicron_ throughout the manuscript to keep a consistent labeling. The only exception is when panels are too busy. In this case, we use S_wu_ and S_omi_.
